# Design, Synthesis and Bioactivities of Novel Pyridyl Containing Pyrazole Oxime Ether Derivatives

**DOI:** 10.3390/molecules29122767

**Published:** 2024-06-11

**Authors:** Jie He, Beibei Zhou, Xinjuan Wang, Qi Chen, Xiaoqian Jiang, Ting Kong, Long Yao, Yingying Zhao, Rong Chen, Ying Xu, Hong Dai

**Affiliations:** 1 College of Chemistry and Chemical Engineering, Nantong University, Nantong 226019, China; hejie19960901@163.com (J.H.); zhoubeibei555@163.com (B.Z.); 15251030317@163.com (X.W.); 18361846329@163.com (Q.C.); jiangxq3466@163.com (X.J.); 18962733080@163.com (T.K.); z1373168932@163.com (Y.Z.); haiouggg@163.com (R.C.); 18020341673@163.com (Y.X.); 2 Analysis and Testing Center, Nantong University, Nantong 226019, China

**Keywords:** pyrazole oxime ether, pyridine, synthesis, biological activity

## Abstract

In this research, with an aim to develop novel pyrazole oxime ether derivatives possessing potential biological activity, thirty-two pyrazole oxime ethers, including a substituted pyridine ring, have been synthesized and structurally identified through ^1^H NMR, ^13^C NMR, and HRMS. Bioassay data indicated that most of these compounds owned strong insecticidal properties against *Mythimna separata*, *Tetranychus cinnabarinus*, *Plutella xylostella*, and *Aphis medicaginis* at a dosage of 500 μg/mL, and some title compounds were active towards *Nilaparvata lugens* at 500 μg/mL. Furthermore, some of the designed compounds had potent insecticidal effects against *M. separata*, *T. cinnabarinus*, or *A. medicaginis* at 100 μg/mL, with the mortalities of compounds **8a**, **8c**, **8d**, **8e**, **8f**, **8g**, **8o**, **8s**, **8v**, **8x**, and **8z** against *A. medicaginis*, in particular, all reaching 100%. Even when the dosage was lowered to 20 μg/mL, compound **8s** also expressed 50% insecticidal activity against *M. separata*, and compounds **8a**, **8e**, **8f**, **8o**, **8v**, and **8x** displayed more than 60% inhibition rates against *A. medicaginis*. The current results provided a significant basis for the rational design of biologically active pyrazole oxime ethers in future.

## 1. Introduction

**Figure 1:**
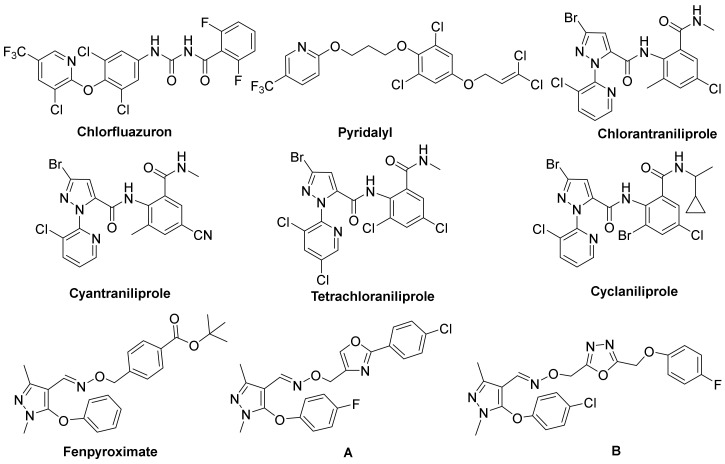
Structures of Chlorfluazuron, Pyridalyl, Chlorantraniliprole, Cyantraniliprole, Tetrachloraniliprole, Cyclaniliprole, Fenpyroximate and compounds **A**–**B**.

In recent years, compounds containing *N*-heterocycles are found to have wide application in pesticidal and medicinal fields [1,2,3,4,5]. As an important *N*-heterocycle, pyridine-based derivatives have drawn extensive interest owing to their diversified bioactivities, including insecticidal [6,7,8], fungicidal [9], herbicidal [10], antibacterial [11], and anticancer properties [12,13]. For example, Chlorfluazuron, Pyridalyl, Chlorantraniliprole, and Cyantraniliprole (Figure 1), commercial insecticides with a substituted pyridyl framework, demonstrated wonderful insecticidal properties against some important lepidopterous pests such as *Plutella xylostella*, *Mythimna separata* (Walker), and so on [14,15]. More recently, agrochemical scientists are focusing on the research of the structural modification of pyridine-based pesticides, and a number of bioactive compounds containing a pyridyl ring were reported [16,17,18,19], among which Tetrachloraniliprole and Cyclaniliprole were the typical commercial pesticides [20]. Therefore, the wide spread use of pyridine compounds as a skeleton in the field of crop protection establishes the pyridyl unit as a vital structural class.

In addition, pyrazole oxime ethers are also significant *N*-heterocyclic compounds, which act as an active pharmacophore during the development of new agrochemicals and drugs [21,22]. Many pyrazole oxime ether derivatives are reported to display various pesticidal and pharmacological activities involving fungicidal [23], insecticidal [24], anti-TMV [25], and antiproliferative effects [26]. For instance, Fenpyroximate (Figure 1) was an outstanding acaricide invented by Nihon Nohyaku in 1991. In the past few decades, Fenpyroximate has been extensively applied in protecting certain kinds of crops because of its efficient insecticidal activities towards agricultural mites like *Polyphagotarsonemus latus* (Banks), *Tetranychus urticae* (Koch), and so on [27]. Thereafter, many studies about the structural derivation of Fenpyroximate were performed. Recently, Wang et al. found that oxazole-containing pyrazole oxime ether derivative **A** (Figure 1) had a broad spectrum of insecticidal and fungicidal properties [28]. Sun et al. reported pyrazole oxime ether **B** (Figure 1) with an aryloxy-linked 1,3,4-oxadiazole moiety displaying satisfactory insecticidal effects [29]. This greatly promoted the development of bioactive pyrazole oxime ether molecules.

**Figure 2:**
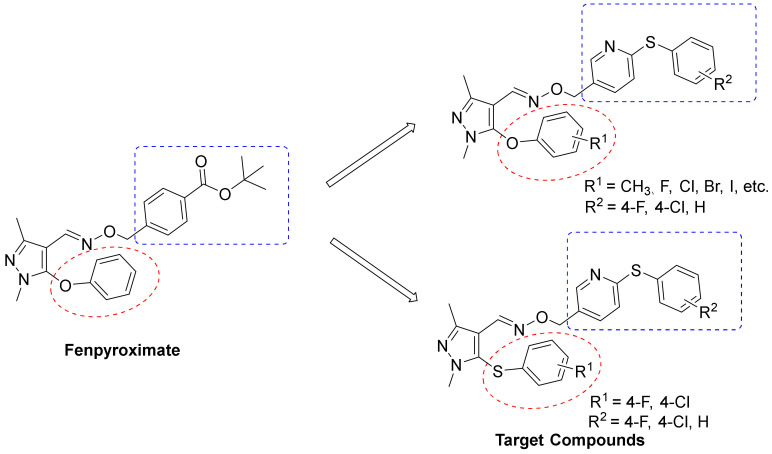
Design of the target compounds.

Inspired by the aforementioned reports, we hypothesized that the introduction of prominent pyridine pharmacophore into a pyrazole oxime ether unit might result in some novel potentially insecticidal agents. Herein, we describe the design and preparation of a series of new pyrazole oxime ether derivatives carrying pyridyl moiety (Figure 2). Furthermore, the insecticidal activities of the newly obtained pyrazole oxime ethers were systematically assessed and discussed.

## 2. Results and Discussion

### 2.1. Chemistry

**Scheme 1:**
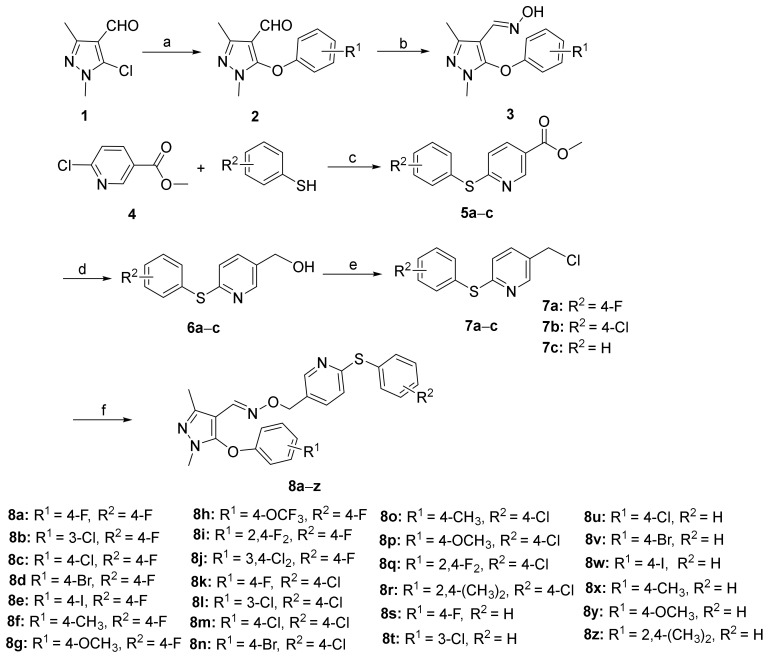
Synthesis of the designed compounds **8a**–**z**. Reagents and conditions: (**a**) substituted phenol, KOH, DMF, 50 °C, 3–4 h, 110 °C, 5–8 h, 49–73%; (**b**) NH_2_OH·HCl, KOH, CH_3_OH, reflux, 3–4 h, 55–69%; (**c**) substituted thiophenol, K_2_CO_3_, DMF, 55 °C, 3–4 h, 110 °C, 5–6 h, 66–71%; (**d**) LiAlH_4_, THF, 0 °C, 2–3 h, 33–42%; (**e**) SOCl_2_, CH_2_Cl_2_, 0 °C, 2–3 h, 71–78%; (**f**) compound **3**, K_2_CO_3_, DMF, 55 °C, 4–6 h, 43–73%.

**Scheme 2:**
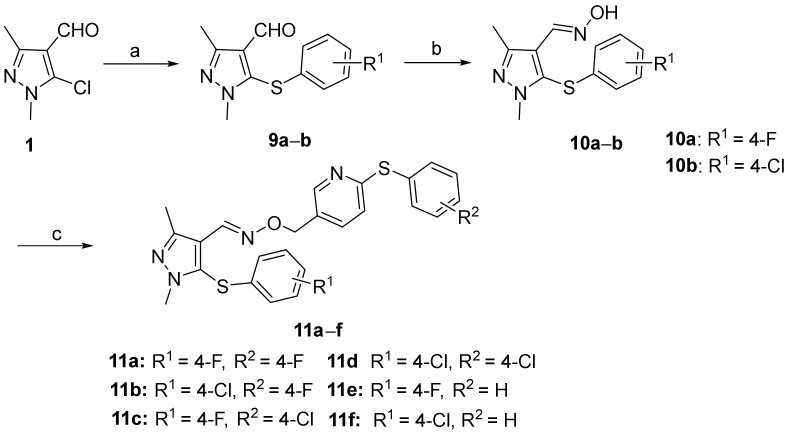
Synthesis of the designed compounds **11a**–**f**. Reagents and conditions: (**a**) substituted thiophenol, KOH, DMF, H_2_O, 55 °C, 2–3 h, 100 °C, 6–8 h, 47–53%; (**b**) NH_2_OH·HCl, KOH, CH_3_OH, r.t., 10 min, reflux, 3 h, 62–65%; (**c**) compounds **7a**–**c**, K_2_CO_3_, DMF, 55 °C, 5–6 h, 42–74%.

The synthetic pathways for the title compounds **8a**–**z** and **11a**–**f** are illustrated in Scheme 1 and Scheme 2, respectively. The crucial intermediates **3** were efficiently synthesized through a two-step process, selecting 5-chloro-1,3-dimethyl-1*H*-pyrazole-4-carbaldehyde (**1**) as the starting material. The reaction of intermediate **1** with diverse substituted phenols or substituted thiophenols was conducted under alkaline conditions to produce compounds **2** or **9a**,**b** smoothly. Next, intermediates **2** or **9a**,**b** were reacted with hydroxylamine hydrochloride, utilizing potassium hydroxide as the base to afford the key intermediates **3** or **10a**,**b** conveniently. The important synthons 5-chloromethyl-2-(substituted phenylthio)pyridine (**7a**–**c**) were prepared from commercially available methyl 6-chloronicotinate (**4**). Compound **4** underwent an aromatic nucleophilic substitution with substituted thiophenols, choosing potassium carbonate as the deacid reagent and DMF as the solvent to generate intermediates **5a**–**c** in good yields. The further reduction reaction with LiAlH_4_ obtained compounds **6a**–**c** successfully. Compounds **6a**–**c** were then converted into the significant intermediates **7a**–**c** by treatment with thionyl chloride. Subsequently, compounds **7a**–**c** were treated with the important intermediates **3** or **10a**,**b** in *N* and *N*-dimethylformamide, utilizing potassium carbonate as the acid scavenger to obtain the desired compounds **8a**–**z** and **11a**–**f** in satisfactory yields. Additionally, the structures of the targeted compounds **8a**–**z** and **11a**–**f** were effectively determined by ^1^H NMR, ^13^C NMR and HRMS.

### 2.2. Biological Activities

The designed compounds **8a**–**z** and **11a**–**f** were screened for insecticidal activities towards *Mythimna separata*, *Tetranychus cinnabarinus*, *Plutella xylostella*, *Aphis medicaginis* and *Nilaparvata lugens*, and Tolfenpyrad, Fenpyroximate, Pyridalyl, Imidacloprid, and Abamectin were selected as reference drugs, respectively. As depicted in Table 1, most of the desired compounds had satisfactory insecticidal properties against *M. separata* at the dosage of 500 μg/mL, and the larvicidal effects of compounds **8a**–**f**, **8h**, **8j**, **8l**, **8n** and **8s**–**z** against *M. separata* were all 100%, respectively, which were comparable to that of Tolfenpyrad (100%). At 100 μg/mL, some targeted compounds also demonstrated interesting insecticidal activities against *M. separata*, for example, compounds **8a**, **8b**, **8d**, **8n**, **8s**, **8t**, **8u**, **8v**, **8w**, **8x**, and **8z** owned 100%, 70%, 100%, 60%, 100%, 60%, 70%, 100%, 50%, 60%, and 50% inhibition rates, which were equal to or higher than Tolfenpyrad (50%). Even when the dosage reached 20 μg/mL, compound **8s** still possessed 50% insecticidal effect towards *M. separata*, which was better than Tolfenpyrad (40%). Among the aryloxy derivatives, when R^2^ was 4-fluoro, the substituents (R^1^) on the phenyl ring were 4-fluoro (**8a**) and 4-bromo (**8d**), and it was more helpful to improve insecticidal properties towards *M. separata* at 100 μg/mL than other substituents; when R^2^ was 4-chloro, compound **8n** (R^1^ = 4-Br) had better insecticidal activity against *M. separata* than compounds **8k**–**m** and **8o**–**r** at 100 μg/mL; and when R^2^ was H, the substituents (R^1^) on the phenyl ring were 4-fluoro (**8s**), 4-chloro (**8u**) and 4-bromo (**8v**), and it was more favorable to increase larvicidal activities towards *M. separata* at 100 μg/mL than other substituents. Among the arylthio derivatives, the mortalities of compounds **11d** (R^1^ = 4-Cl, R^2^ = 4-Cl) and **11e** (R^1^ = 4-F, R^2^ = H) against *M. Separata* were 80% and 70% at 500 μg/mL, which were much higher than those of **11a**–**c** and **11f**. From the list illustrated in Table 1, we found that most title compounds displayed wonderful inhibitory effects towards *T*. *cinnabarinus* and *P*. *xylostella* at 500 μg/mL, and compounds **8a**, **8c**, **8i**, **8k**, and **8m** demonstrated 80% to 100% inhibition rates towards *T*. *cinnabarinus* at the dosage of 100 μg/mL, which were near to that of Fenpyroximate. Table 2 showed that most of the prepared compounds possessed excellent insecticidal properties against *A*. *medicaginis* at 500 μg/mL, and some of them still had 80% to 100% mortalities against *A*. *medicaginis* at a lower dosage of 100 μg/mL. Even at 20 μg/mL, some compounds were active towards *A*. *medicaginis*, with the inhibition rates of compounds **8a**, **8c**–**f**, **8o**, **8v**, **8x**, and **8z**, especially, being 50% to 70%. From the structure–insecticidal activity data (Table 2), we can see that at a dosage of 500 μg/mL, the aryloxy derivatives **8a**-**z** exhibited much better insecticidal effects against *A*. *medicaginis* than corresponding arylthio analogues **11a**-**f**. Among the aryloxy derivatives, when R^2^ is 4-fluoro, the substituents (R^1^) on the phenyl ring were 4-fluoro (**8a**), 4-chloro (**8c**) and 4-methyl (**8f**), it was more beneficial to ameliorate inhibitory activity towards *A*. *medicaginis* at 20 μg/mL than corresponding analogues **8k** (R^1^ = 4-F, R^2^ = 4-Cl), **8s** (R^1^ = 4-F, R^2^ = H), **8m** (R^1^ = 4-Cl, R^2^ = 4-Cl), **8u** (R^1^ = 4-Cl, R^2^ = H), **8o** (R^1^ = 4-CH_3_, R^2^ = 4-Cl), and **8x** (R^1^ = 4-CH_3_, R^2^ = H). Additionally, some of these compounds represented perfect inhibition effects against *N*. *lugens* at 500 μg/mL, for instance, compounds **8a**, **8c**–**i**, **8s**, **8u**, **8w**, **8x** and **8z** held 100%, 100%, 100%, 100%, 100%, 100%, 100%, 80%, 100%, 100%, 90%, 100%, and 80% mortalities against *N*. *lugens*, which were equal to that of Abamectin. The data provided in Table 1 and Table 2 also indicated that compounds **8a** (R^1^ = 4-F, R^2^ = 4-F), **8c** (R^1^ = 4-Cl, R^2^ = 4-F), **8d** (R^1^ = 4-Br, R^2^ = 4-F), **8s** (R^1^ = 4-F, R^2^ = H), **8v** (R^1^ = 4-Br, R^2^ = H), **8x** (R^1^ = 4-CH_3_, R^2^ = H), and **8z** (R^1^ = 2,4-(CH_3_)_2_, R^2^ = H) showed extensive insecticidal properties towards the tested pests, such as *M. separata*, *T. cinnabarinus*, *P*. *xylostella*, *A*. *medicaginis*, and *N*. *lugens*, which might be chosen as potential insecticidal agent candidates for deeper structural optimization and bioactivity research. All the aforementioned data suggested that the biological activity spectra of pyrazole oxime ether derivatives were vitally improved through importing the significant pyridine unit.

## 3. Materials and Methods

### 3.1. Chemistry

All chemical agents were chemically pure and treated by standard methods. ^1^H NMR, and ^13^C NMR spectra were screened through a Bruker AV400 spectrometer (Bruker, Billerica, MA, USA) in the solvent of CDCl_3_, and TMS was used as the internal reference. High-resolution mass spectra (HRMS) were collected by a Waters Xevo G2-XS Q-TOF mass spectrometer (Waters, Milford, CT, USA). The melting point was tested through an X-4 binocular microscope melting point detector (Beijing Tech Instrument Co., Beijing, China) and uncorrected. Intermediates **1**–**3** were prepared by reported procedures [30].

#### 3.1.1. General Approach to Preparation of **5a**–**c**

To a well-stirred solution of substituted thiophenol (55 mmol) in DMF (80 mL), potassium carbonate was added (110 mmol, 15.18 g) at room temperature, and the mixture was kept at 55 °C for another 3–4 h. Next, compound **4** (36 mmol, 6.18 g) was added thereto. The reaction mixture was heated to 110 °C for 5–6 h. After cooling, the solution was poured into ice water (100 mL). The generating precipitates were filtrated to obtain intermediates **5a**–**c**.

*Methyl 6-(4-fluorophenylthio)nicotinate* (**5a**): colorless solid, yield 66%, m.p. 102–104 °C; ^1^H NMR (400 MHz, CDCl_3_) *δ* 8.96 (s, 1H, Py-H), 8.00 (d, *J* = 8.40 Hz, 1H, Py-H), 7.66–7.54 (m, 2H, Ar-H), 7.15 (t, *J* = 8.60 Hz, 2H, Ar-H), 6.85 (d, *J* = 8.40 Hz, 1H, Py-H), 3.89 (s, 3H, OCH_3_); ^13^C NMR (101 MHz, CDCl_3_) *δ* 167.18, 165.61, 162.51, 150.81, 137.72, 131.21, 124.64, 122.10, 119.61, 117.30, 52.33. HRMS calcd for C_13_H_11_FNO_2_S [M + H]^+^ 264.0495, found 246.0499.

*Methyl 6-(4-chlorophenylthio)nicotinate* (**5b**): colorless solid, yield 71%, m.p. 97–99 °C; ^1^H NMR (400 MHz, CDCl_3_) *δ* 8.97 (s, 1H, Py-H), 8.02 (d, *J* = 8.50 Hz, 1H, Py-H), 7.53 (m, 2H, Ar-H), 7.15 (t, *J* = 8.60 Hz, 2H, Ar-H), 6.85 (d, *J* = 8.40 Hz, 1H, Py-H), 3.89 (s, 3H, OCH_3_); ^13^C NMR (101 MHz, CDCl_3_) *δ* 166.53, 165.57, 150.87, 137.43, 136.69, 136.23, 130.12, 127.92, 122.27, 119.93, 52.63. HRMS calcd for C_13_H_11_ClNO_2_S [M + H]^+^ 280.0199, found 280.0203.

*Methyl 6-(phenylthio)nicotinate (***5c***)*: colorless solid, yield 69%, m.p. 72–74 °C; ^1^H NMR (400 MHz, CDCl_3_) *δ* 8.98 (d, *J* = 2.20 Hz, 1H, Py-H), 8.00–7.98 (m, 1H, Py-H), 7.63–7.59 (m, 2H, Ar-H), 7.51–7.40 (m, 3H, Ar-H), 6.84 (d, *J* = 8.50 Hz, 1H, Py-H), 3.89 (s, 3H, OCH_3_); ^13^C NMR (101 MHz, CDCl_3_) *δ* 167.69, 165.67, 150.76, 137.36, 135.53, 129.97, 129.91, 129.40, 121.93, 119.74, 52.32. HRMS calcd for C_13_H_12_NO_2_S [M + H]^+^ 246.0589, found 246.0590.

#### 3.1.2. General Approach to Preparation of **6a**–**c**

To a cold (0 °C) solution of compound **5** (30 mmol) in THF (50 mL), LiAlH_4_ (60 mmol, 2.28 g) was added in portions, and the mixture was kept at 0 °C for another 2–3 h. When the reaction was achieved, ice water (2 mL) was added dropwise slowly thereto. After filtration, the filter liquor was evaporated under reduced pressure to provide a residue, which was further purified through silica gel column chromatography, selecting a mixture of ethyl acetate/petroleum ether (1:2, *v*/*v*) as the eluent to give intermediates **6a**–**c**.

*[6-(4-Fluorophenylthio)pyridin-3-yl]methanol* (**6a**): yellow oil, yield 37%; ^1^H NMR (400 MHz, CDCl_3_) *δ* 8.24 (s, 1H, Py-H), 7.60–7.40 (m, 3H, Py-H and Ar-H), 7.07 (t, *J* = 8.70 Hz, 2H, Ar-H), 6.77 (d, *J* = 8.20 Hz, 1H, Py-H), 4.55 (s, 2H, CH_2_); ^13^C NMR (101 MHz, CDCl_3_) *δ* 164.61, 162.12, 159.95, 148.02, 137.18, 136.34, 133.47, 125.97, 121.08, 117.07, 61.54. HRMS calcd for C_12_H_11_FNOS [M + H]^+^ 236.0545, found 236.0550.

*[6-(4-Chlorophenylthio)pyridin-3-yl]methanol* (**6b**): yellow oil, yield 42%; ^1^H NMR (400 MHz, CDCl_3_) *δ* 8.30 (d, *J* = 2.30 Hz, 1H, Py-H), 7.50 (d, *J* = 8.30 Hz, 1H, Py-H), 7.42 (d, *J* = 8.50 Hz, 2H, Ar-H), 7.33 (d, *J* = 8.50 Hz, 2H, Ar-H), 6.87 (d, *J* = 8.20 Hz, 1H, Py-H), 5.44 (s, 1H, OH), 4.57 (s, 2H, CH_2_); ^13^C NMR (101 MHz, CDCl_3_) *δ* 159.00, 147.80, 136.68, 135.85, 135.49, 133.93, 129.96, 121.91, 61.59. HRMS calcd for C_12_H_11_ClNOS [M + H]^+^ 252.0250, found 252.0255.

*[6-(Phenylthio)pyridin-3-yl]methanol* (**6c**): yellow oil, yield 33%; ^1^H NMR (400 MHz, CDCl_3_) *δ* 8.28 (d, *J* = 2.20 Hz, 1H, Py-H), 7.53–7.51 (m, 2H, Py-H and Ar-H), 7.48–7.45 (m, 1H, Ar-H), 7.43–7.34 (m, 3H, Ar-H), 6.83 (d, *J* = 8.20 Hz, 1H, Py-H), 4.57 (s, 2H, CH_2_), 3.74 (s, 1H, OH); ^13^C NMR (101 MHz, CDCl_3_) *δ* 160.26, 148.11, 136.29, 134.75, 133.27, 131.00, 129.75, 129.18, 121.52, 61.81.HRMS calcd for C_12_H_12_NOS [M + H]^+^ 218.0640, found 218.0643.

#### 3.1.3. General Approach to Preparation of **7a**–**c**

To a cold (0 °C) solution of compound **6** (30 mmol) in CH_2_Cl_2_ (60 mL), thionyl chloride was added dropwise (60 mmol, 7.14 g), and then the mixture was stirred at 0 °C for an additional 2–3 h. After the reaction was achieved, 20 mL of water was added slowly thereto. The resulting solution was extracted with CH_2_Cl_2_ (3 × 30 mL). The collected extracts were dried on anhydrous sodium sulfate, filtered, and condensed to obtain the key intermediates **7a**–**c**.

*5-Chloromethyl-2-(4-fluorophenylthio)pyridine* (**7a**): yellow oil, yield 73%; ^1^H NMR (400 MHz, CDCl_3_) *δ* 8.40 (s, 1H, Py-H), 7.64–7.54 (m, 2H, Ar-H), 7.52 (d, *J* = 8.30 Hz, 1H, Py-H), 7.14 (t, *J* = 8.60 Hz, 2H, Ar-H), 6.86 (d, *J* = 8.30 Hz, 1H, Py-H), 4.52 (s, 2H, CH_2_); ^13^C NMR (101 MHz, CDCl_3_) *δ* 164.79, 162.30, 149.24, 137.55, 129.54, 125.48, 120.80, 117.13, 42.94. HRMS calcd for C_12_H_10_ClFNS [M + H]^+^ 254.0207, found 254.0212.

*5-Chloromethyl-2-(4-chlorophenylthio)pyridine* (**7b**): yellow oil, yield 78%; ^1^H NMR (400 MHz, CDCl_3_) *δ* 8.39 (s, 1H, Py-H), 7.59–7.45 (m, 3H, Py-H and Ar-H), 7.37 (d, *J* = 8.40 Hz, 2H, Ar-H), 6.91 (d, *J* = 8.30 Hz, 1H, Py-H), 4.50 (s, 2H, CH_2_); ^13^C NMR (101 MHz, CDCl_3_) *δ* 161.03, 149.37, 137.20, 136.28, 135.64, 129.93, 121.28, 42.94. HRMS calcd for C_12_H_10_Cl_2_NS [M + H]^+^ 269.9911, found 269.9915.

*5-Chloromethyl-2-(phenylthio)pyridine* (**7c**): colorless oil, yield 71%; ^1^H NMR (400 MHz, CDCl_3_) *δ* 8.38 (d, *J* = 2.40 Hz, 1H, Py-H), 7.58–7.56 (m, 2H, Py-H and Ar-H), 7.47–7.40 (m, 4H, Ar-H), 6.84 (d, *J* = 8.30 Hz, 1H, Py-H), 4.49 (s, 2H, CH_2_); ^13^C NMR (101 MHz, CDCl_3_) *δ* 162.08, 149.26, 137.13, 135.16, 130.44, 129.81, 129.45, 129.43, 121.04, 43.06. HRMS calcd for C_12_H_11_ClNS [M + H]^+^ 236.0301, found 236.0305.

#### 3.1.4. General Approach to Preparation of Target Compounds **8a**–**z**

To a solution of compound **3** (11 mmol), potassium carbonate (20 mmol, 2.76 g) in DMF (30 mL), compound **7** (10 mmol) was added, and then the mixture was stirred at 55 °C for 4–6 h. After the reaction was completed, 30 mL of water was added thereto. Next, the mixture was extracted with CH_2_Cl_2_ (3 × 20 mL). The gathered extracts were further dried on anhydrous sodium sulfate, filtered, and concentrated to generate the crude product, which was then separated by silica gel column chromatography, choosing a mixture of ethyl acetate/petroleum ether (1:4, *v*/*v*) as the eluent to afford the targeted compounds **8a**–**z** successfully, and the corresponding spectral data of compounds **8a**–**z** are illustrated below. ^1^H NMR and ^13^C NMR spectra are presented in Supplementary Materials.

*5-(4-Fluorophenoxy)-1,3-dimethyl-1H-pyrazole-4-carbaldehyde O-{[6-(4-fluorophenylthio)pyridin-3-yl]methyl}oxime* (**8a**): yellow oil, yield 61%; ^1^H NMR (400 MHz, CDCl_3_) *δ* 8.29 (d, *J* = 2.30 Hz, 1H, Py-H), 7.71 (s, 1H, CH=N), 7.53–7.50 (m, 2H, Ar-H), 7.33–7.31 (m, 1H, Py-H), 7.06 (t, *J* = 8.60 Hz, 2H, Ar-H), 6.94–6.90 (m, 2H, Ar-H), 6.80–6.73 (m, 3H, Py-H and Ar-H), 4.85 (s, 2H, CH_2_), 3.55 (s, 3H, CH_3_), 2.28 (s, 3H, CH_3_); ^13^C NMR (101 MHz, CDCl_3_) *δ* 164.59, 162.11, 160.83, 159.86, 157.45, 152.57, 149.74, 147.64, 146.92, 140.98, 137.36, 129.58, 125.96, 120.50, 116.95, 116.59, 116.36, 99.83, 72.81, 34.18, 14.62. HRMS calcd for C_24_H_21_F_2_N_4_O_2_S [M + H]^+^ 467.1353, found 467.1352.

*5-(3-Chlorophenoxy)-1,3-dimethyl-1H-pyrazole-4-carbaldehyde O-{[6-(4-fluorophenylthio)-pyridin-3-yl]methyl}oxime* (**8b**): yellow oil, yield 62%; ^1^H NMR (400 MHz, CDCl_3_) *δ* 8.30 (d, *J* = 2.30 Hz, 1H, Py-H), 7.74 (s, 1H, CH=N), 7.56–7.53 (m, 2H,Ar-H), 7.33–7.31 (m, 1H, Py-H), 7.18 (t, *J* = 8.20 Hz, 1H, Ar-H), 7.09 (t, *J* = 8.60 Hz, 2H, Ar-H), 7.04–7.01 (m, 1H, Ar-H), 6.84 (s, 1H,Py-H), 6.77–6.72 (m, 2H, Ar-H), 4.86 (s, 2H, CH_2_), 3.57 (s, 3H, CH_3_), 2.30 (s, 3H, CH_3_); ^13^C NMR (101 MHz, CDCl_3_) *δ* 164.64, 162.15, 160.85, 157.07, 149.69, 147.05, 146.78, 140.83, 137.31, 135.39, 130.80, 129.56, 125.99, 123.94, 120.56, 116.99, 116.78, 115.82, 113.53, 100.14, 72.87, 34.28, 14.54. HRMS calcd for C_24_H_21_ClFN_4_O_2_S [M + H]^+^ 483.1058, found 483.1062.

*5-(4-Chlorophenoxy)-1,3-dimethyl-1H-pyrazole-4-carbaldehyde O-{[6-(4-fluorophenylthio)-pyridin-3-yl]methyl}oxime* (**8c**): yellow oil, yield 59%; ^1^H NMR (400 MHz, CDCl_3_) *δ* 8.34 (d, *J* = 2.20 Hz, 1H, Py-H), 7.75 (s, 1H, CH=N), 7.60–7.56 (m, 2H,Ar-H), 7.35–7.33 (m, 1H, Py-H), 7.27–7.23 (m, 2H, Ar-H), 7.13 (t, *J* = 8.60 Hz, 2H, Ar-H), 6.81–6.79 (m, 3H, Py-H and Ar-H), 4.89 (s, 2H, CH_2_), 3.59 (s, 3H, CH_3_), 2.32 (s, 3H, CH_3_); ^13^C NMR (101 MHz, CDCl_3_) *δ* 164.71, 162.22, 160.90, 155.17, 149.62, 147.07, 140.95, 137.41, 129.93, 129.61, 128.75, 125.89, 120.65, 117.06, 116.84, 116.57, 100.01, 72.88, 34.27, 14.59. HRMS calcd for C_24_H_21_ClFN_4_O_2_S [M + H]^+^ 483.1058, found 483.1062.

*5-(4-Bromophenoxy)-1,3-dimethyl-1H-pyrazole-4-carbaldehyde O-{[6-(4-fluorophenylthio)-pyridin-3-yl]methyl}oxime* (**8d**): yellow oil, yield 66%; ^1^H NMR (400 MHz, CDCl_3_) *δ* 8.33 (d, *J* = 2.20 Hz, 1H, Py-H), 7.74 (s, 1H, CH=N), 7.58–7.54 (m, 2H, Ar-H), 7.37–7.32 (m, 3H, Ar-H and Py-H), 7.11 (t, *J* = 8.60 Hz, 2H, Ar-H), 6.80 (d, *J* = 8.20 Hz, 1H, Py-H), 6.74 (d, *J* = 9.00 Hz, 2H, Ar-H), 4.88 (s, 2H, CH_2_), 3.58 (s, 3H, CH_3_), 2.31 (s, 3H, CH_3_); ^13^C NMR (101 MHz, CDCl_3_) *δ* 164.66, 162.18, 160.90, 155.74, 149.76, 147.08, 140.89, 137.33, 132.88, 129.57, 126.02, 120.64, 117.04, 116.79, 116.14, 100.03, 72.92, 34.27, 14.58. HRMS calcd for C_24_H_21_BrFN_4_O_2_S [M + H]^+^ 527.0553, found 527.0558.

*5-(4-Iodophenoxy)-1,3-dimethyl-1H-pyrazole-4-carbaldehyde O-{[6-(4-fluorophenylthio)-pyridin-3-yl]methyl}oxime* (**8e**): yellow oil, yield 53%; ^1^H NMR (400 MHz, CDCl_3_) *δ* 8.34 (d, *J* = 2.30 Hz, 1H, Py-H), 7.73 (s, 1H, CH=N), 7.57 (d, *J* = 2.90 Hz, 2H, Ar-H), 7.56 (d, *J* = 2.40 Hz, 2H, Ar-H), 7.34 (d, *J* = 8.20 Hz, 1H, Py-H), 7.11 (t, *J* = 8.50 Hz, 2H, Ar-H), 6.81 (d, *J* = 8.30 Hz, 1H, Py-H), 6.63 (d, *J* = 8.40 Hz, 2H, Ar-H), 4.87 (s, 2H, CH_2_), 3.57 (s, 3H, CH_3_), 2.31 (s, 3H, CH_3_); ^13^C NMR (101 MHz, CDCl_3_) *δ* 164.65, 162.17, 160.88, 156.60, 149.79, 147.04, 140.87, 138.85, 137.36, 129.56, 126.04, 120.68, 117.51, 117.02, 116.80, 100.06, 86.47, 72.93, 34.27, 14.61. HRMS calcd for C_24_H_21_FIN_4_O_2_S [M + H]^+^ 575.0414, found 575.0417.

*5-(4-Methylphenoxy)-1,3-dimethyl-1H-pyrazole-4-carbaldehyde O-{[6-(4-fluorophenylthio)- pyridin-3-yl]methyl}oxime* (**8f**): yellow oil, yield 52%; ^1^H NMR (400 MHz, CDCl_3_) *δ* 8.34 (d, *J* = 2.30 Hz, 1H, Py-H), 7.74 (s, 1H, CH=N), 7.57–7.54 (m, 2H,Ar-H), 7.38–7.35 (m, 1H, Py-H), 7.11–7.05 (m, 4H, Ar-H), 6.80–6.73 (m, 3H, Py-H and Ar-H), 4.90 (s, 2H, CH_2_), 3.57 (s, 3H, CH_3_), 2.32 (s, 3H, CH_3_), 2.28 (s, 3H, CH_3_); ^13^C NMR (101 MHz, CDCl_3_) *δ* 164.64, 162.15, 160.76, 154.68, 149.81, 148.14, 146.82, 141.35, 137.31, 133.19, 130.40, 129.71, 126.12, 120.63, 116.97, 116.76, 115.08, 99.90, 72.83, 34.20, 20.58, 14.88. HRMS calcd for C_25_H_24_FN_4_O_2_S [M + H]^+^ 463.1604, found 463.1606.

*5-(4-Methoxyphenoxy)-1,3-dimethyl-1H-pyrazole-4-carbaldehyde O-{[6-(4-fluorophenyl- thio) pyridin-3-yl]methyl}oxime* (**8g**): yellow oil, yield 68%; ^1^H NMR (400 MHz, CDCl_3_) *δ* 8.33 (d, *J* = 2.30 Hz, 1H, Py-H), 7.71 (s, 1H, CH=N), 7.56–7.52 (m, 2H, Ar-H), 7.38–7.35 (m, 1H, Py-H), 7.09 (t, *J* = 8.60 Hz, 2H, Ar-H), 6.78 (s, 5H, Py-H and Ar-H), 4.89 (s, 2H, CH_2_), 3.73 (s, 3H, OCH_3_), 3.56 (s, 3H, CH_3_), 2.29 (s, 3H, CH_3_); ^13^C NMR (101 MHz, CDCl_3_) *δ* 164.61, 162.13, 160.80, 155.74, 150.59, 149.84, 148.47, 146.82, 141.34, 137.33, 129.67, 126.05, 120.58, 116.98, 116.76, 116.31, 114.90, 99.64, 72.82, 55.67, 34.20, 14.91. HRMS calcd for C_25_H_24_FN_4_O_3_S [M + H]^+^ 479.1553, found 479.1559.

*5-(4-Trifluoromethoxyphenoxy)-1,3-dimethyl-1H-pyrazole-4-carbaldehyde O-{[6-(4-fluoro-phenylthio) pyridin-3-yl]methyl}oxime* (**8h**): yellow oil, yield 54%; ^1^H NMR (400 MHz, CDCl_3_) *δ* 8.33 (d, *J* = 2.20 Hz, 1H, Py-H), 7.75 (s, 1H, CH=N), 7.57–7.54 (m, 2H, Ar-H), 7.36–7.33 (m, 1H, Py-H), 7.15–7.10 (m, 4H, Ar-H), 6.87 (d, *J* = 9.10 Hz, 2H, Ar-H), 6.79 (d, *J* = 8.20 Hz, 1H, Py-H), 4.86 (s, 2H, CH_2_), 3.59 (s, 3H, CH_3_), 2.31 (s, 3H, CH_3_); ^13^C NMR (101 MHz, CDCl_3_) *δ* 164.66, 162.18, 160.96, 154.91, 149.79, 147.08, 144.74, 140.83, 137.27, 129.49, 125.97, 122.87, 121.69, 120.56, 119.14, 116.98, 116.77, 116.31, 100.03, 72.91, 34.27, 14.56. HRMS calcd for C_25_H_21_F_4_N_4_O_3_S [M + H]^+^ 533.1270, found 533.1274.

*5-(2,4-Difluorophenoxy)-1,3-dimethyl-1H-pyrazole-4-carbaldehyde O-{[6-(4-fluorophenyl-thio) pyridin-3-yl]methyl}oxime* (**8i**): yellow oil, yield 64%; ^1^H NMR (400 MHz, CDCl_3_) *δ* 8.30 (d, *J* = 2.30 Hz, 1H, Py-H), 7.72 (s, 1H, CH=N), 7.56–7.53 (m, 2H, Ar-H), 7.36–7.33 (m, 1H, Py-H), 7.09 (t, *J* = 8.60 Hz, 2H, Ar-H), 6.91–6.86 (m, 1H, Ar-H), 6.77 (d, *J* = 8.30 Hz, 1H, Py-H), 6.71–6.68 (m, 2H, Ar-H), 4.86 (s, 2H, CH_2_), 3.62 (s, 3H, CH_3_), 2.27 (s, 3H, CH_3_); ^13^C NMR (101 MHz, CDCl_3_) *δ* 164.63, 162.14, 160.90, 159.44, 156.99, 153.04, 150.54, 149.64, 147.05, 140.66, 137.39, 137.14, 129.54, 125.96, 120.51, 116.98, 116.76, 111.20, 105.81, 99.43, 72.84, 34.21, 14.37. HRMS calcd for C_24_H_20_F_3_N_4_O_2_S [M + H]^+^ 485.1259, found 485.1262.

*5-(3,4-Dichlorophenoxy)-1,3-dimethyl-1H-pyrazole-4-carbaldehyde O-{[6-(4-fluorophenylthio) pyridin-3-yl]methyl}oxime* (**8j**): yellow oil, yield 61%; ^1^H NMR (400 MHz, CDCl_3_) *δ* 8.32 (d, *J* = 2.20 Hz, 1H, Py-H), 7.75 (s, 1H, CH=N), 7.57–7.54 (m, 2H, Ar-H), 7.33–7.29 (m, 2H, Py-H and Ar-H), 7.10 (t, *J* = 8.60 Hz, 2H, Ar-H), 6.96 (d, *J* = 2.90 Hz, 1H, Ar-H), 6.79 (d, *J* = 8.20 Hz, 1H, Py-H), 6.74–6.71 (m, 1H, Ar-H), 4.87 (s, 2H, CH_2_), 3.58 (s, 3H, CH_3_), 2.31 (s, 3H, CH_3_); ^13^C NMR (101 MHz, CDCl_3_) *δ* 164.65, 162.17, 160.95, 155.32, 149.71, 147.18, 146.44, 140.63, 137.29, 133.59, 131.22, 129.49, 127.30, 126.00, 120.57, 117.43, 116.98, 116.76, 114.93, 100.14, 72.96, 34.31, 14.37. HRMS calcd for C_24_H_20_Cl_2_FN_4_O_2_S [M + H]^+^ 517.0668, found 517.0673.

*5-(4-Fluorophenoxy)-1,3-dimethyl-1H-pyrazole-4-carbaldehyde O-{[6-(4-chlorophenylthio)-pyridin-3-yl]methyl}oxime* (**8k**): yellow oil, yield 57%; ^1^H NMR (400 MHz, CDCl_3_) *δ* 8.33 (d, *J* = 2.20 Hz, 1H, Py-H), 7.73 (s, 1H, CH=N), 7.47 (d, *J* = 8.40 Hz, 2H, Ar-H), 7.38–7.34 (m, 3H, Py-H and Ar-H), 6.97–6.92 (m, 2H, Ar-H), 6.82–6.78 (m, 3H, Py-H and Ar-H), 4.88 (s, 2H, CH_2_), 3.57 (s, 3H, CH_3_), 2.30 (s, 3H, CH_3_); ^13^C NMR (101 MHz, CDCl_3_) *δ* 160.09, 157.49, 152.59, 149.85, 147.69, 146.98, 141.05, 137.33, 136.08, 135.38, 129.83, 129.50, 121.11, 116.62, 116.55, 116.47, 116.39, 99.83, 72.82, 34.24, 14.69. HRMS calcd for C_24_H_21_ClFN_4_O_2_S [M + H]^+^ 483.1058, found 483.1062.

*5-(3-Chlorophenoxy)-1,3-dimethyl-1H-pyrazole-4-carbaldehyde O-{[6-(4-chlorophenylthio)-pyridin-3-yl]methyl}oxime* (**8l**): yellow oil, yield 53%; ^1^H NMR (400 MHz, CDCl_3_) *δ* 8.30 (d, *J* = 2.30 Hz, 1H, Py-H), 7.74 (s, 1H, CH=N), 7.47 (d, *J* = 8.50 Hz, 2H, Ar-H), 7.36–7.32 (m, 3H, Py-H and Ar-H), 7.18 (t, *J* = 8.20 Hz, 1H, Ar-H), 7.02 (d, *J* = 8.80 Hz, 1H, Ar-H), 6.85–6.82 (m, 3H, Py-H and Ar-H), 4.87 (s, 2H, CH_2_), 3.56 (s, 3H, CH_3_), 2.30 (s, 3H, CH_3_); ^13^C NMR (101 MHz, CDCl_3_) *δ* 160.03, 157.07, 149.81, 147.03, 146.78, 140.85, 137.29, 136.07, 135.39, 130.81, 129.81, 123.95, 121.12, 115.84, 113.53, 100.14, 72.85, 34.29, 14.55. HRMS calcd for C_24_H_21_Cl_2_N_4_O_2_S [M + H]^+^ 499.0762, found 499.0766.

*5-(4-Chlorophenoxy)-1,3-dimethyl-1H-pyrazole-4-carbaldehyde O-{[6-(4-chlorophenylthio)-pyridin-3-yl]methyl}oxime* (**8m**): yellow oil, yield 48%; ^1^H NMR (400 MHz, CDCl_3_) *δ* 8.32 (d, *J* = 2.30 Hz, 1H, Py-H), 7.73 (s, 1H, CH=N), 7.46 (d, *J* = 8.50 Hz, 2H, Ar-H), 7.35–7.31 (m, 3H, Py-H and Ar-H), 7.20 (d, *J* = 9.00 Hz, 2H, Ar-H), 6.84 (d, *J* = 8.30 Hz, 1H, Py-H), 6.77 (d, *J* = 9.00 Hz, 2H, Ar-H), 4.86 (s, 2H, CH_2_), 3.55 (s, 3H, CH_3_), 2.29 (s, 3H, CH_3_); ^13^C NMR (101 MHz, CDCl_3_) *δ* 160.08, 155.16, 149.87, 147.13, 146.99, 140.91, 137.33, 136.06, 135.34, 129.92, 129.86, 129.81, 129.54, 128.71, 121.13, 116.58, 100.00, 72.86, 34.26, 14.61. HRMS calcd for C_24_H_21_Cl_2_N_4_O_2_S [M + H]^+^ 499.0762, found 499.0768.

*5-(4-Bromophenoxy)-1,3-dimethyl-1H-pyrazole-4-carbaldehyde O-{[6-(4-chlorophenylthio)-pyridin-3-yl]methyl}oxime* (**8n**): yellow oil, yield 48%; ^1^H NMR (400 MHz, CDCl_3_) *δ* 8.31 (d, *J* = 2.30 Hz, 1H, Py-H), 7.72 (s, 1H, CH=N), 7.45 (d, *J* = 8.20 Hz, 2H, Ar-H), 7.35–7.31 (m, 5H, Py-H and Ar-H), 6.85 (d, *J* = 8.20 Hz, 1H, Py-H), 6.72 (d, *J* = 8.50 Hz, 2H, Ar-H), 4.86 (s, 2H, CH_2_), 3.55 (s, 3H, CH_3_), 2.29 (s, 3H, CH_3_); ^13^C NMR (101 MHz, CDCl_3_) *δ* 160.02, 155.72, 149.89, 147.03, 146.97, 140.88, 137.29, 136.02, 135.30, 132.87, 129.87, 129.79, 129.60, 121.17, 117.05, 116.13, 100.02, 72.87, 34.27, 14.60. HRMS calcd for C_24_H_21_BrClN_4_O_2_S [M + H]^+^ 543.0257, found 543.0256.

*5-(4-Methylphenoxy)-1,3-dimethyl-1H-pyrazole-4-carbaldehyde O-{[6-(4-chlorophenylthio)pyridin-3-yl]methyl}oxime* (**8o**): yellow oil, yield 73%; ^1^H NMR (400 MHz, CDCl_3_) *δ* 8.35 (d, *J* = 2.30 Hz, 1H, Py-H), 7.74 (s, 1H, CH=N), 7.48 (d, *J* = 8.10 Hz, 2H, Ar-H), 7.36 (t, *J* = 8.20 Hz, 3H, Py-H and Ar-H), 7.06 (d, *J* = 8.10 Hz, 2H, Ar-H), 6.86 (d, *J* = 8.20 Hz, 1H, Py-H), 6.74 (d, *J* = 8.20 Hz, 2H, Ar-H), 4.91 (s, 2H, CH_2_), 3.57 (s, 3H, CH_3_), 2.32 (s, 3H, CH_3_), 2.28 (s, 3H, CH_3_); ^13^C NMR (101 MHz, CDCl_3_) *δ* 159.96, 154.67, 149.87, 148.13, 146.82, 141.39, 137.45, 136.00, 135.35, 133.20, 130.41, 130.04, 129.82, 129.63, 121.22, 115.07, 99.90, 72.80, 34.22, 20.60, 14.90. HRMS calcd for C_25_H_24_ClN_4_O_2_S [M + H]^+^ 479.1308, found 479.1313.

*5-(4-Methoxyphenoxy)-1,3-dimethyl-1H-pyrazole-4-carbaldehyde O-{[6-(4-chlorophenyl-thio)pyridin-3-yl]methyl}oxime* (**8p**): yellow oil, yield 47%; ^1^H NMR (400 MHz, CDCl_3_) *δ* 8.33 (d, *J* = 2.30 Hz, 1H, Py-H), 7.71 (s, 1H, CH=N), 7.46 (d, *J* = 8.50 Hz, 2H, Ar-H), 7.39–7.32 (m, 3H, Py-H and Ar-H), 6.84 (d, *J* = 8.20 Hz, 1H, Py-H), 6.77 (s, 4H, Ar-H), 4.89 (s, 2H, CH_2_), 3.72 (s, 3H, CH_3_), 3.55 (s, 3H, OCH_3_), 2.30 (s, 3H, CH_3_); ^13^C NMR (101 MHz, CDCl_3_) *δ* 159.96, 155.73, 150.58, 149.92, 148.46, 146.79, 141.36, 137.40, 136.00, 135.29, 130.00, 129.80, 129.61, 121.16, 116.31, 114.89, 99.62, 72.78, 55.66, 34.21, 14.92. HRMS calcd for C_25_H_24_ClN_4_O_3_S [M + H]^+^ 495.1258, found 495.1262.

*5-(2,4-Difluorophenoxy)-1,3-dimethyl-1H-pyrazole-4-carbaldehyde O-{[6-(4-chlorophenyl-thio)pyridin-3-yl]methyl}oxime* (**8q**): yellow oil, yield 61%; ^1^H NMR (400 MHz, CDCl_3_) *δ* 8.32 (d, *J* = 2.20 Hz, 1H, Py-H), 7.73 (s, 1H, CH=N), 7.49 (d, *J* = 8.40 Hz, 2H, Ar-H), 7.36 (d, *J* = 8.40 Hz, 3H, Py-H and Ar-H), 6.92–6.84 (m, 2H, Py-H and Ar-H), 6.71–6.69 (m, 2H, Ar-H), 4.87 (s, 2H, CH_2_), 3.63 (s, 3H, CH_3_), 2.28 (s, 3H, CH_3_); ^13^C NMR (101 MHz, CDCl_3_) *δ* 160.14, 159.46, 157.02, 153.05, 150.55, 149.76, 147.08, 140.71, 137.19, 136.09, 135.39, 129.83, 129.51, 121.10, 117.34, 111.17, 110.98, 105.84, 105.36, 99.43, 72.84, 34.25, 14.42. HRMS calcd for C_24_H_20_ClF_2_N_4_O_2_S [M + H]^+^ 501.0964, found 501.0968.

*5-(2,4-Dimethylphenoxy)-1,3-dimethyl-1H-pyrazole-4-carbaldehyde O-{[6-(4-chlorophenyl-thio)pyridin-3-yl]methyl}oxime* (**8r**): yellow oil, yield 48%; ^1^H NMR (400 MHz, CDCl_3_) *δ* 8.34 (d, *J* = 2.20 Hz, 1H, Py-H), 7.67 (s, 1H, CH=N), 7.47 (d, *J* = 8.50 Hz, 2H, Ar-H), 7.36–7.34 (m, 3H, Py-H and Ar-H), 6.99 (s, 1H, Ar-H), 6.85–6.81 (m, 2H, Py-H and Ar-H), 6.38 (d, *J* = 8.30 Hz, 1H, Ar-H), 4.89 (s, 2H, CH_2_), 3.56 (s, 3H, CH_3_), 2.32 (s, 3H, CH_3_), 2.30 (s, 3H, CH_3_), 2.23 (s, 3H, CH_3_); ^13^C NMR (101 MHz, CDCl_3_) *δ* 159.96, 152.87, 149.87, 148.54, 146.82, 141.40, 137.39, 136.02, 135.33, 133.05, 132.22, 130.05, 129.81, 129.63, 127.51, 126.19, 121.18, 113.33, 99.51, 72.76, 34.11, 20.58, 16.04, 14.93. HRMS calcd for C_26_H_26_ClN_4_O_2_S [M + H]^+^ 493.1465, found 493.1470.

*5-(4-Fluorophenoxy)-1,3-dimethyl-1H-pyrazole-4-carbaldehyde O-{[6-(phenylthio)pyridin-3-yl]methyl}oxime* (**8s**): yellow oil, yield 46%; ^1^H NMR (400 MHz, CDCl_3_) *δ* 8.33 (d, *J* = 2.20 Hz, 1H, Py-H), 7.72 (s, 1H, CH=N), 7.56–7.54 (m, 2H, Ar-H), 7.39–7.35 (m, 4H, Py-H and Ar-H), 6.96–6.92 (m, 2H, Ar-H), 6.81–6.77 (m, 3H, Py-H and Ar-H), 4.87 (s, 2H, CH_2_), 3.56 (s, 3H, CH_3_), 2.30 (s, 3H, CH_3_); ^13^C NMR (101 MHz, CDCl_3_) *δ* 161.16, 159.90, 157.49, 152.59, 149.75, 147.69, 146.98, 141.00, 137.28, 134.99, 130.90, 129.69, 129.47, 129.20, 120.88, 116.63, 116.39, 99.87, 72.90, 34.23, 14.69. HRMS calcd for C_24_H_22_FN_4_O_2_S [M + H]^+^ 449.1447, found 449.1452.

*5-(3-Chlorophenoxy)-1,3-dimethyl-1H-pyrazole-4-carbaldehyde O-{[6-(phenylthio)pyridin-3-yl]methyl}oxime* (**8t**): yellow oil, yield 53%; ^1^H NMR (400 MHz, CDCl_3_) *δ* 8.32 (d, *J* = 2.30 Hz, 1H, Py-H), 7.75 (s, 1H, CH=N), 7.58–7.55 (m, 2H, Ar-H), 7.40 (t, *J* = 2.80 Hz, 3H, Py-H and Ar-H), 7.32–7.29 (m, 1H, Ar-H), 7.18 (t, *J* = 8.20 Hz, 1H, Ar-H), 7.02 (d, *J* = 8.90 Hz, 1H, Ar-H), 6.85 (t, *J* = 2.20 Hz, 1H, Py-H), 6.81–6.69 (m, 2H, Ar-H), 4.87 (s, 2H, CH_2_), 3.57 (s, 3H, CH_3_), 2.31 (s, 3H, CH_3_); ^13^C NMR (101 MHz, CDCl_3_) *δ* 161.14, 157.07, 149.69, 147.07, 146.79, 140.82, 137.24, 135.40, 135.01, 130.94, 130.82, 129.69, 129.45, 129.19, 123.96, 120.89, 115.83, 113.52, 100.17, 72.93, 34.29, 14.54. HRMS calcd for C_24_H_22_ClN_4_O_2_S [M + H]^+^ 465.1152, found 465.1155.

*5-(4-Chlorophenoxy)-1,3-dimethyl-1H-pyrazole-4-carbaldehyde O-{[6-(phenylthio)pyridin-3-yl]methyl}oxime* (**8u**): yellow oil, yield 43%; ^1^H NMR (400 MHz, CDCl_3_) *δ* 8.31 (d, *J* = 2.30 Hz, 1H, Py-H), 7.74 (s, 1H, CH=N), 7.56–7.54 (m, 2H, Ar-H), 7.39–7.38 (m, 3H, Py-H and Ar-H), 7.31–7.28 (m, 1H, Ar-H), 7.17 (t, *J* = 8.20 Hz, 1H, Ar-H), 7.01 (d, *J* = 8.00 Hz, 1H, Ar-H), 6.84 (s, 1H, Py-H), 6.81–6.64 (m, 2H, Ar-H), 4.86 (s, 2H, CH_2_), 3.56 (s, 3H, CH_3_), 2.31 (s, 3H, CH_3_); ^13^C NMR (101 MHz, CDCl_3_) *δ* 161.11, 157.07, 149.67, 147.04, 146.77, 140.81, 137.24, 135.39, 135.00, 130.92, 130.82, 129.69, 129.46, 129.19, 123.95, 120.88, 115.83, 113.52, 100.16, 72.91, 34.29, 14.55. HRMS calcd for C_24_H_22_ClN_4_O_2_S [M + H]^+^ 465.1152, found 465.1154.

*5-(4-Bromophenoxy)-1,3-dimethyl-1H-pyrazole-4-carbaldehyde O-{[6-(phenylthio)pyridin-3-yl]methyl}oxime* (**8v**): yellow oil, yield 51%; ^1^H NMR (400 MHz, CDCl_3_) *δ* 8.35 (d, *J* = 2.30 Hz, 1H, Py-H), 7.74 (s, 1H, CH=N), 7.57 (d, *J* = 6.20 Hz, 2H, Ar-H), 7.40–7.30 (m, 6H, Py-H and Ar-H), 6.82 (d, *J* = 8.30 Hz, 1H, Py-H), 6.74 (d, *J* = 8.40 Hz, 2H, Ar-H), 4.87 (s, 2H, CH_2_), 3.57 (s, 3H, CH_3_), 2.31 (s, 3H, CH_3_); ^13^C NMR (101 MHz, CDCl_3_) *δ* 161.15, 155.74, 149.75, 147.08, 147.04, 140.86, 137.30, 134.96, 132.89, 130.98, 129.69, 129.46, 129.17, 120.97, 117.05, 116.15, 100.05, 72.96, 34.27, 14.61. HRMS calcd for C_24_H_22_BrN_4_O_2_S [M + H]^+^ 509.0647, found 509.0650.

*5-(4-Iodophenoxy)-1,3-dimethyl-1H-pyrazole-4-carbaldehyde O-{[6-(phenylthio)pyridin-3-yl]methyl}oxime* (**8w**): yellow oil, yield 64%; ^1^H NMR (400 MHz, CDCl_3_) *δ* 8.36 (d, *J* = 2.30 Hz, 1H, Py-H), 7.74 (s, 1H, CH=N), 7.59–7.56 (m, 4H, Ar-H), 7.42–7.40 (m, 3H, Py-H and Ar-H), 7.34–7.31 (m, 1H, Ar-H), 6.84 (d, *J* = 8.20 Hz, 1H, Py-H), 6.63 (d, *J* = 8.80 Hz, 2H, Ar-H), 4.88 (s, 2H, CH_2_), 3.57 (s, 3H, CH_3_), 2.31 (s, 3H, CH_3_); ^13^C NMR (101 MHz, CDCl_3_) *δ* 161.15, 156.60, 149.79, 147.05, 147.00, 140.85, 138.85, 137.31, 134.96, 131.01, 129.70, 129.45, 129.17, 121.02, 117.51, 100.08, 86.47, 72.99, 34.28, 14.62. HRMS calcd for C_24_H_22_IN_4_O_2_S [M + H]^+^ 557.0508, found 557.0511.

*5-(4-Methylphenoxy)-1,3-dimethyl-1H-pyrazole-4-carbaldehyde O-{[6-(phenylthio)pyridin-3-yl]methyl}oxime* (**8x**): yellow oil, yield 55%; ^1^H NMR (400 MHz, CDCl_3_) *δ* 8.35 (d, *J* = 2.20 Hz, 1H, Py-H), 7.74 (s, 1H, CH=N), 7.58–7.55 (m, 2H, Ar-H), 7.40–7.33 (m, 4H, Py-H and Ar-H), 7.06 (d, *J* = 8.30 Hz, 2H, Ar-H), 6.86–6.67 (m, 3H, Py-H and Ar-H), 4.90 (s, 2H, CH_2_), 3.56 (s, 3H, CH_3_), 2.32 (s, 3H, CH_3_), 2.27 (s, 3H, CH_3_); ^13^C NMR (101 MHz, CDCl_3_) *δ* 161.03, 154.68, 149.80, 148.12, 146.82, 141.32, 137.33, 134.91, 133.19, 131.09, 130.41, 129.66, 129.61, 129.12, 120.96, 115.08, 99.92, 72.88, 34.21, 20.60, 14.88. HRMS calcd for C_25_H_25_N_4_O_2_S [M + H]^+^ 445.1698, found 445.1702.

*5-(4-Methoxyphenoxy)-1,3-dimethyl-1H-pyrazole-4-carbaldehyde O-{[6-(phenylthio)- pyridin-3-yl]methyl}oxime* (**8y**): yellow oil, yield 45%; ^1^H NMR (400 MHz, CDCl_3_) *δ* 8.35 (s, 1H, Py-H), 7.72 (s, 1H, CH=N), 7.57–7.55 (m, 2H, Ar-H), 7.40–7.35 (m, 4H, Py-H and Ar-H), 6.80 (s, 5H, Py-H and Ar-H), 4.90 (s, 2H, CH_2_), 3.74 (s, 3H, CH_3_), 3.57 (s, 3H, OCH_3_), 2.31 (s, 3H, CH_3_); ^13^C NMR (101 MHz, CDCl_3_) *δ* 161.05, 155.76, 150.61, 149.80, 148.49, 146.85, 141.33, 137.33, 134.91, 131.04, 129.66, 129.60, 129.13, 120.97, 116.32, 114.91, 99.66, 72.88, 55.69, 14.89. HRMS calcd for C_25_H_25_N_4_O_3_S [M + H]^+^ 461.1647, found 461.1652.

*5-(2,4-Dimethylphenoxy)-1,3-dimethyl-1H-pyrazole-4-carbaldehyde O-{[6-(phenylthio)- pyridin-3-yl]methyl}oxime* (**8z**): yellow oil, yield 49%; ^1^H NMR (400 MHz, CDCl_3_) *δ* 8.35 (d, *J* = 2.20 Hz, 1H, Py-H), 7.67 (s, 1H, CH=N), 7.59–7.56 (m, 2H, Ar-H), 7.42–7.40 (m, 3H, Py-H and Ar-H), 7.34–7.32 (m, 1H, Ar-H), 7.00 (s, 1H, Ar-H), 6.84–6.79 (m, 2H, Py-H and Ar-H), 6.38 (d, *J* = 8.30 Hz, 1H, Ar-H), 4.90 (s, 2H, CH_2_), 3.57 (s, 3H, CH_3_), 2.31 (d, *J* = 7.50 Hz, 6H, 2 × CH_3_), 2.24 (s, 3H, CH_3_); ^13^C NMR (101 MHz, CDCl_3_) *δ* 161.07, 152.87, 149.73, 148.54, 146.87, 141.38, 137.36, 134.96, 133.05, 132.21, 131.00, 129.70, 129.62, 129.18, 127.49, 126.20, 120.94, 113.31, 99.52, 72.84, 34.11, 20.57, 16.03, 14.92. HRMS calcd for C_26_H_27_N_4_O_2_S [M + H]^+^ 459.1855, found 459.1858.

#### 3.1.5. General Approach to Preparation of **9a**,**b**

To a mixture of potassium hydroxide (36 mmol, 2.02 g), water (2 mL) and DMF (50 mL), a solution of substituted thiophenol (30 mmol) in DMF (10 mL) was added dropwise at room temperature, and the mixture was stirred at 55 °C for 2–3 h. Then, compound **1** (20 mmol, 3.17 g) was added slowly thereto, and the reaction mixture was heated to 100 °C for 6–8 h. The reaction system was cooled and poured into ice water (150 mL). The resulting mixture was extracted with ethyl acetate (3 × 60 mL). The combined ethyl acetate layer was dried on anhydrous magnesium sulfate, filtered, and removed in vacuum to form intermediates **9a**,**b**, which were directly used in the following step.

#### 3.1.6. General Approach to Preparation of **10a**,**b**

To a mixture of hydroxylamine hydrochloride (15 mmol) and CH_3_OH (40 mL), potassium hydroxide (20 mmol, 1.12 g) was added in portions, and the resulting solution was stirred at room temperature for 10 min. Next, compound **9** (10 mmol) was added slowly thereto, and the reaction system was refluxed for 3 h. After cooling, the mixture was dumped into ice water (100 mL), and the resulting solid was filtered to give intermediates **10a**,**b**.

*5-(4-Fluorophenylthio)-1,3-dimethyl-1H-pyrazole-4-carbaldehyde oxime* (**10a**): colorless solid, yield 62%, m.p. 182–184 °C; ^1^H NMR (400 MHz, CDCl_3_) *δ* 8.22 (s, 1H, OH), 7.97 (s, 1H, CH=N), 7.09–6.91 (m, 4H, Ar-H), 3.80 (s, 3H, CH_3_), 2.44 (s, 3H, CH_3_); ^13^C NMR (101 MHz, CDCl_3_) *δ* 163.06, 160.60, 147.44, 144.15, 131.94, 129.45, 129.37, 129.25, 117.33, 116.62, 36.62, 14.48. HRMS calcd for C_12_H_13_FN_3_OS [M + H]^+^ 266.0763, found 266.0769.

*5-(4-Chlorophenylthio)-1,3-dimethyl-1H-pyrazole-4-carbaldehyde oxime* (**10b**): colorless solid, yield 65%, m.p. 210–212 °C; ^1^H NMR (400 MHz, CDCl_3_) *δ* 8.18 (s, 1H, OH), 7.52 (s, 1H, CH=N), 7.23 (d, *J* = 8.50 Hz, 2H, Ar-H), 6.93 (d, *J* = 8.60 Hz, 2H, Ar-H), 3.79 (s, 3H, CH_3_), 2.44 (s, 3H, CH_3_); ^13^C NMR (101 MHz, CDCl_3_) *δ* 147.60, 144.19, 131.89, 129.99, 129.66, 128.11, 117.57, 36.63, 14.51. HRMS calcd for C_12_H_13_ClN_3_OS, [M + H]^+^ 282.0468, found 282.0472.

#### 3.1.7. General Approach to Preparation of Target Compounds **11a**–**f**

To a mixture of potassium carbonate (20 mmol, 2.76 g), compound **10** (11 mmol) in DMF (30 mL), compound **7** (10 mmol) was added, and the generating solution was heated to 55 °C for 5–6 h. Subsequently, 30 mL of water was added slowly thereto. The formative mixture was extracted through dichloromethane (3 × 20 mL). The collected extracts were then dried on anhydrous sodium sulfate, filtered, and removed under reduced pressure to obtain the crude product, which was purified through silica gel column chromatography eluting with ethyl acetate/petroleum ether (1:4, *v*/*v*) to obtain the designed compounds **11a**-**f** smoothly, and the spectral data of compounds **11a**-**f** are depicted below. ^1^H NMR and ^13^CNMR spectra are listed in Supplementary Materials.

*5-(4-Fluorophenthio)-1,3-dimethyl-1H-pyrazole-4-carbaldehyde O-{[6-(4-fluorophenylthio)pyridin-3-yl]methyl}oxime* (**11a**): yellow oil, yield 64%; ^1^H NMR (400 MHz, CDCl_3_) *δ* 8.44 (d, *J* = 2.30 Hz, 1H, Py-H), 8.14 (s, 1H, CH=N), 7.59–7.56 (m, 2H, Ar-H), 7.52–7.49 (m, 1H, Py-H), 7.12 (t, *J* = 8.60 Hz, 2H, Ar-H), 7.00–6.92 (m, 4H, Ar-H), 6.84 (d, *J* = 8.20 Hz, 1H, Py-H), 5.04 (s, 2H, CH_2_), 3.76 (s, 3H, CH_3_), 2.39 (s, 3H, CH_3_); ^13^C NMR (101 MHz, CDCl_3_) *δ* 164.68, 163.00, 162.19, 161.04, 160.54, 149.93, 147.60, 143.39, 137.37, 131.85, 129.57, 129.26, 126.00, 120.64, 117.19, 117.02, 116.80, 116.59, 73.10, 36.63, 14.70. HRMS calcd for C_24_H_21_F_2_N_4_OS_2_ [M + H]^+^ 483.1125, found 483.1129.

*5-(4-Chlorophenthio)-1,3-dimethyl-1H-pyrazole-4-carbaldehyde O-{[6-(4-fluorophenylthio)pyridin-3-yl]methyl}oxime* (**11b**): yellow oil, yield 59%; ^1^H NMR (400 MHz, CDCl_3_) *δ* 8.43 (s, 1H, Py-H), 8.10 (s, 1H, CH=N), 7.58–7.54 (m, 2H, Ar-H), 7.50–7.48 (m, 1H, Py-H), 7.19 (d, *J* = 8.40 Hz, 2H, Ar-H), 7.11 (t, *J* = 8.50 Hz, 2H, Ar-H), 6.88 (d, *J* = 8.30 Hz, 2H, Ar-H), 6.83 (d, *J* = 8.20 Hz, 1H, Py-H), 5.03 (s, 2H, CH_2_), 3.76 (s, 3H, CH_3_), 2.40 (s, 3H, CH_3_); ^13^C NMR (101 MHz, CDCl_3_) *δ* 164.65, 162.17, 161.02, 149.96, 147.68, 143.22, 137.35, 133.03, 132.64, 130.89, 129.62, 128.07, 126.03, 120.64, 117.44, 117.00, 116.79, 73.12, 36.63, 14.70. HRMS calcd for C_24_H_21_ClFN_4_OS_2_ [M + H]^+^ 499.0829, found 499.0833.

*5-(4-Fluorophenthio)-1,3-dimethyl-1H-pyrazole-4-carbaldehyde O-{[6-(4-chlorophenylthio)pyridin-3-yl]methyl}oxime* (**11c**): yellow oil, yield 42%; ^1^H NMR (400 MHz, CDCl_3_) *δ* 8.45 (d, *J* = 2.30 Hz, 1H, Py-H), 8.14 (s, 1H, CH=N), 7.53–7.48 (m, 3H, Py-H and Ar-H), 7.37 (d, *J* = 8.50 Hz, 2H, Ar-H), 7.00–6.90 (m, 5H, Py-H and Ar-H), 5.04 (s, 2H, CH_2_), 3.76 (s, 3H, CH_3_), 2.39 (s, 3H, CH_3_); ^13^C NMR (101 MHz, CDCl_3_) *δ* 163.00, 160.54, 160.23, 150.04, 147.59, 143.40, 137.46, 136.05, 135.40, 131.86, 129.89, 129.84, 129.55, 129.35, 129.27, 121.21, 117.17, 116.81, 116.59, 73.08, 36.63, 14.70. HRMS calcd for C_24_H_21_ClFN_4_OS_2_ [M + H]^+^ 499.0829, found 499.0834.

*5-(4-Chlorophenthio)-1,3-dimethyl-1H-pyrazole-4-carbaldehyde O-{[6-(4-chlorophenylthio)pyridin-3-yl]methyl}oxime* (**11d**): yellow oil, yield 55%; ^1^H NMR (400 MHz, CDCl_3_) *δ* 8.44 (d, *J* = 2.30 Hz, 1H, Py-H), 8.10 (s, 1H, CH=N), 7.52–7.48 (m, 3H, Py-H and Ar-H), 7.36 (d, *J* = 8.50 Hz, 2H, Ar-H), 7.19 (d, *J* = 8.60 Hz, 2H, Ar-H), 6.89 (t, *J* = 8.80 Hz, 3H, Py-H and Ar-H), 5.04 (s, 2H, CH_2_), 3.76 (s, 3H, CH_3_), 2.40 (s, 3H, CH_3_); ^13^C NMR (101 MHz, CDCl_3_) *δ* 160.23, 150.04, 147.68, 143.25, 137.45, 136.04, 135.39, 133.02, 132.65, 131.08, 130.90, 129.97, 129.85, 129.84, 129.63, 129.56, 128.07, 121.21, 117.43, 73.10, 36.63, 14.71. HRMS calcd for C_24_H_21_Cl_2_N_4_OS_2_ [M + H]^+^ 515.0534, found 515.0536.

*5-(4-Fluorophenthio)-1,3-dimethyl-1H-pyrazole-4-carbaldehyde O-{[6-(phenylthio)pyridin-3-yl]methyl}oxime* (**11e**): yellow oil, yield 65%; ^1^H NMR (400 MHz, CDCl_3_) *δ* 8.45 (d, *J* = 2.30 Hz, 1H, Py-H), 8.14 (s, 1H, CH=N), 7.58–7.56 (m, 2H, Ar-H), 7.50–7.47 (m, 1H, Py-H), 7.41–7.39 (m, 3H, Ar-H), 7.00–6.91 (m, 4H, Ar-H), 6.85 (d, *J* = 8.30 Hz, 1H, Py-H), 5.04 (s, 2H, CH_2_), 3.76 (s, 3H, CH_3_), 2.39 (s, 3H, CH_3_); ^13^C NMR (101 MHz, CDCl_3_) *δ* 162.99, 161.29, 160.53, 149.93, 147.59, 143.35, 137.40, 134.96, 131.83, 130.96, 129.69, 129.47, 129.35, 129.27, 129.18, 120.96, 117.21, 116.80, 116.58, 73.15, 36.62, 14.71. HRMS calcd for C_24_H_22_FN_4_OS_2_ [M + H]^+^ 465.1219, found 465.1221.

*5-(4-Chlorophenthio)-1,3-dimethyl-1H-pyrazole-4-carbaldehyde O-{[6-(phenylthio)pyridin-3-yl]methyl}oxime* (**11f**): yellow oil, yield 74%; ^1^H NMR (400 MHz, CDCl_3_) *δ* 8.45 (d, *J* = 2.30 Hz, 1H, Py-H), 8.10 (s, 1H, CH=N), 7.58–7.55 (m, 2H, Ar-H), 7.49–7.46 (m 1H, Py-H), 7.40–7.39 (m, 3H, Ar-H), 7.18 (d, *J* = 8.60 Hz, 2H, Ar-H), 6.89–6.84 (m, 3H, Py-H and Ar-H), 5.03 (s, 2H, CH_2_), 3.75 (s, 3H, CH_3_), 2.40 (s, 3H, CH_3_); ^13^C NMR (101 MHz, CDCl_3_) *δ* 161.28, 149.90, 147.68, 143.20, 137.41, 134.96, 133.04, 132.63, 130.95, 130.88, 129.69, 129.63, 129.45, 129.19, 128.08, 120.98, 117.46, 73.16, 36.63, 14.72. HRMS calcd for C_24_H_22_ClN_4_OS_2_ [M + H]^+^ 481.0924, found 481.0928.

### 3.2. Biological Assay

#### 3.2.1. Insecticidal Activities against *Mythimna separata* and *Plutella xylostella*

The larvicidal properties of compounds **8a**–**z** and **11a**–**f** against *Mythimna separata* and *Plutella xylostella* were determined by a leaf-dipping method [31]. Firstly, the tested sample was dissolved in the solvent of DMF including 1% Tween-80 emulsifier, and diluted to the demanded dosages through water. Some corn leaves were fully immersed in the generated solutions for 3 s and then air-dried. Subsequently, the treated leaves were released into a culture plate containing filter paper, into which third-instar *M. separata* and second-instar *P. xylostella* larvae were inoculated. The above culture plate was put into a room for normal cultivation at 25 °C. Mortality was evaluated 48 h after treatment. The larvae who had no response to the touch of a writing brush were considered dead. Each assay was performed three times. Tolfenpyrad and Pyridalyl, as the controls, were studied under equal conditions.

#### 3.2.2. Insecticidal Activities against *Tetranychus cinnabarinus*, *Aphis medicaginis* and *Nilaparvata lugens*

The insecticidal activities of compounds **8a**–**z** and **11a**–**f** towards *Tetranychus cinnabarinus*, *Aphis medicaginis*, and *Nilaparvata lugens* were researched with a spray approach [32]. Through the use of a Potter spray tower, broad bean leaves owning *T. cinnabarinus* or *A. medicaginis*, and oryza sativa seedlings possessing *N. lugens*, respectively, were sprayed with solutions of the examined compounds. After that, the above broad bean leaves or oryza sativa seedlings were placed in a room for general cultivation at 25 °C. Mortality was counted 48 h after spray treatment. Each experiment was executed three times. Fenpyroximate, Imidacloprid, and Abamectin were separately chosen as corresponding controls and investigated under similar conditions.

## 4. Conclusions

In conclusion, a series of novel pyridyl-containing pyrazole oxime ethers were synthesized and assessed for the insecticidal activities against five crop pests. The results displayed that the majority of the designed compounds exhibited favorable insecticidal properties towards *M. separata*, *T. cinnabarinus*, *P. xylostella*, and *A. medicaginis* at a dosage of 500 μg/mL. Some of the desired compounds demonstrated moderate to good insecticidal effects against *M. separata* and *T. cinnabarinus* when the dosage was reduced to 100 μg/mL, and some derivatives expressed satisfactory inhibitory activities against *A. medicaginis* at dosages of 100 μg/mL and 20 μg/mL, compounds **8a**, **8c**–**f**, **8o**, **8v**, **8x**, and **8z**, especially, still had 50% to 70% of their insecticidal activity against *A. medicaginis* at 20 μg/mL. In addition, a few derivatives also possessed wonderful insecticidal properties towards *N. lugens* at 500 μg/mL. Interestingly, compounds **8a**, **8c**, **8d**, **8s**, **8v**, **8x**, and **8z** indicated broad-spectrum insecticidal properties towards all five pests, and they could be selected as promising insecticidal lead compounds for deeper structural modification and biological activity studies.

## Figures and Tables

**Table 1:** Insecticidal activities of title compounds **8a**–**z** and **11a**–**f** (mortality, %).

Compd.	*Mythimna separata*			*Tetranychus cinnabarinus*			*Plutella xylostella*	
	500 μg/mL	100 μg/mL	20 μg/mL	500 μg/mL	100 μg/mL	20 μg/mL	500 μg/mL	100 μg/mL
**8a**	100	100	30	100	100	40	100	0
**8b**	100	70	0	100	0	— ^a^	100	30
**8c**	100	20	—	100	100	0	100	0
**8d**	100	100	0	100	0	—	100	0
**8e**	100	20	—	100	0	—	100	0
**8f**	100	30	—	100	30	—	80	0
**8g**	70	0	—	100	60	0	100	0
**8h**	100	20	—	0	—	—	100	0
**8i**	40	0	—	100	100	0	100	30
**8j**	100	0	—	100	30	—	50	0
**8k**	60	0	—	100	100	40	100	30
**8l**	100	30	—	70	0	—	100	0
**8m**	50	0	—	100	80	0	70	0
**8n**	100	60	10	80	0	—	60	0
**8o**	50	0	—	100	70	0	100	0
**8p**	40	0	—	100	0	—	100	0
**8q**	70	0	—	60	0	—	100	0
**8r**	0	—	—	100	0	—	80	0
**8s**	100	100	50	100	50	0	100	20
**8t**	100	60	30	60	0	—	50	0
**8u**	100	70	20	50	0	—	80	0
**8v**	100	100	30	100	40	0	80	0
**8w**	100	50	20	80	0	—	80	0
**8x**	100	60	20	100	70	0	100	0
**8y**	100	20	—	70	0	—	90	0
**8z**	100	50	20	100	50	0	100	0
**11a**	20	—	—	20	—	—	80	0
**11b**	40	0	—	50	0	—	80	0
**11c**	10	—	—	60	0	—	100	0
**11d**	80	0	—	0	—	—	30	0
**11e**	70	0	—	60	0	—	0	—
**11f**	0	—	—	20	—	—	20	—
Tolfenpyrad	100	50	40	—	—	—	—	—
Fenpyroximate	—	—	—	100	100	100	—	—
Pyridalyl	—	—	—	—	—	—	100	100

^a^ “—” refers to “not tested”.

**Table 2:** Insecticidal activities of target compounds **8a**–**z** and **11a**–**f** (mortality, %).

Compd.	*Aphis medicaginis*			*Nilaparvata lugens*		
	500 μg/mL	100 μg/mL	20 μg/mL	500 μg/mL	100 μg/mL	20 μg/mL
**8a**	100	100	60	100	30	— ^a^
**8b**	100	40	0	0	—	—
**8c**	100	100	50	100	0	—
**8d**	100	100	50	100	0	—
**8e**	100	100	60	100	20	—
**8f**	100	100	70	100	0	—
**8g**	100	100	0	100	20	—
**8h**	100	30	—	100	0	—
**8i**	100	60	0	80	0	—
**8j**	90	0	—	0	—	—
**8k**	100	70	0	60	0	—
**8l**	100	80	0	0	—	—
**8m**	100	0	—	50	0	—
**8n**	100	0	—	0	—	—
**8o**	100	100	60	0	—	—
**8p**	100	90	0	50	0	—
**8q**	100	70	0	0	—	—
**8r**	100	70	0	20	—	—
**8s**	100	100	40	100	30	—
**8t**	80	0	—	70	0	—
**8u**	100	30	—	100	0	—
**8v**	100	100	60	50	0	—
**8w**	100	80	0	90	0	—
**8x**	100	100	60	100	0	—
**8y**	100	0	—	60	0	—
**8z**	100	100	50	80	0	—
**11a**	60	0	—	0	—	—
**11b**	50	0	—	0	—	—
**11c**	20	—	—	0	—	—
**11d**	50	0	—	0	—	—
**11e**	70	0	—	0	—	—
**11f**	40	0	—	0	—	—
Imidacloprid	100	100	100	—	—	—
Abamectin	—	—	—	100	100	100

^a^ “—” refers to “not tested”.

## Data Availability

Data are contained within the article and Supplementary Materials.

## Supplementary Materials

The following supporting information can be downloaded at: https://www.mdpi.com/article/10.3390/molecules29122767/s1, Supplementary Materials (^1^H NMR and ^13^C NMR spectra of the target compounds **8a**–**z** and **11a**–**f**).
