# Supplementary material for: Design, Synthesis and Bioactivities of Novel Pyridyl Containing Pyrazole Oxime Ether Derivatives
Source: Molecules. 2024 Jun 11;29(12):2767. doi: 10.3390/molecules29122767 (PMC11206151; doi:10.3390/molecules29122767)
Supplement: Supplementary file 1 [file molecules-29-02767-s001.zip › molecules-3033284-supplementary.pdf]

molecules

Supporting Information

Design, Synthesis and Bioactivities of Novel Pyridyl Containing Pyrazole Oxime Ether Derivatives

Jie He [1,†], Beibei Zhou [1,†], Xinjuan Wang [1], Qi Chen [1], Xiaoqian Jiang [1], Ting Kong [1], Long Yao [2,*], Yingying Zhao [1], Rong Chen [1], Ying Xu [1] and Hong Dai [1,*]

Received: date ; Accepted: date ; Published: date
Academic Editor: Jian-Quan Weng

[1] College of Chemistry and Chemical Engineering, Nantong University, Nantong 226019, China
[2] Analysis and Testing Center, Nantong University, Nantong 226019, China
† These authors contributed equally to this work.
* Correspondence: yaolong@ntu.edu.cn (L.Y.); dh123@ntu.edu.cn (H.D.); Tel./Fax: +86-513-8501-2955 (L.Y.) ; +86-513-8501-2945 (H.D.)

The ^{1}H-NMR, ^{13}C-NMR and HRMS spectra of compounds **8a**-**z**, and **11a**-**f** were listed below:

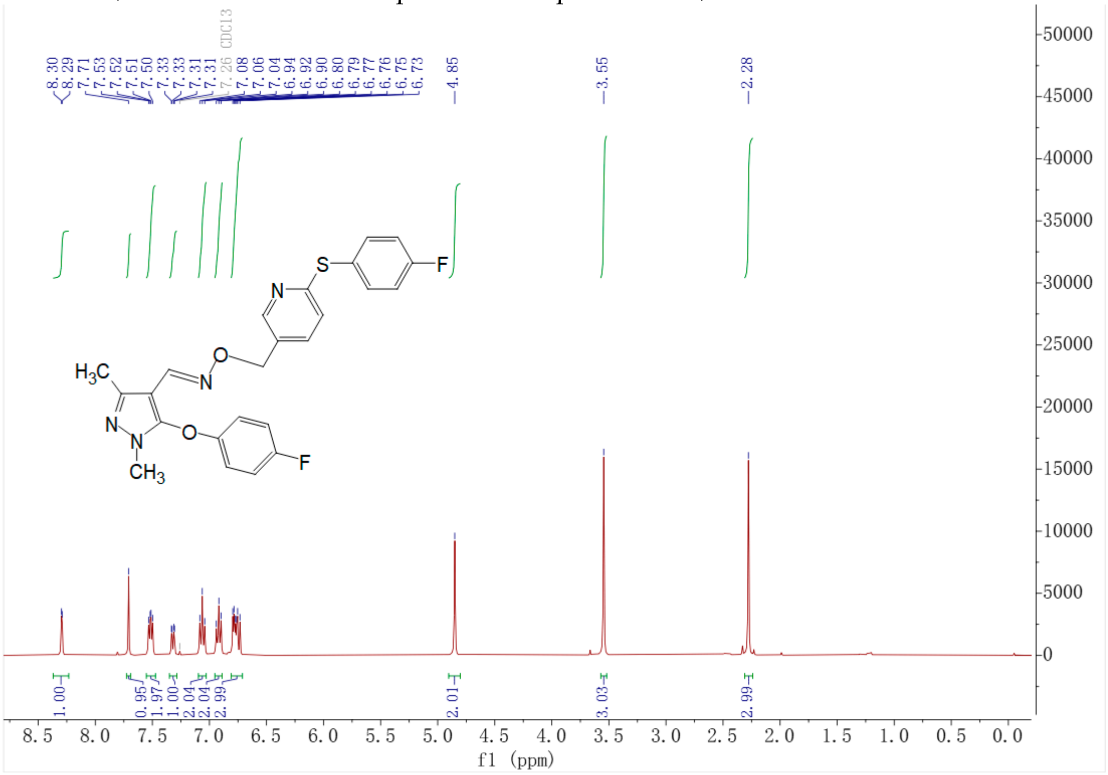

Figure S1. ^{1}H-NMR of compound **8a** (400 MHz, $CDCl_3$)

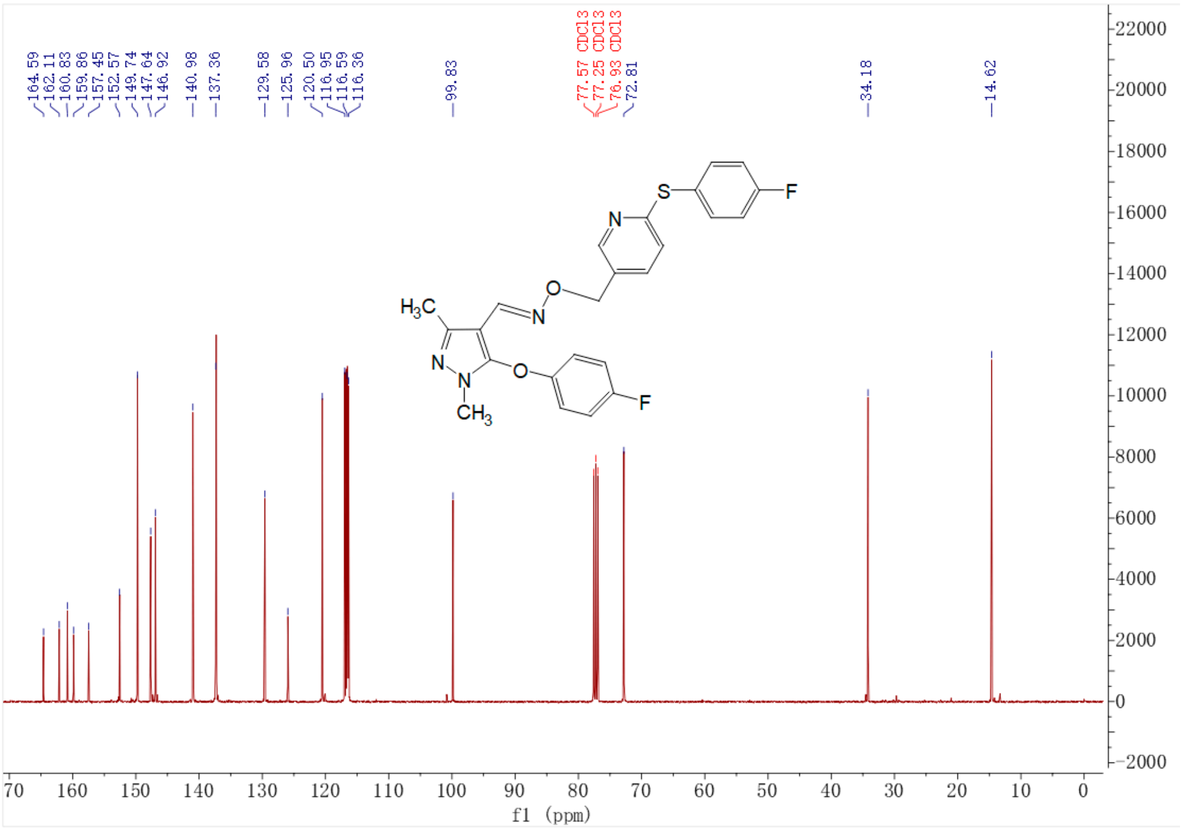

Figure S2. ^{13}C-NMR of compound **8a** (101 MHz, $CDCl_3$)

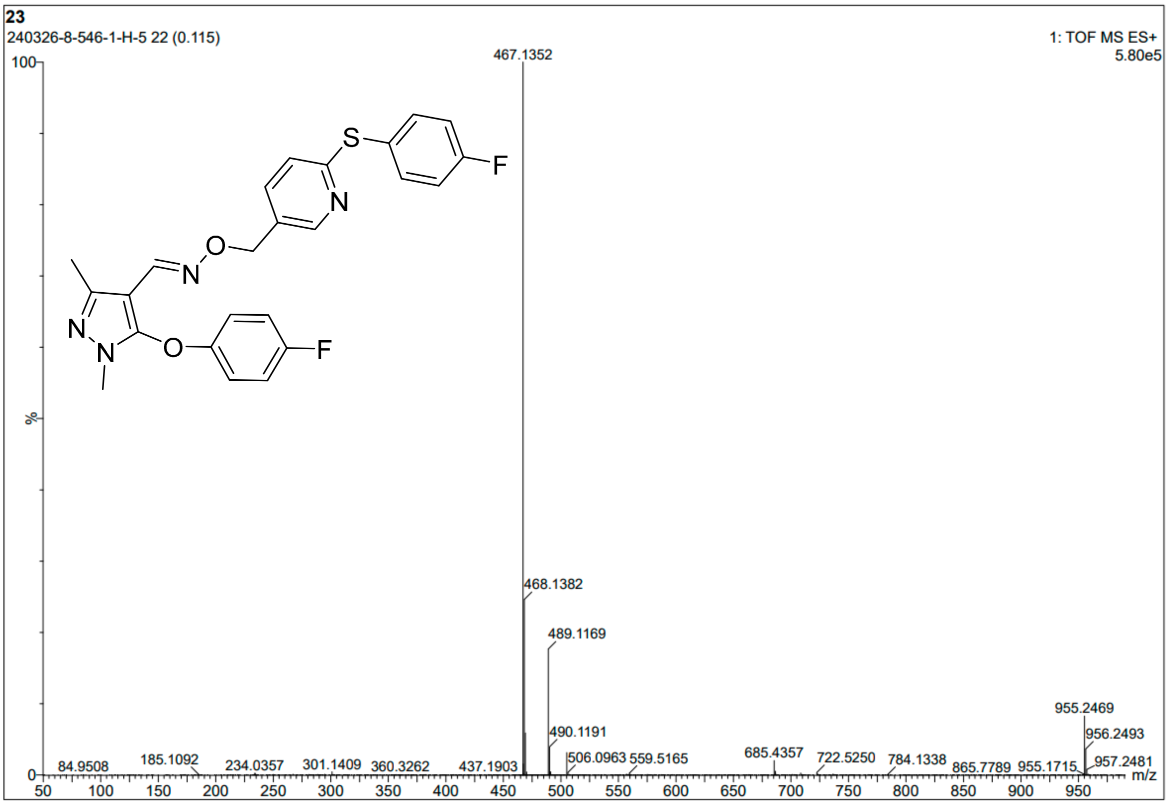

Figure S3. HRMS of compound **8a**

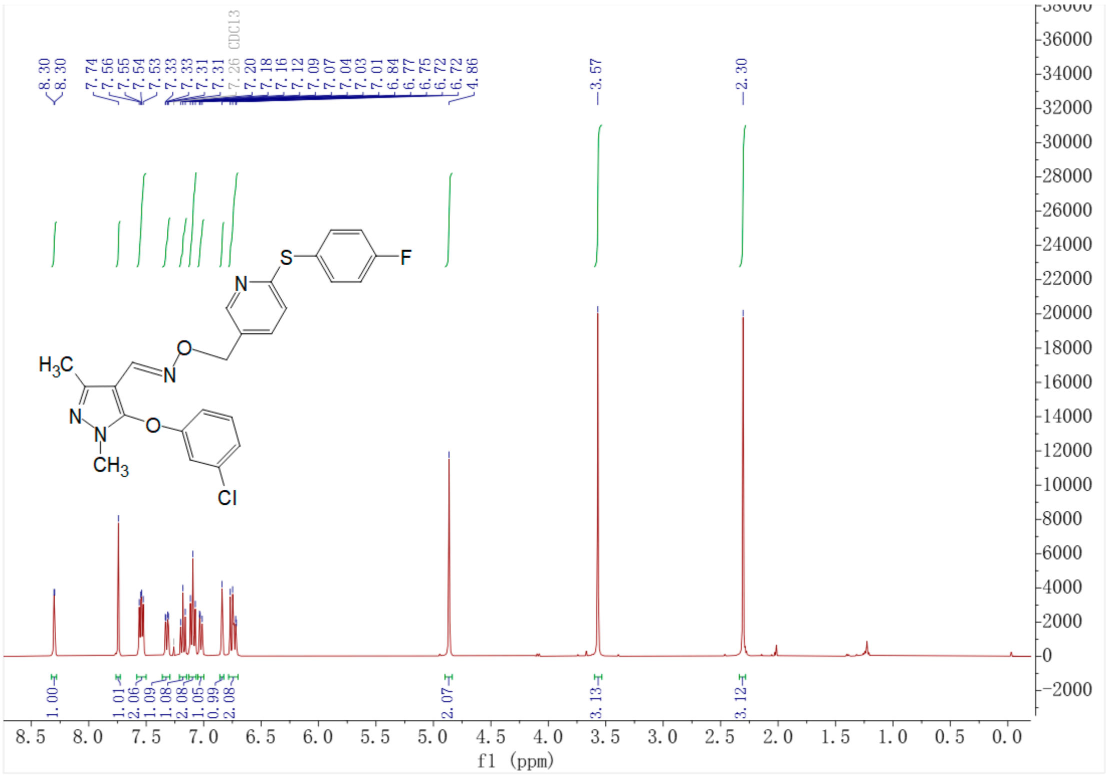

Figure S4. ¹H-NMR of compound **8b** (400 MHz, $CDCl_3$)

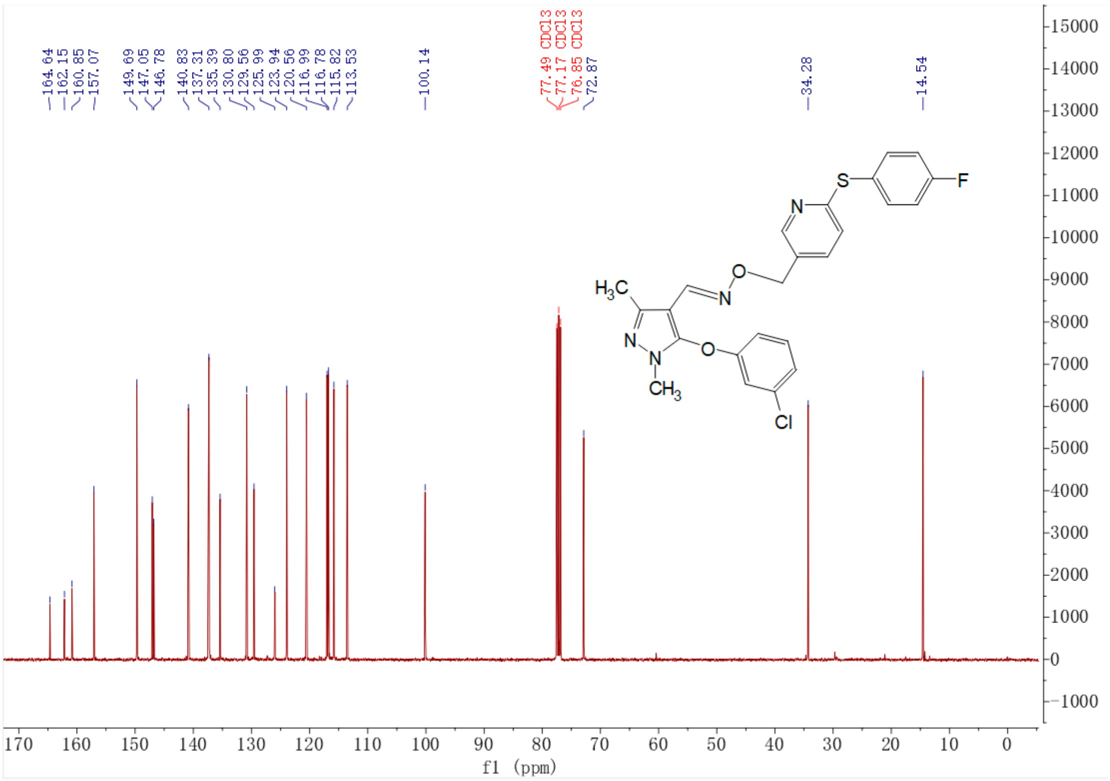

Figure S5. ^{13}C-NMR of compound **8b** (101 MHz, $CDCl_3$)

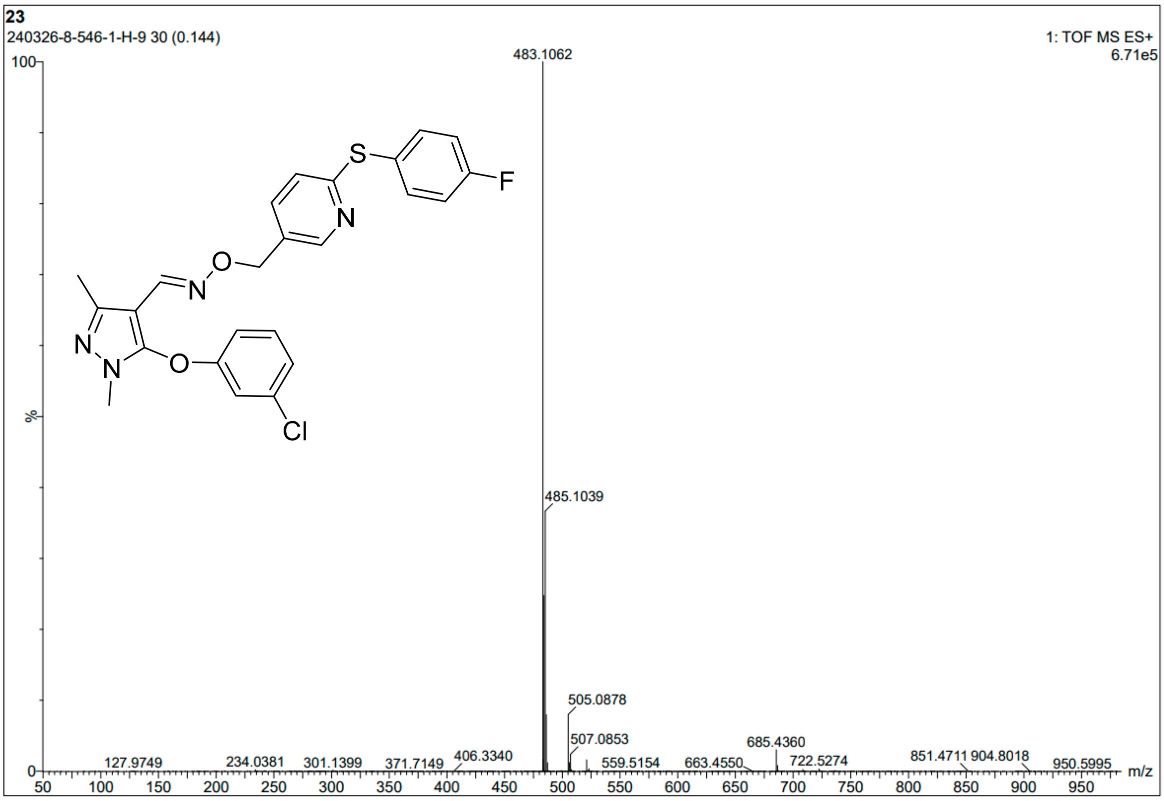

Figure S6. HRMS of compound **8b**

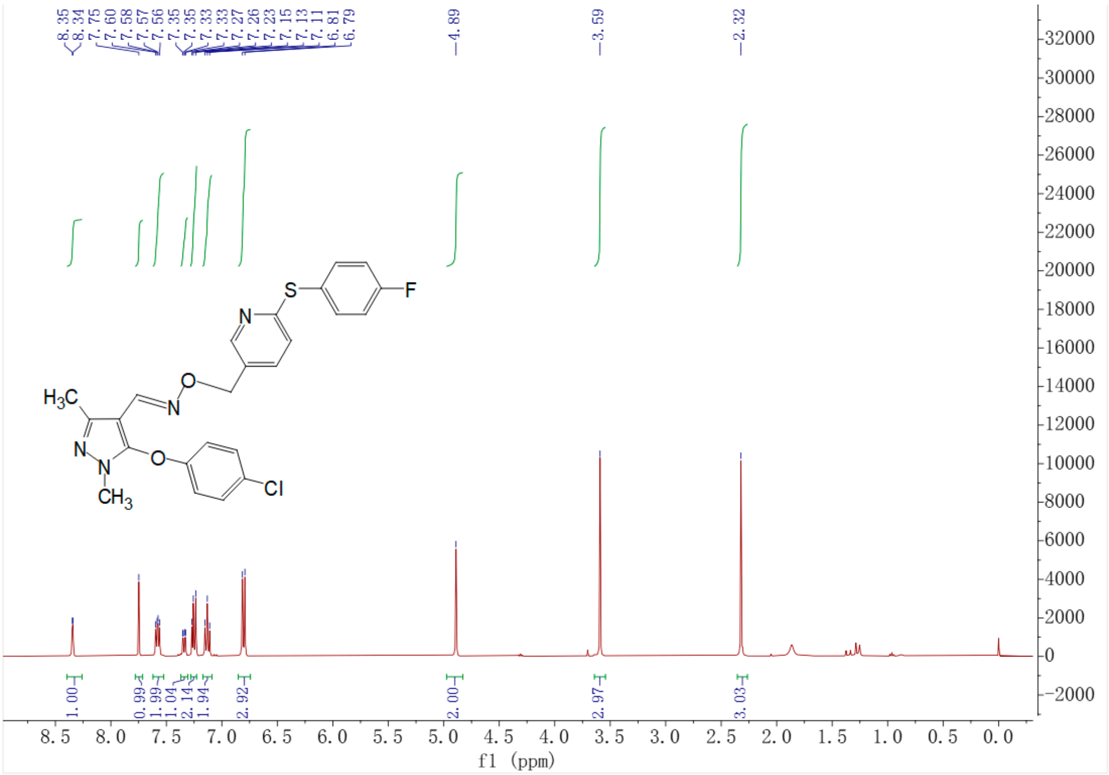

Figure S7. ^{1}H-NMR of compound **8c** (400 MHz, $CDCl_3$)

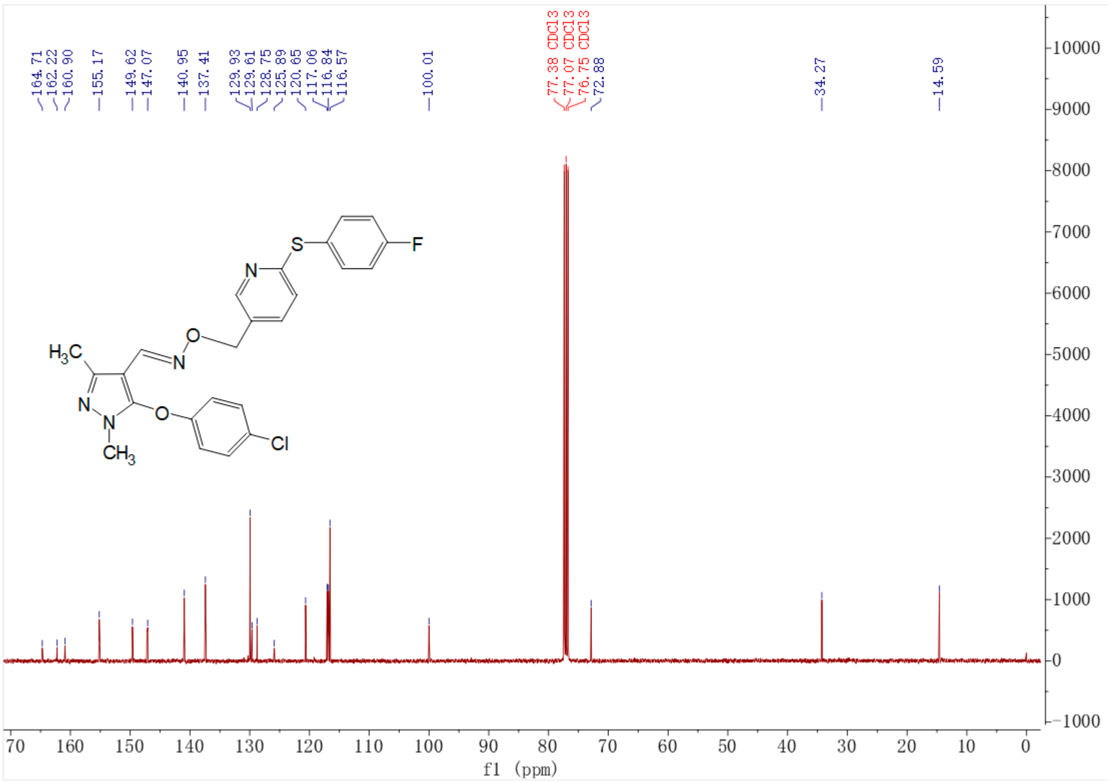

Figure S8. ^{13}C-NMR of compound **8c** (101 MHz, $CDCl_3$)

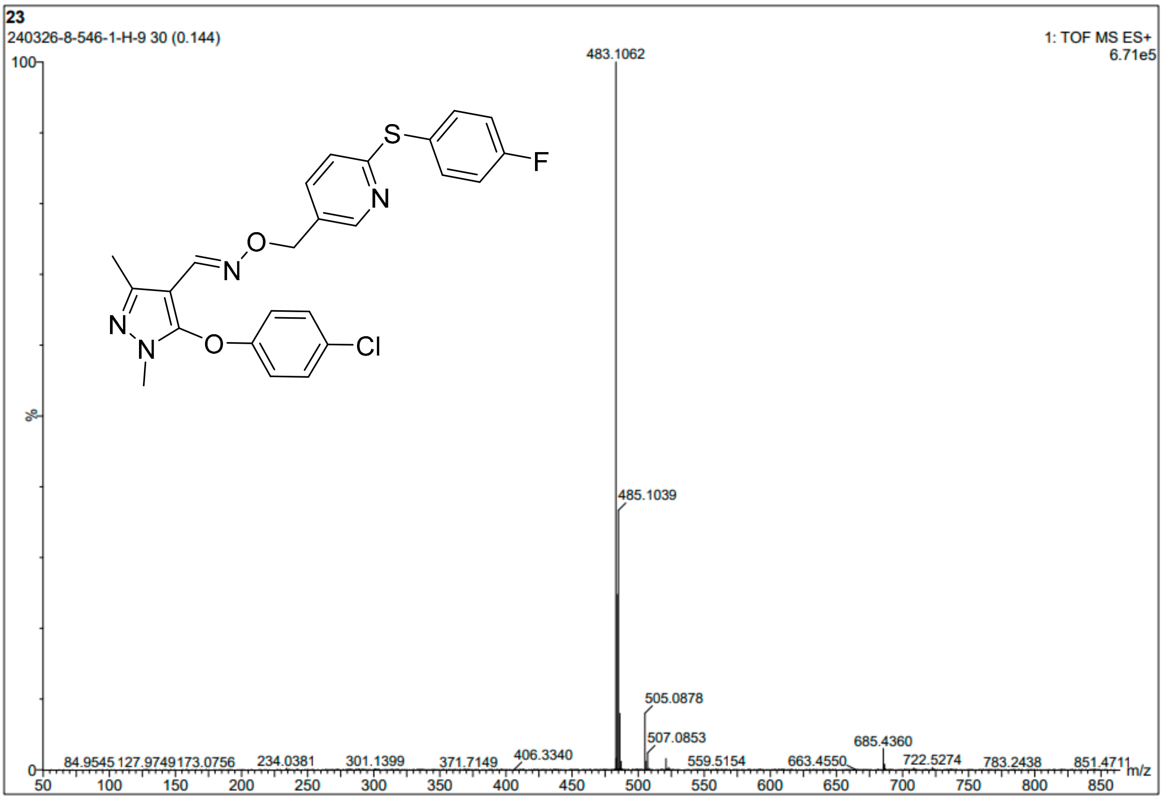

Figure S9. HRMS of compound **8c**

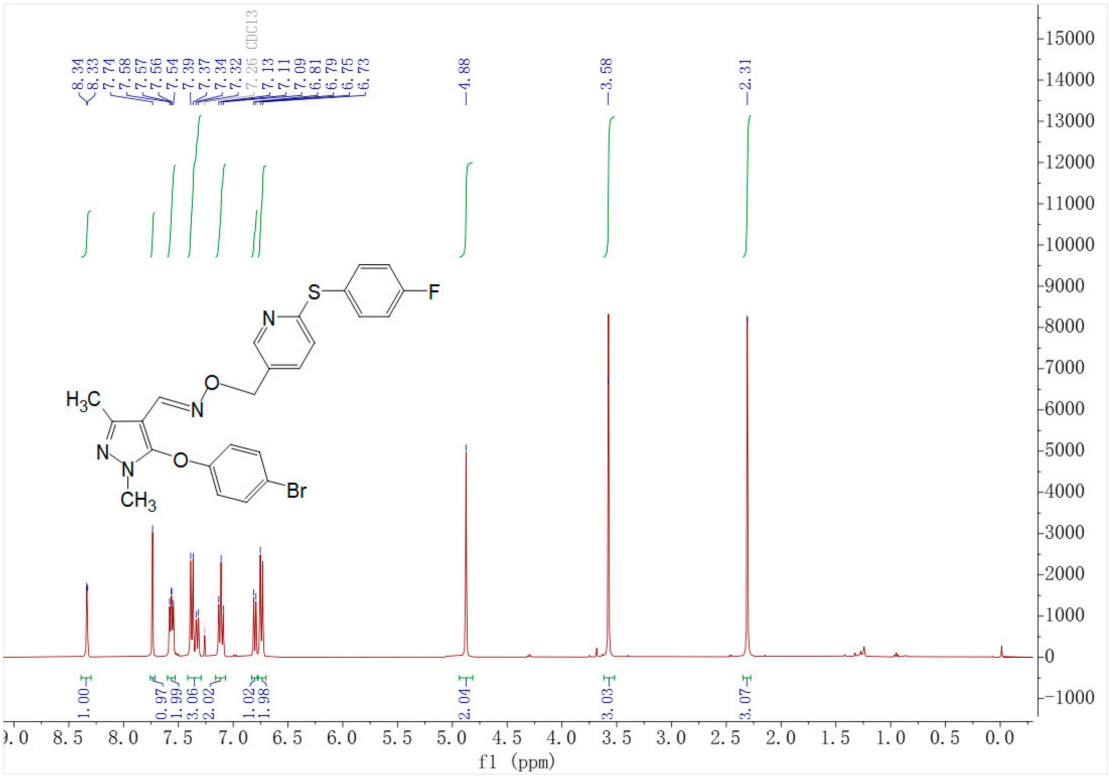

Figure S10. ^{1}H-NMR of compound **8d** (400 MHz, $CDCl_3$)

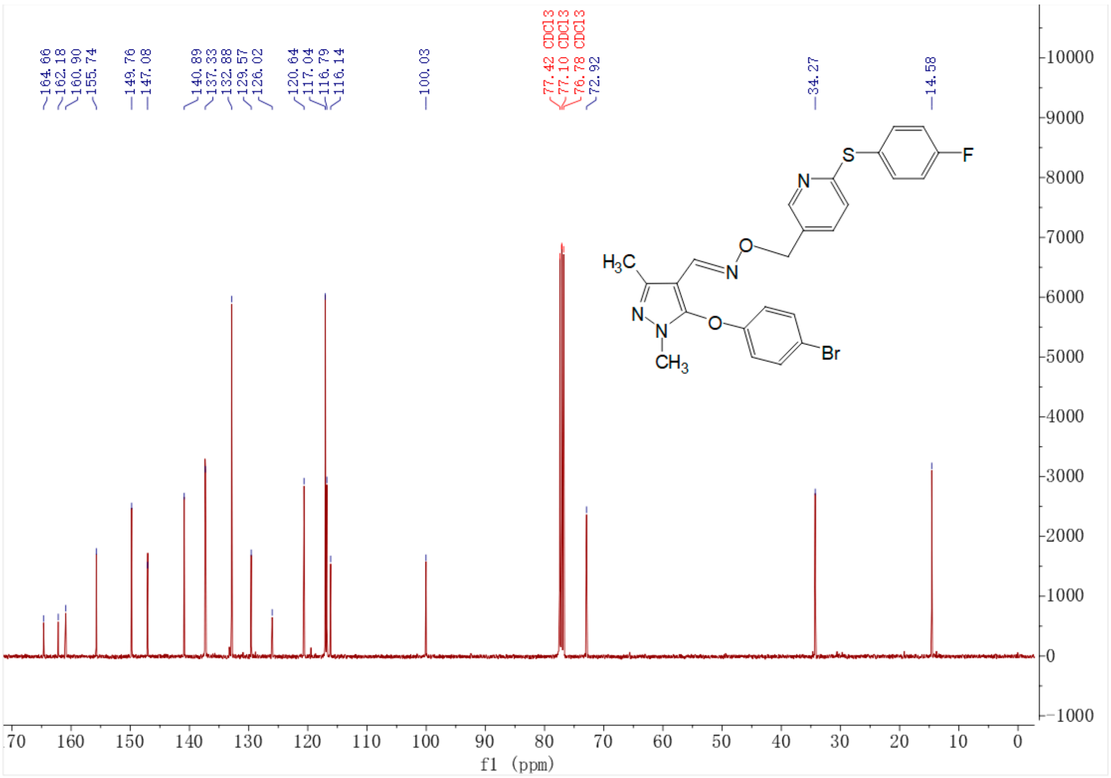

Figure S11. ¹³C-NMR of compound **8d** (101 MHz, $CDCl_3$)

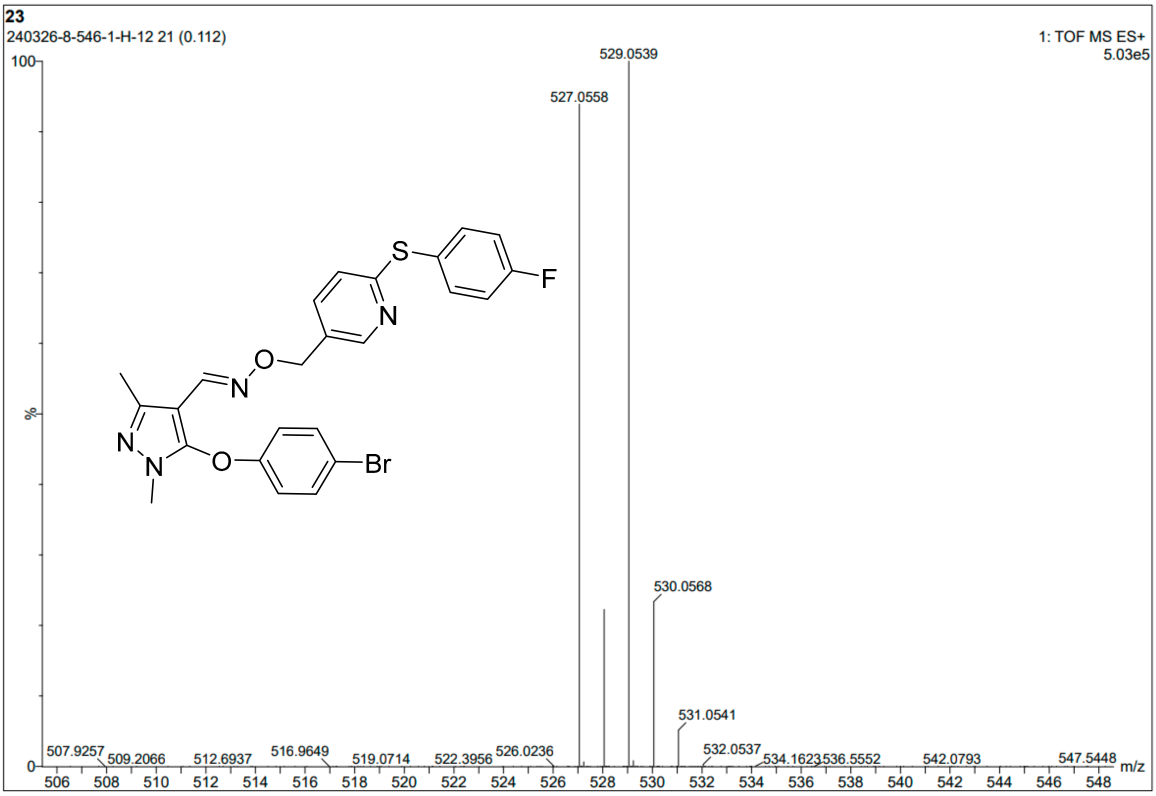

Figure S12. HRMS of compound **8d**

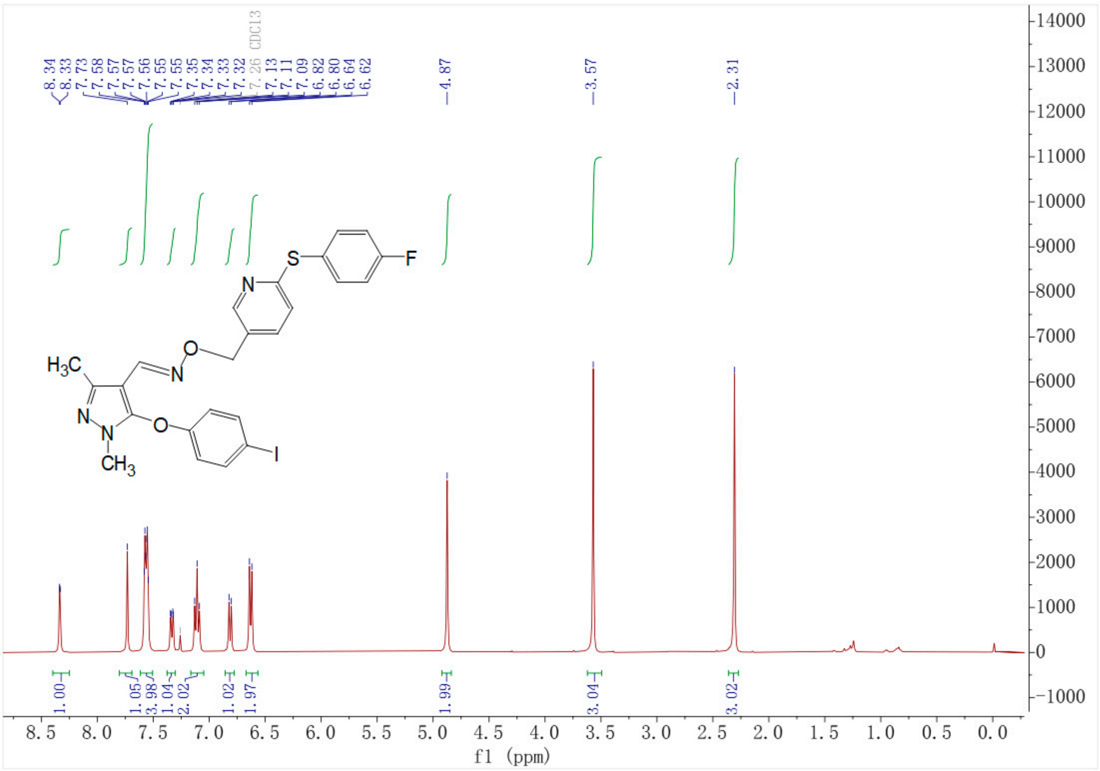

Figure S13. ^{1}H-NMR of compound **8e** (400 MHz, $CDCl_3$)

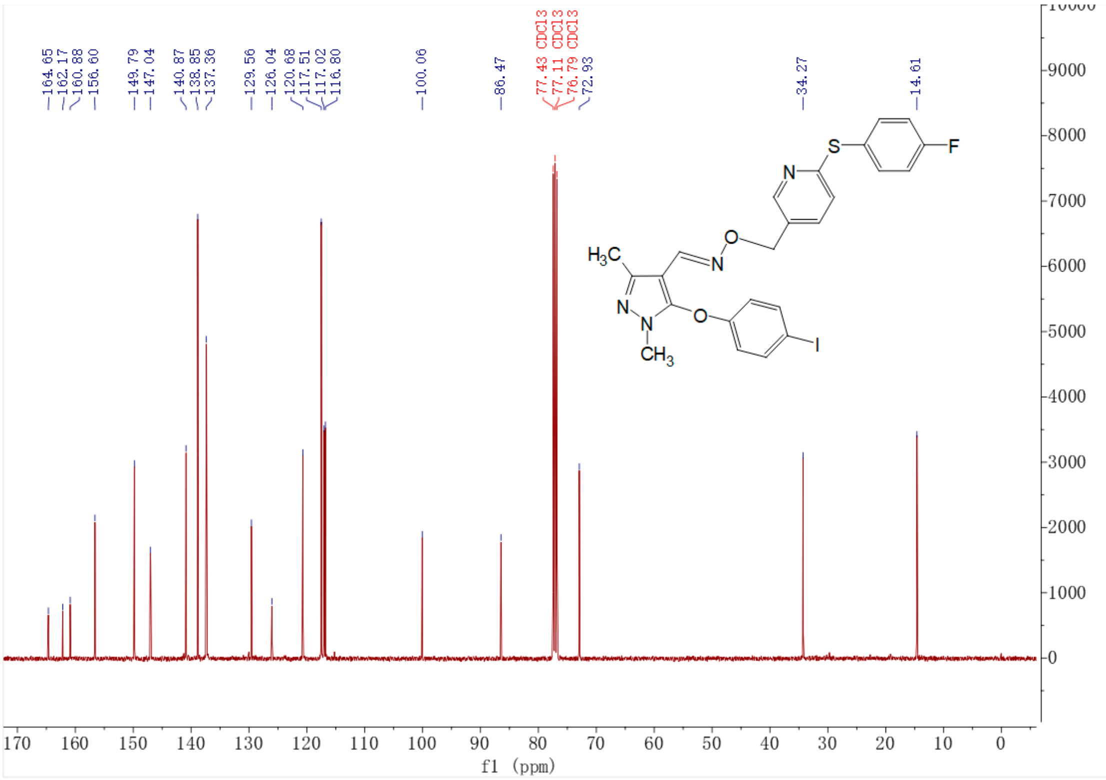

Figure S14. ^{13}C-NMR of compound **8e** (101 MHz, $CDCl_3$)

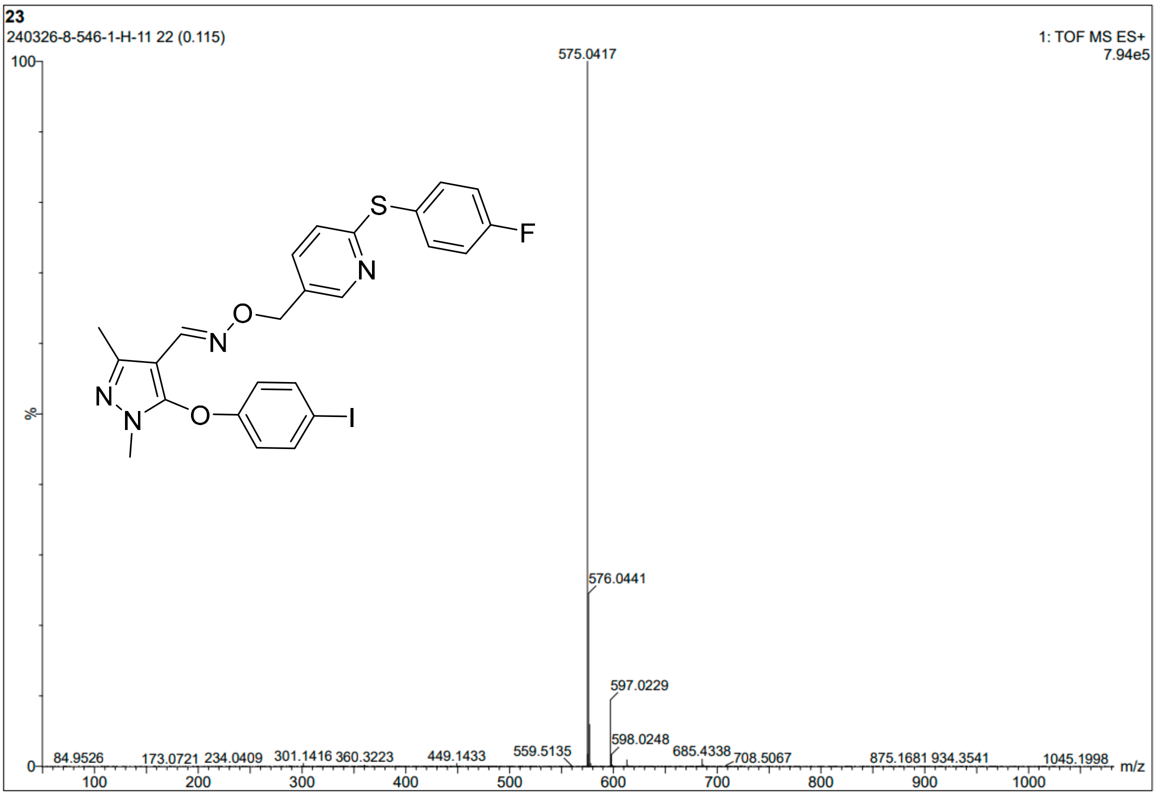

Figure S15. HRMS of compound **8e**

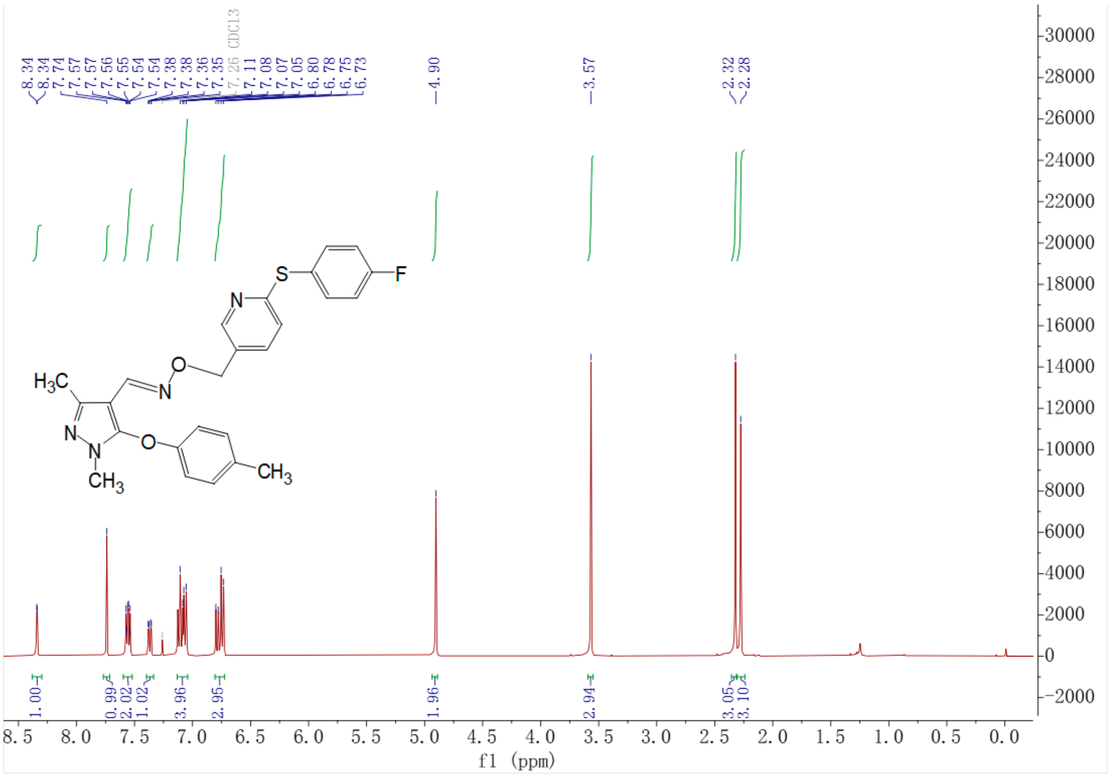

Figure S16. ^{1}H-NMR of compound **8f** (400 MHz, $CDCl_3$).

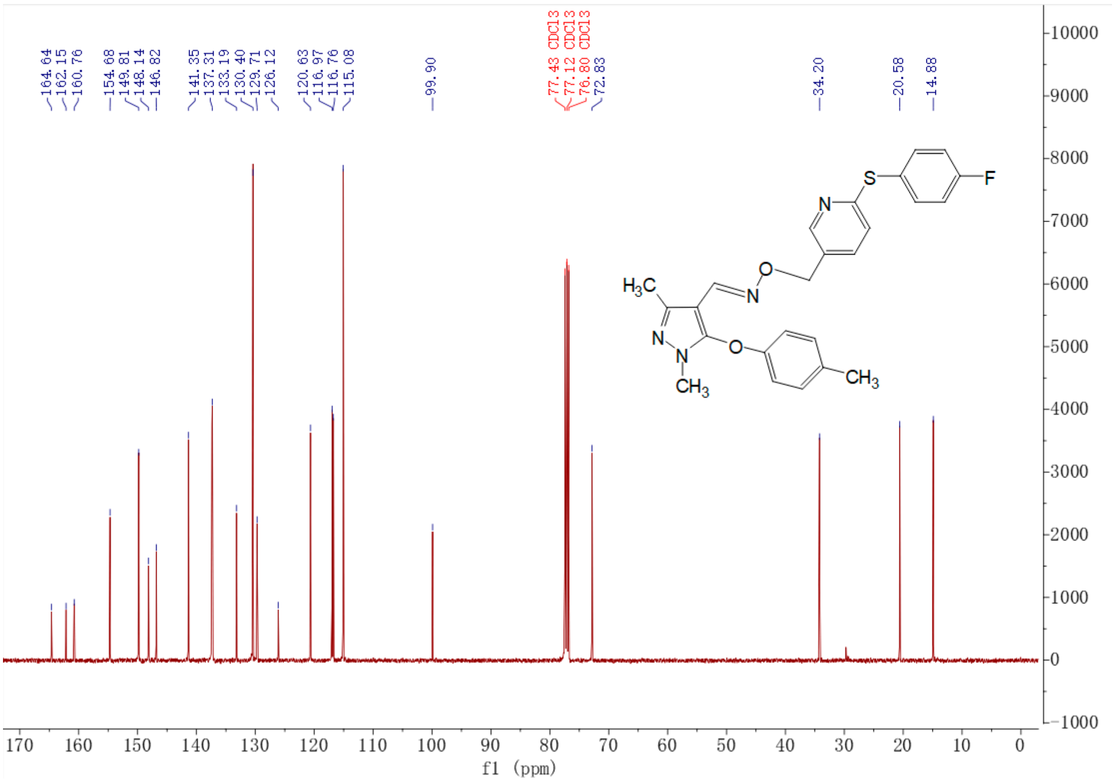

Figure S17. ^{13}C-NMR of compound **8f** (101 MHz, $CDCl_3$)

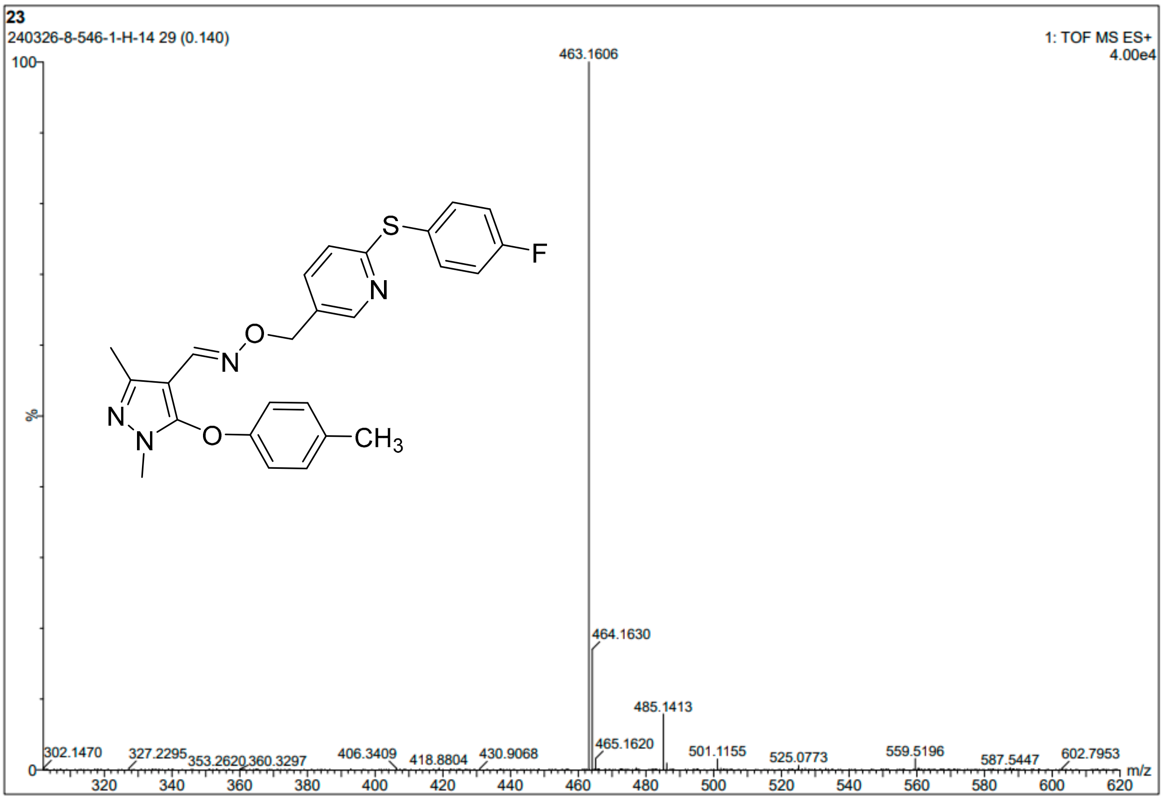

Figure S18. HRMS of compound **8f**

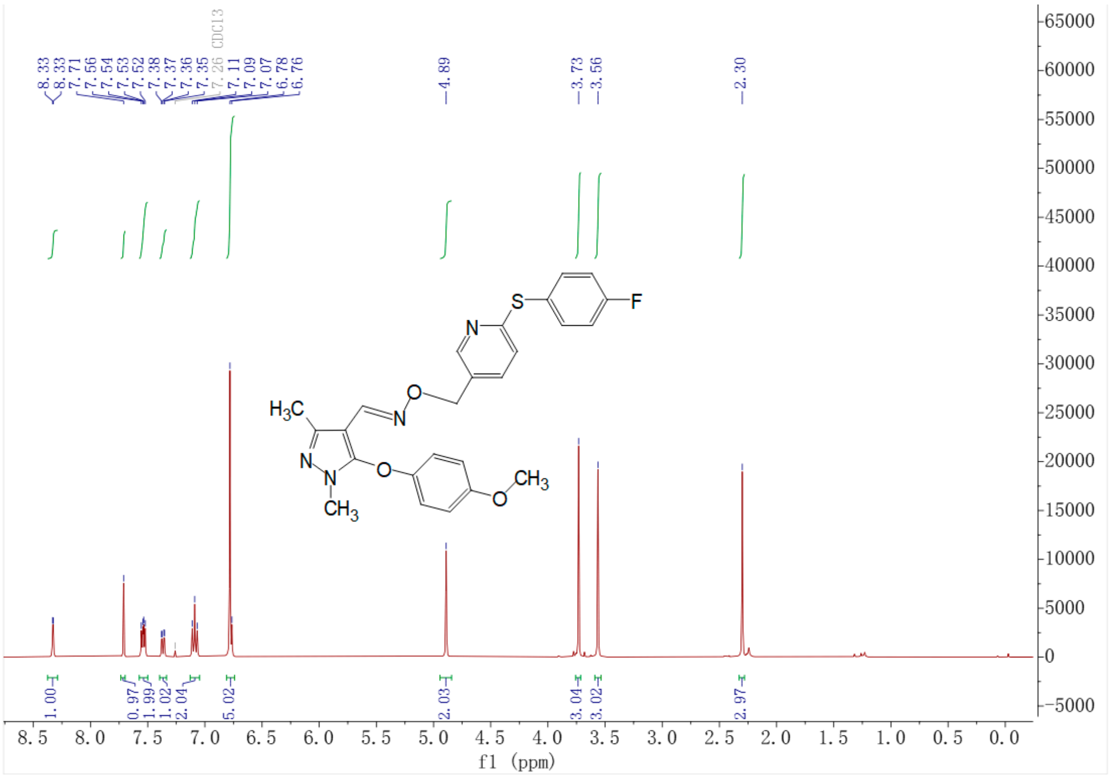

Figure S19. ^{1}H-NMR of compound **8g** (400 MHz, $CDCl_3$)

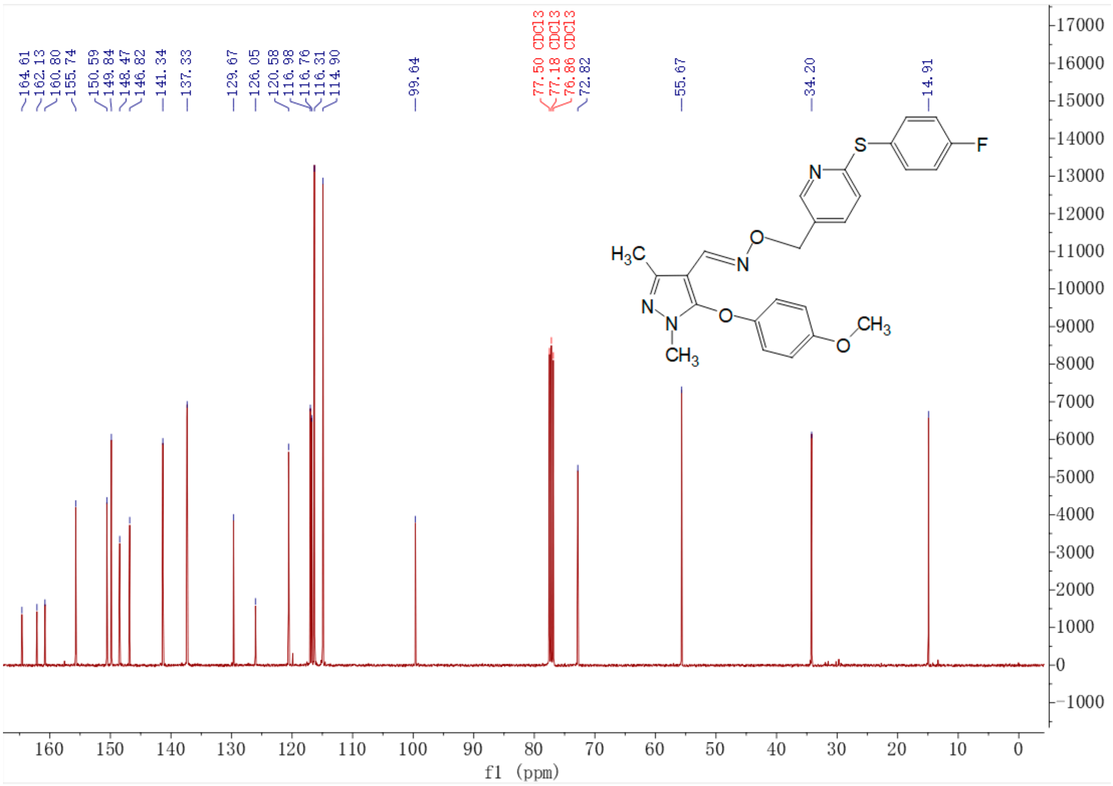

Figure S20. ^{13}C-NMR of compound **8g** (101 MHz, $CDCl_3$)

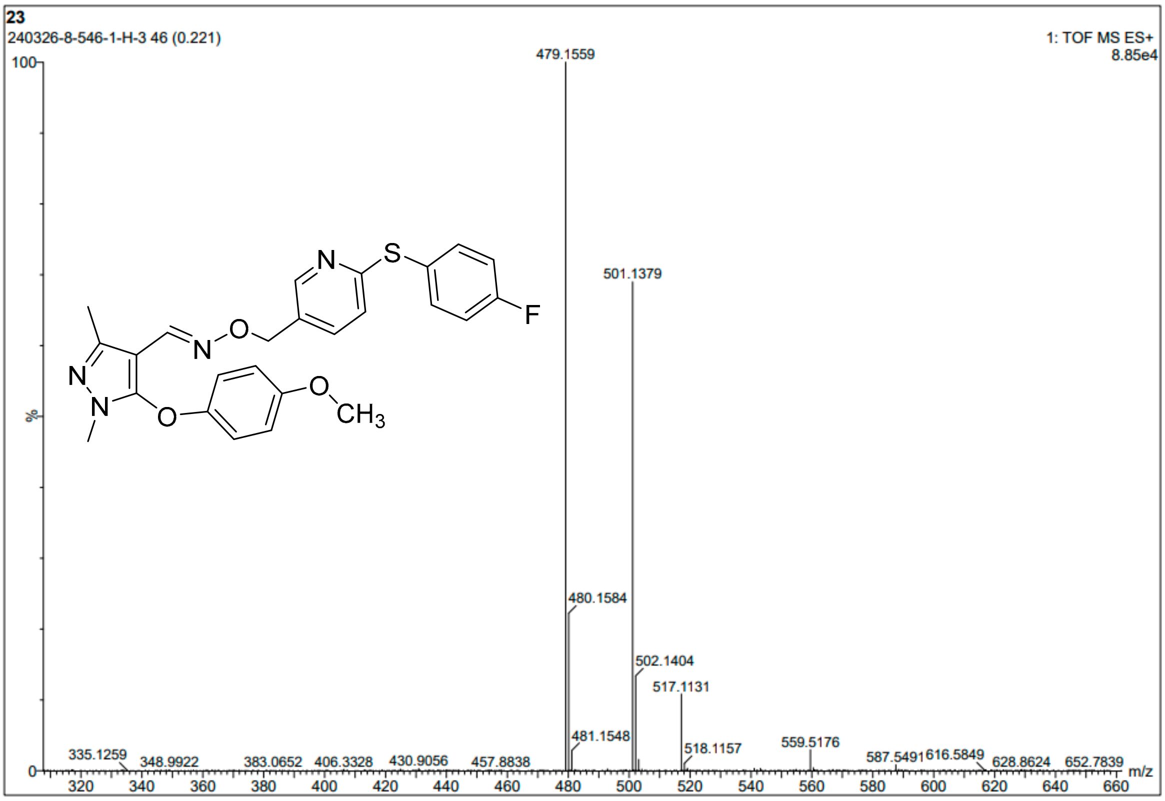

Figure S21. HRMS of compound **8g**

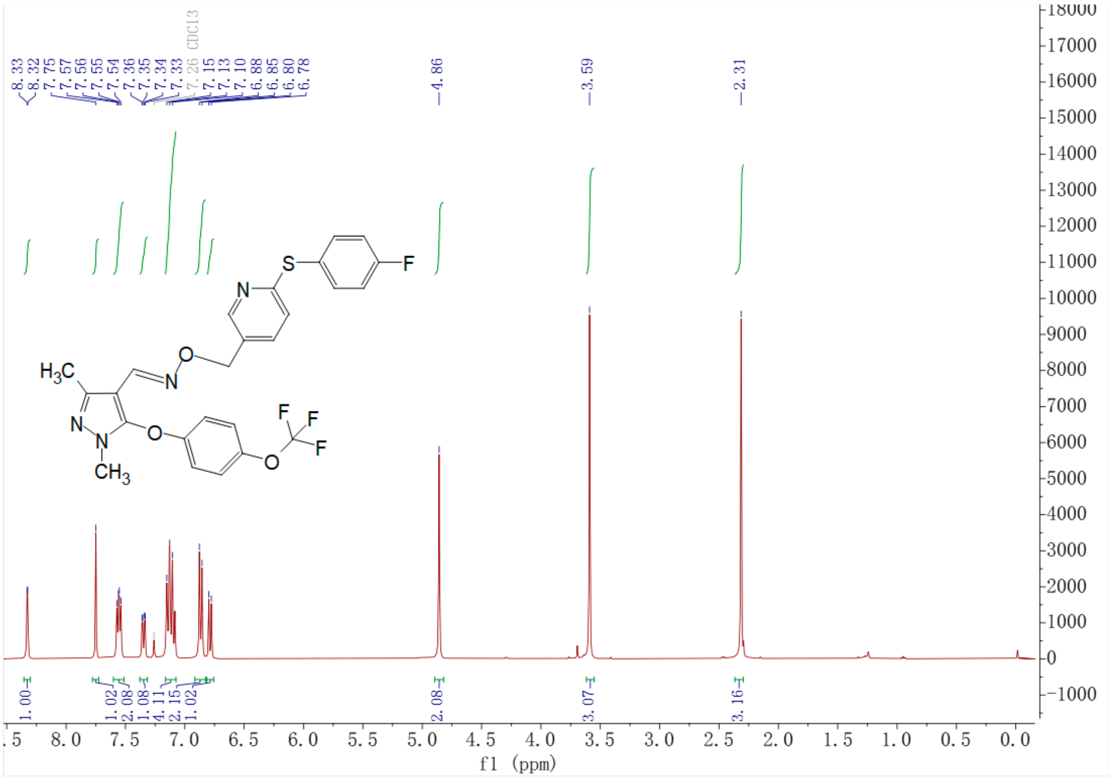

Figure S22. ^{1}H-NMR of compound **8h** (400 MHz, $CDCl_3$)

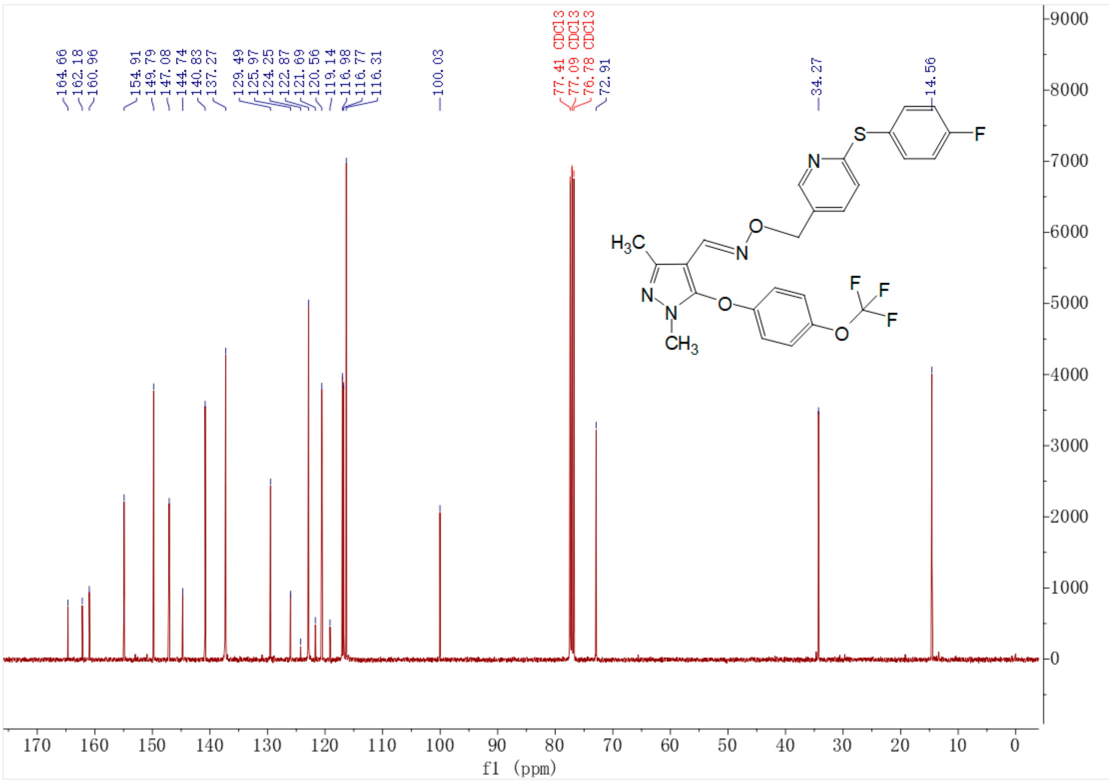

Figure S23. ^{13}C-NMR of compound **8h** (101 MHz, $CDCl_3$)

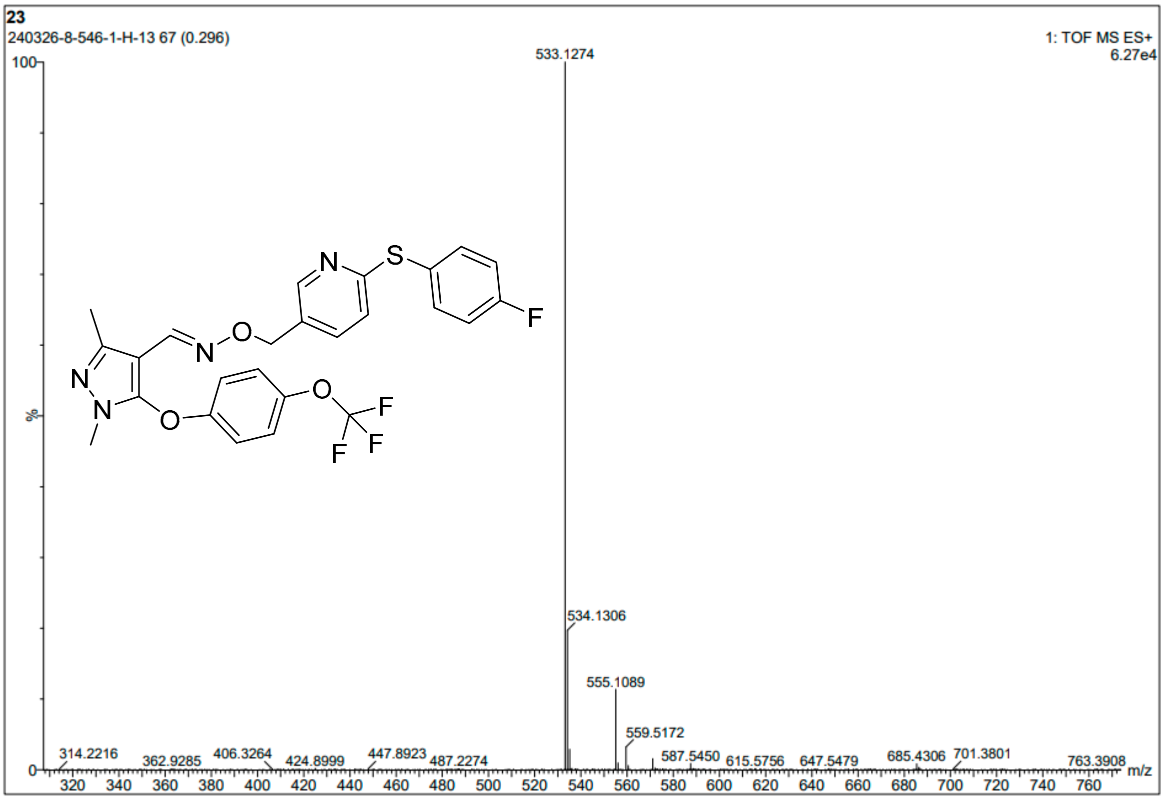

Figure S24. HRMS of compound **8h**

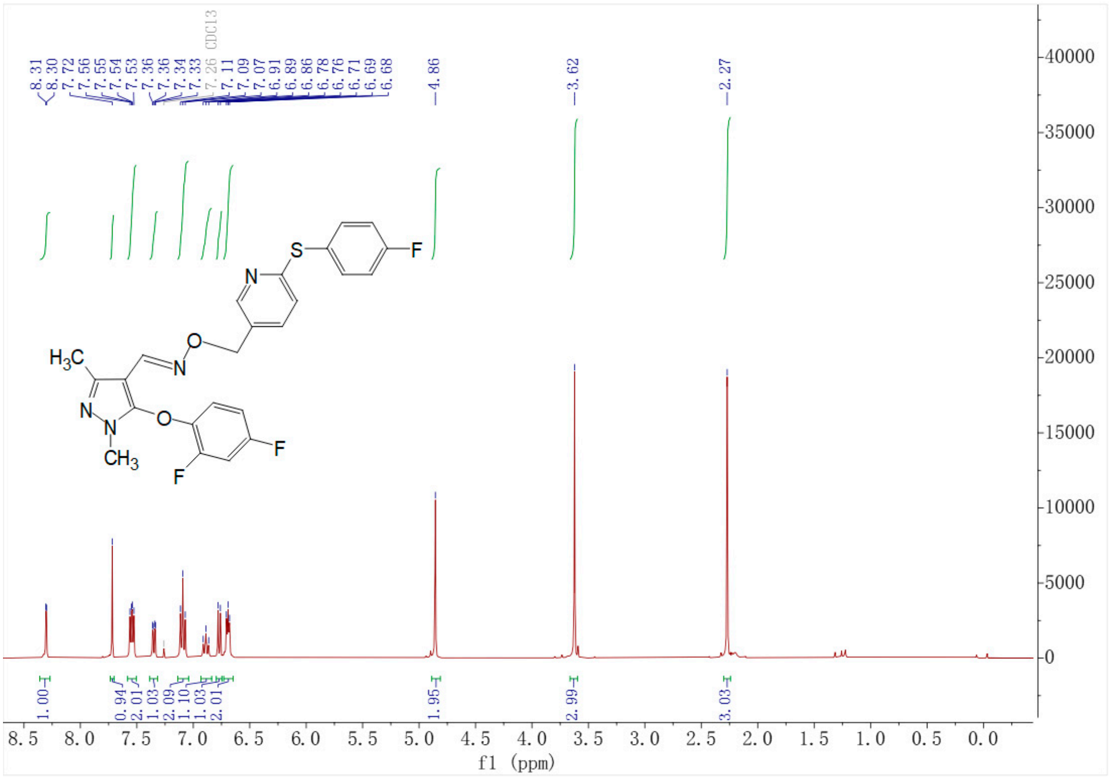

Figure S25. ^{1}H-NMR of compound **8i** (400 MHz, $CDCl_3$)

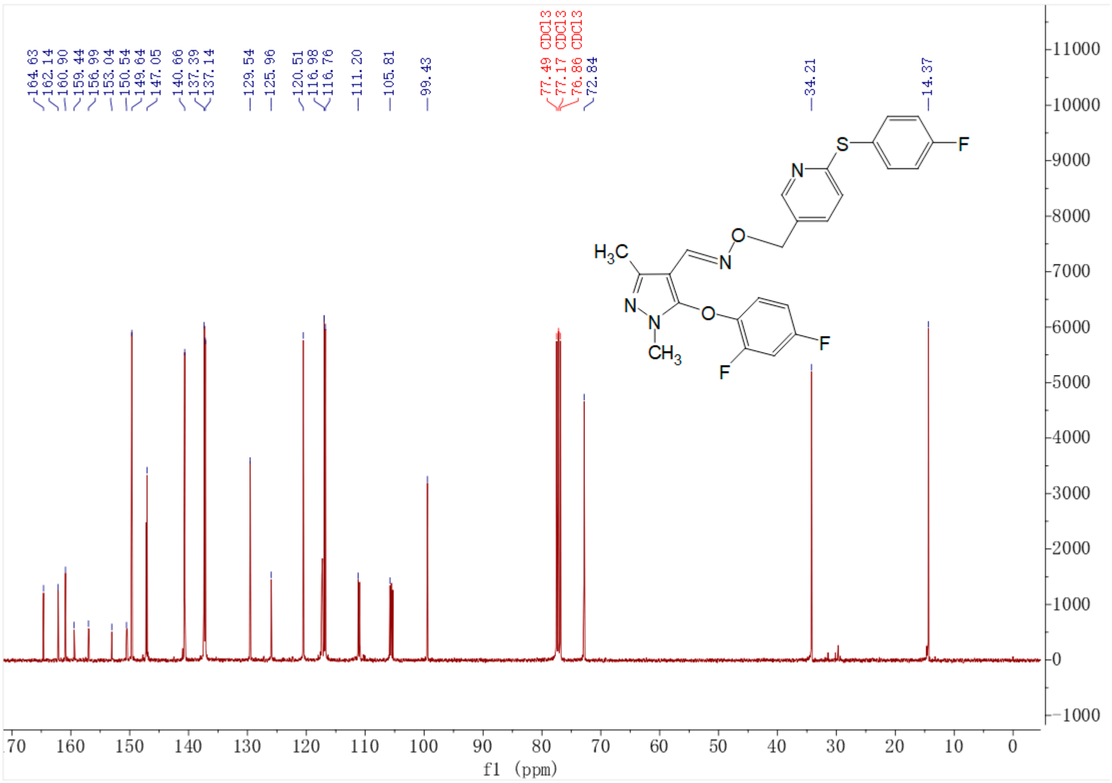

Figure S26. ^{13}C-NMR of compound **8i** (101 MHz, $CDCl_3$)

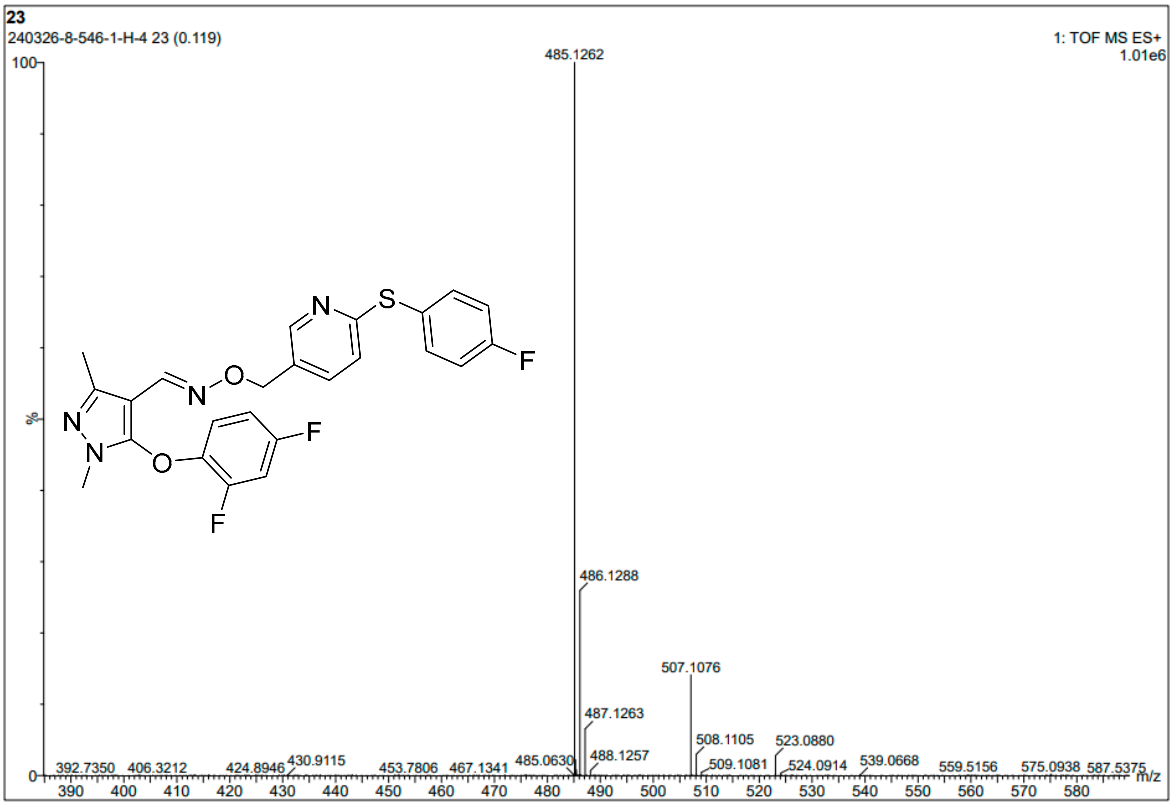

Figure S27. HRMS of compound **8i**

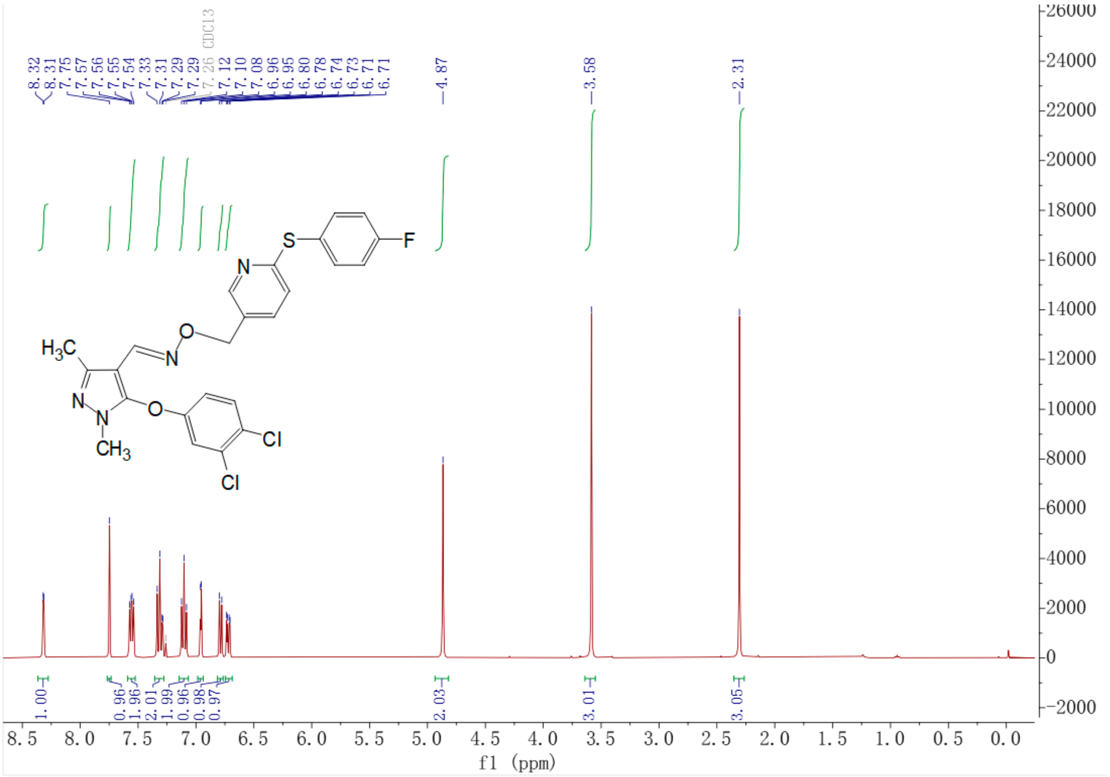

Figure S28. ^{1}H-NMR of compound **8j** (400 MHz, $CDCl_3$)

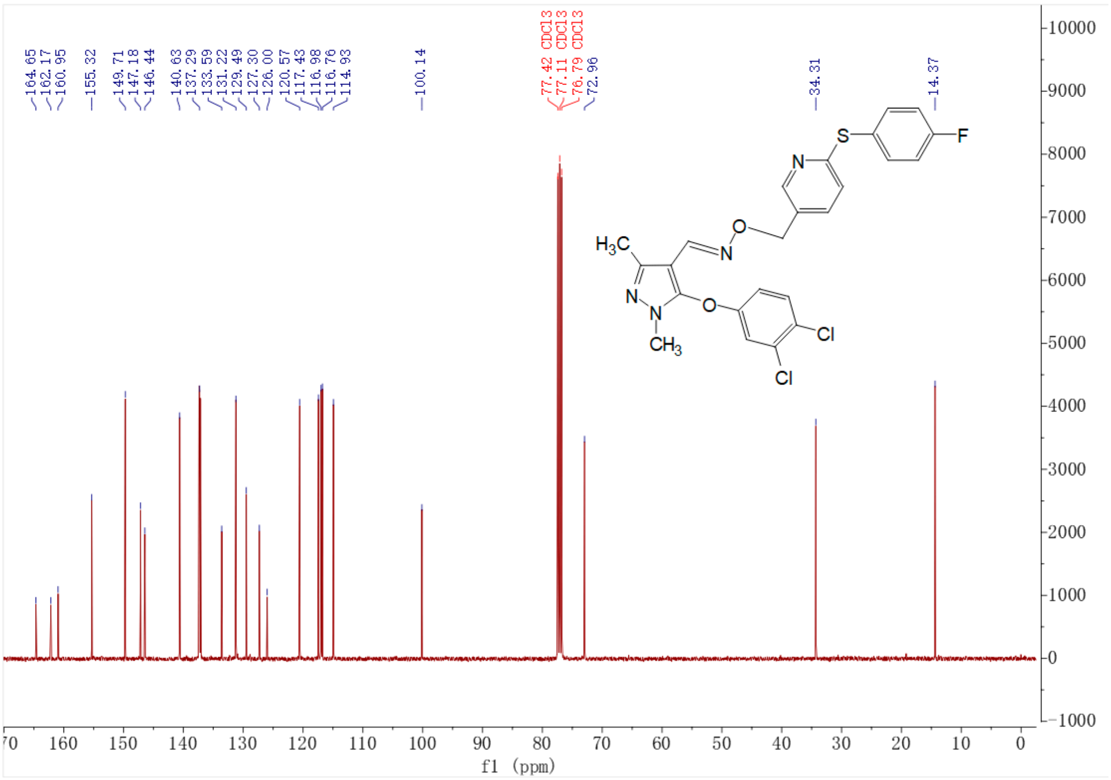

Figure S29. ^{13}C-NMR of compound **8j** (101 MHz, $CDCl_3$)

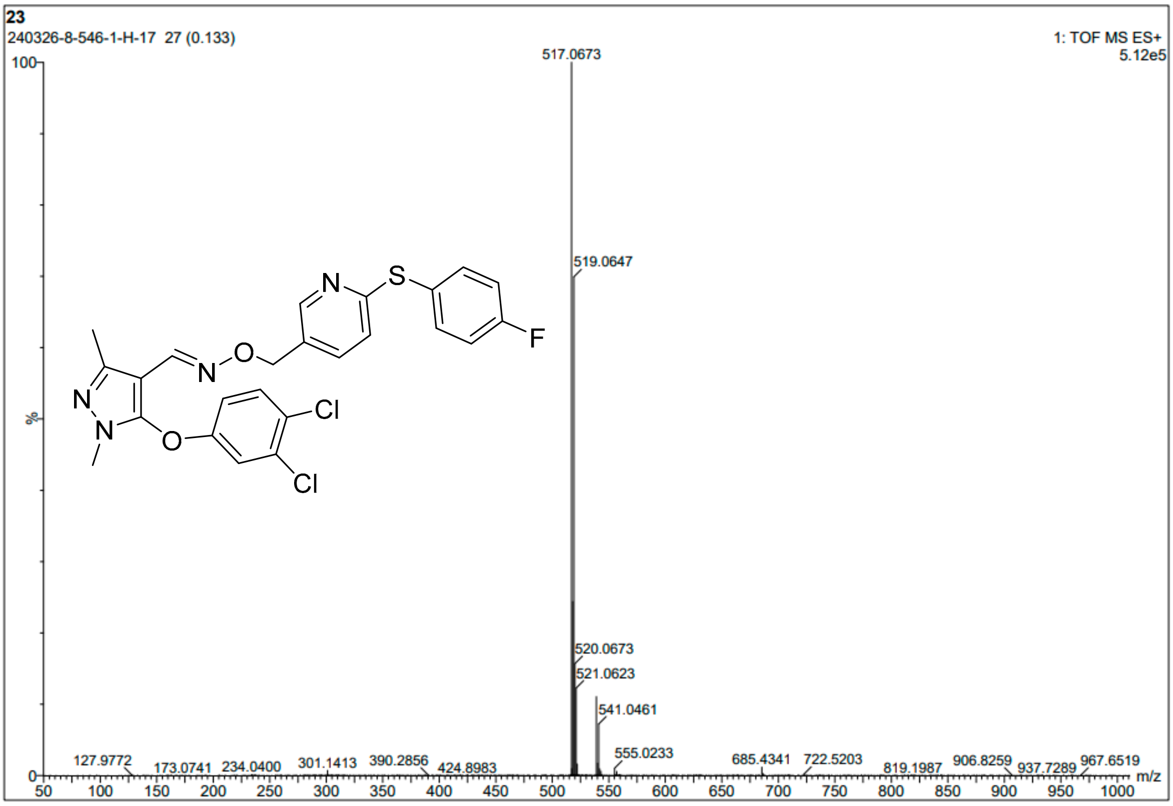

Figure S30. HRMS of compound **8j**

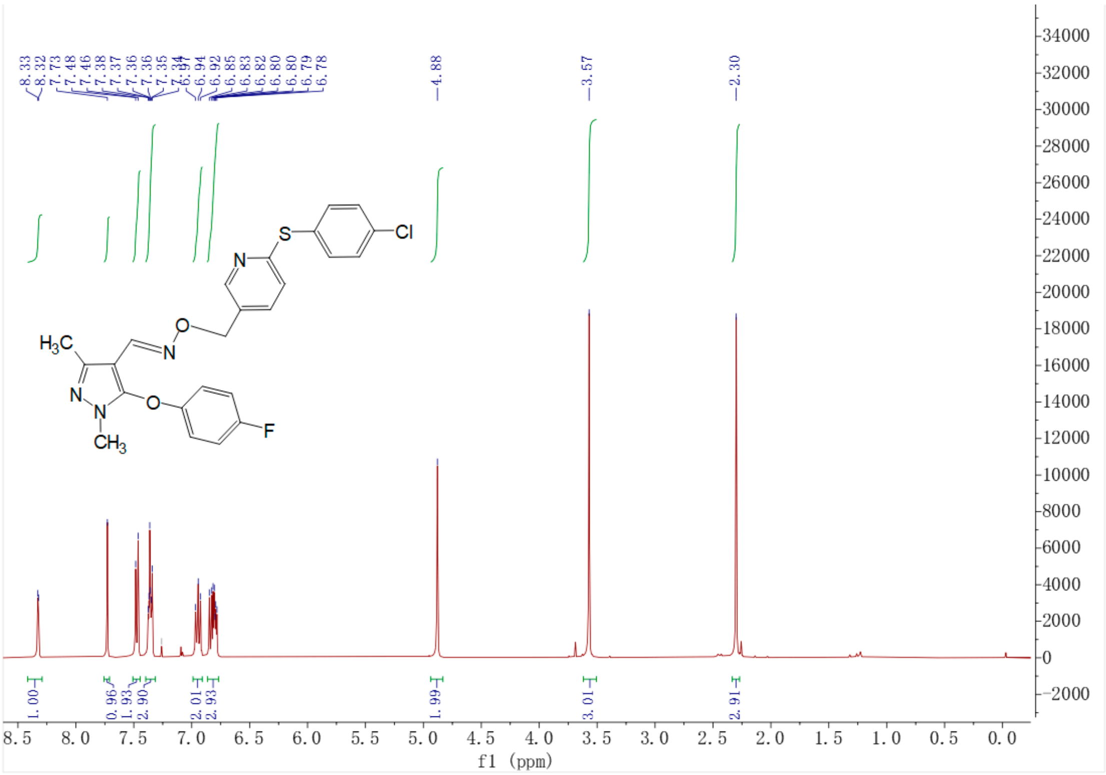

Figure S31. ^{1}H-NMR of compound **8k** (400 MHz, $CDCl_3$)

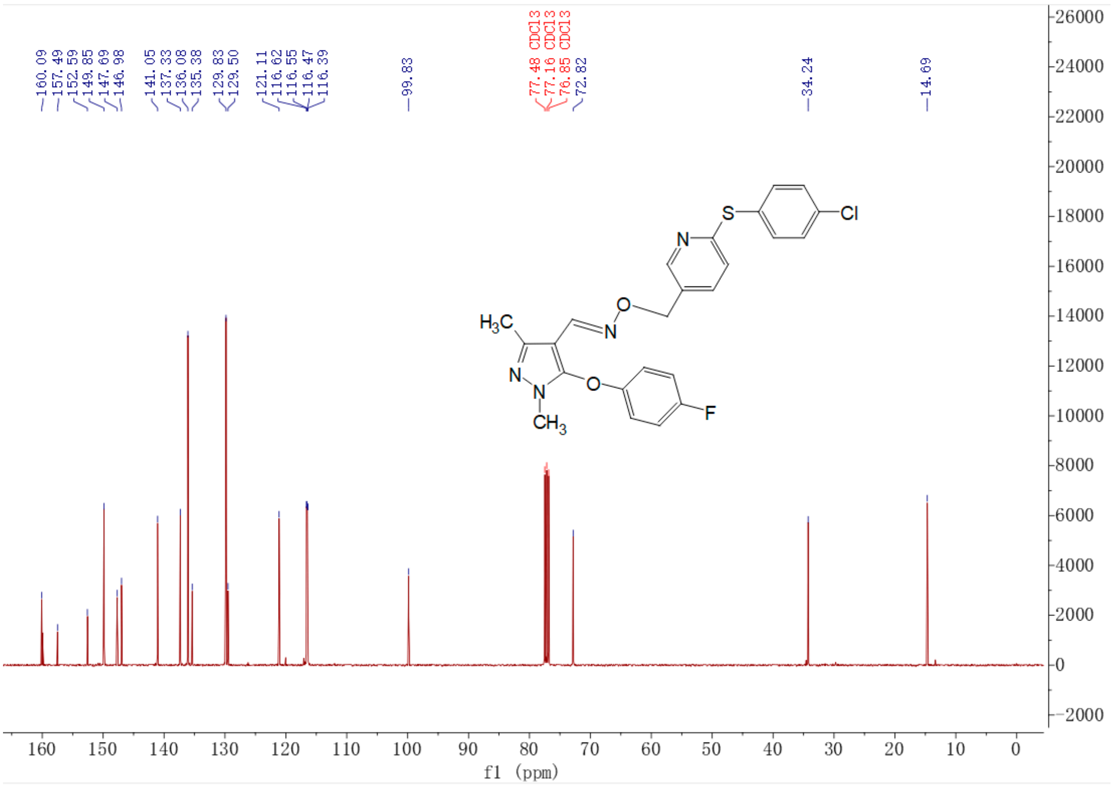

Figure S32. ^{13}C-NMR of compound**8k** (101 MHz, $CDCl_3$)

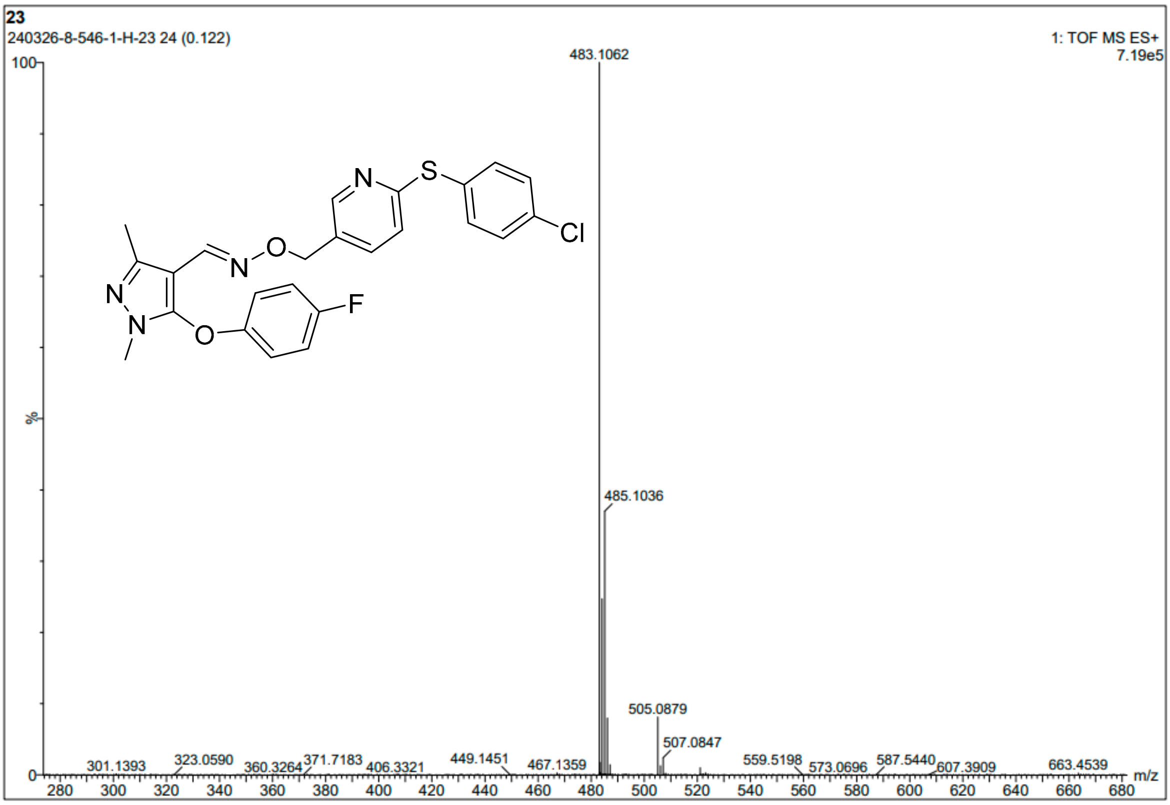

Figure S33. HRMS of compound **8k**

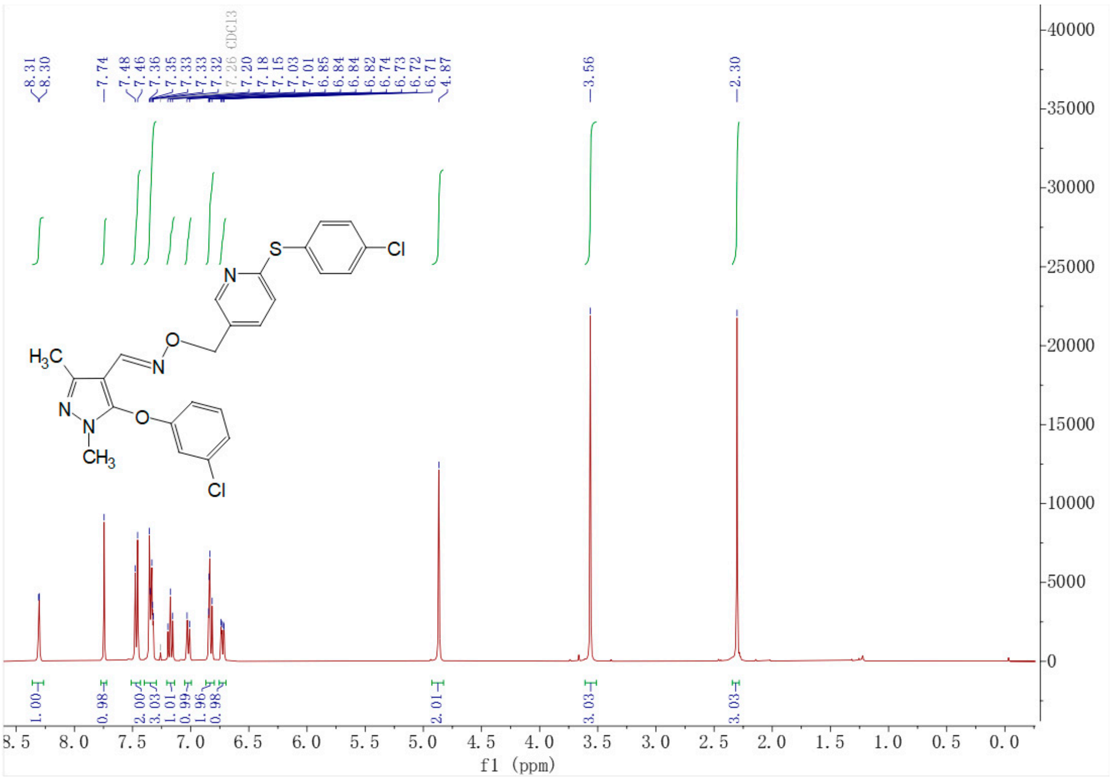

Figure S34. ^{1}H-NMR of compound **8l** (400 MHz, $CDCl_3$)

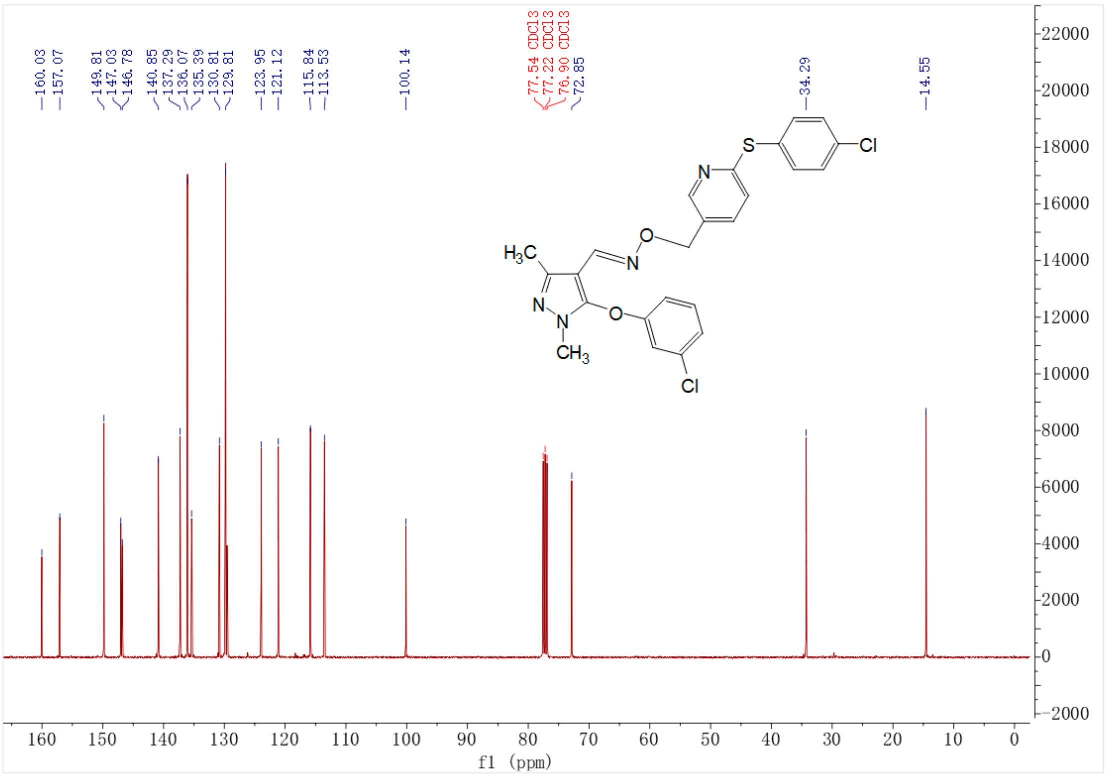

Figure S35. ^{13}C-NMR of compound **8l** (101 MHz, $CDCl_3$)

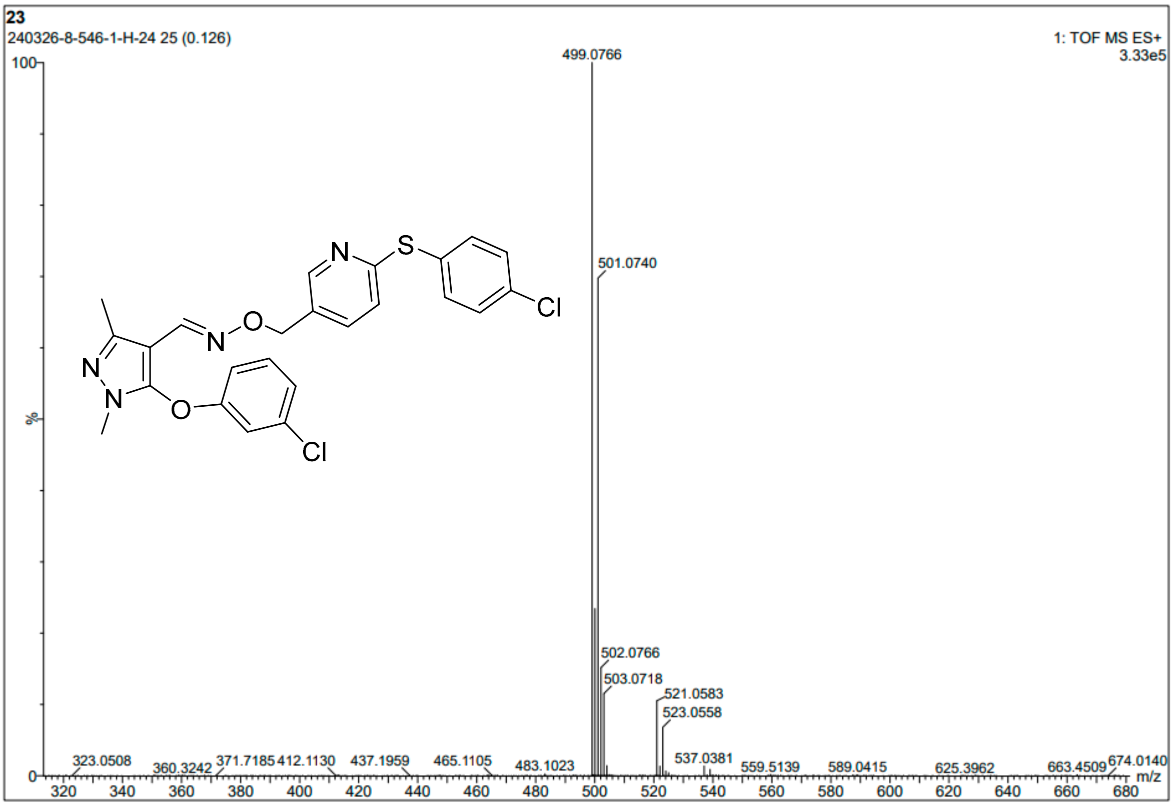

Figure S36. HRMS of compound **8l**

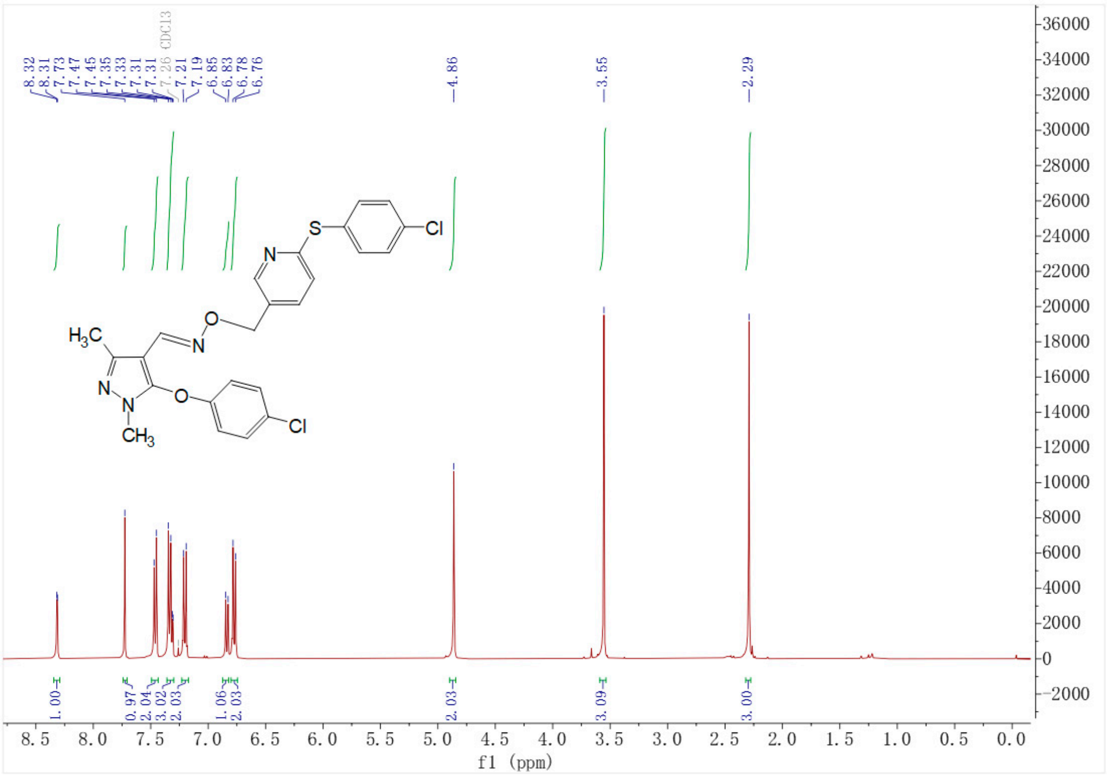

Figure S37. ^{1}H-NMR of compound **8m** (400 MHz, $CDCl_3$)

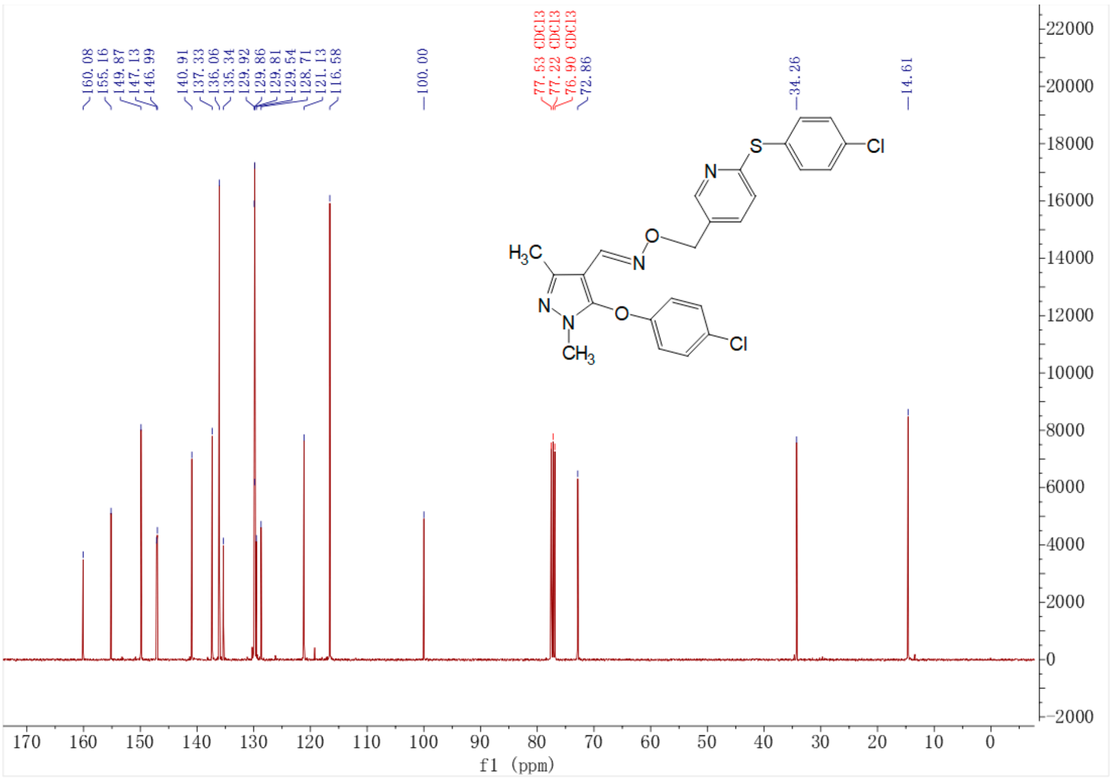

Figure S38. ^{13}C-NMR of compound **8m** (101 MHz, $CDCl_3$)

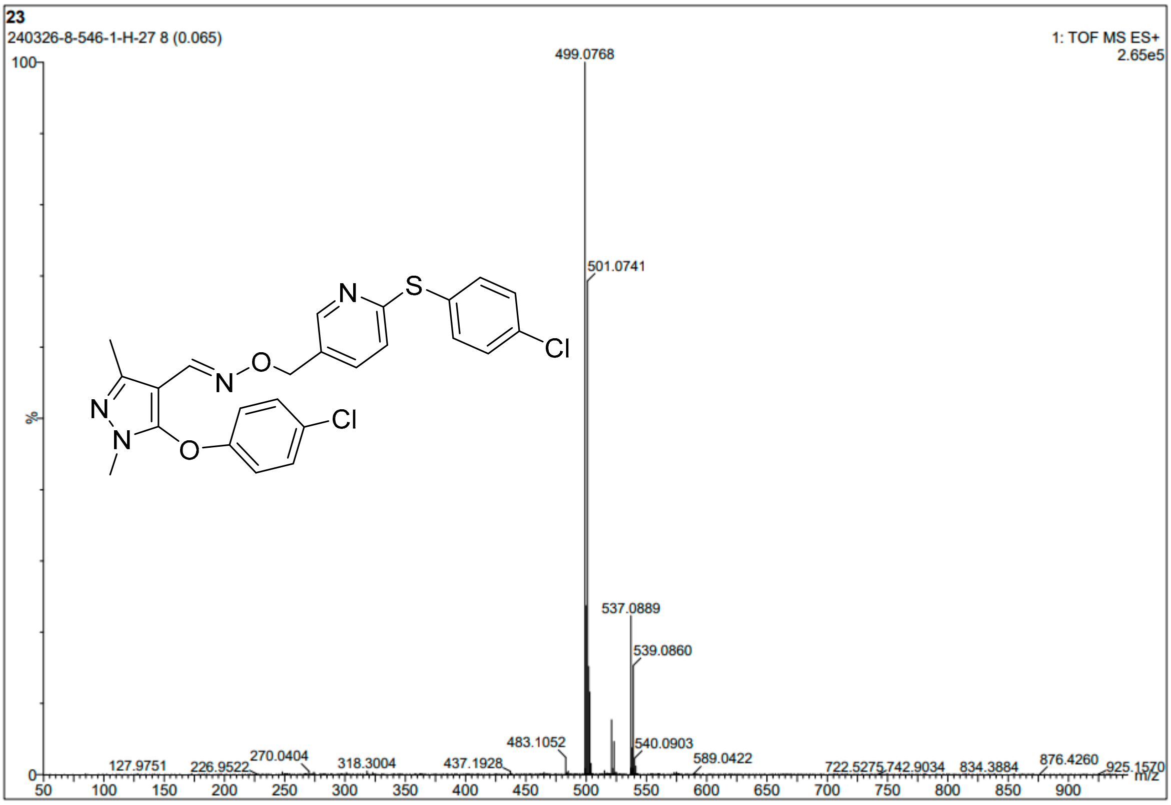

Figure S39. HRMS of compound **8m**

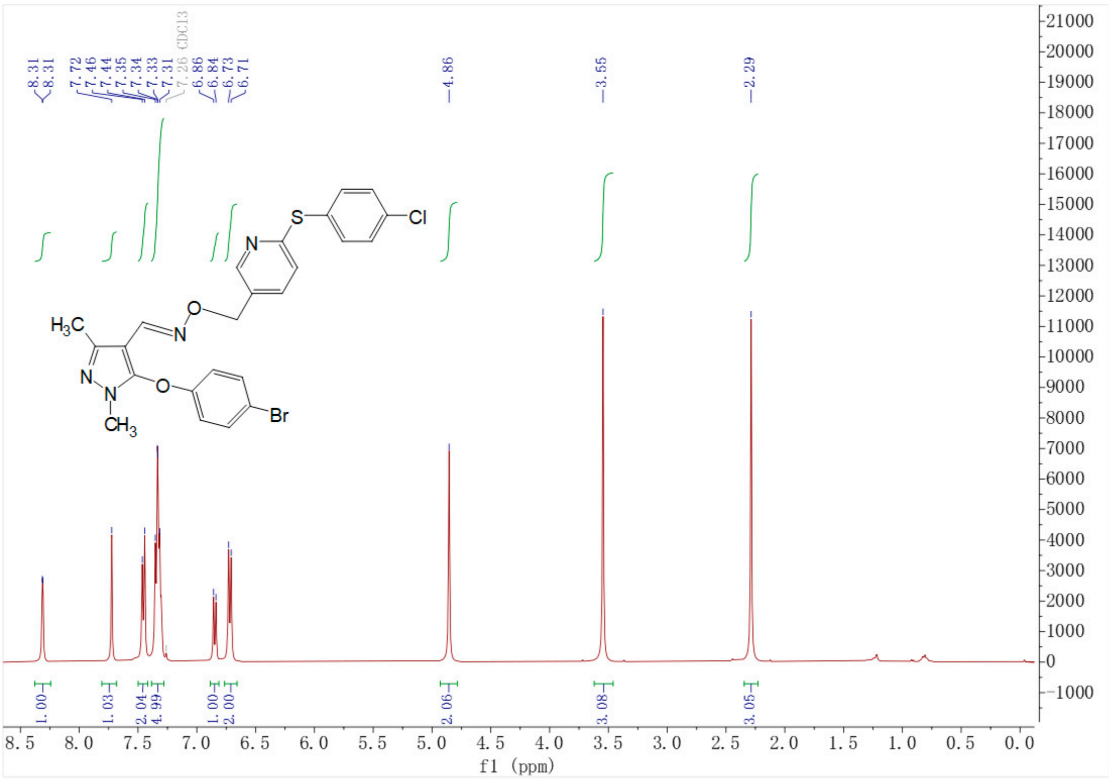

Figure S40. ^{1}H-NMR of compound **8n** (400 MHz, $CDCl_3$)

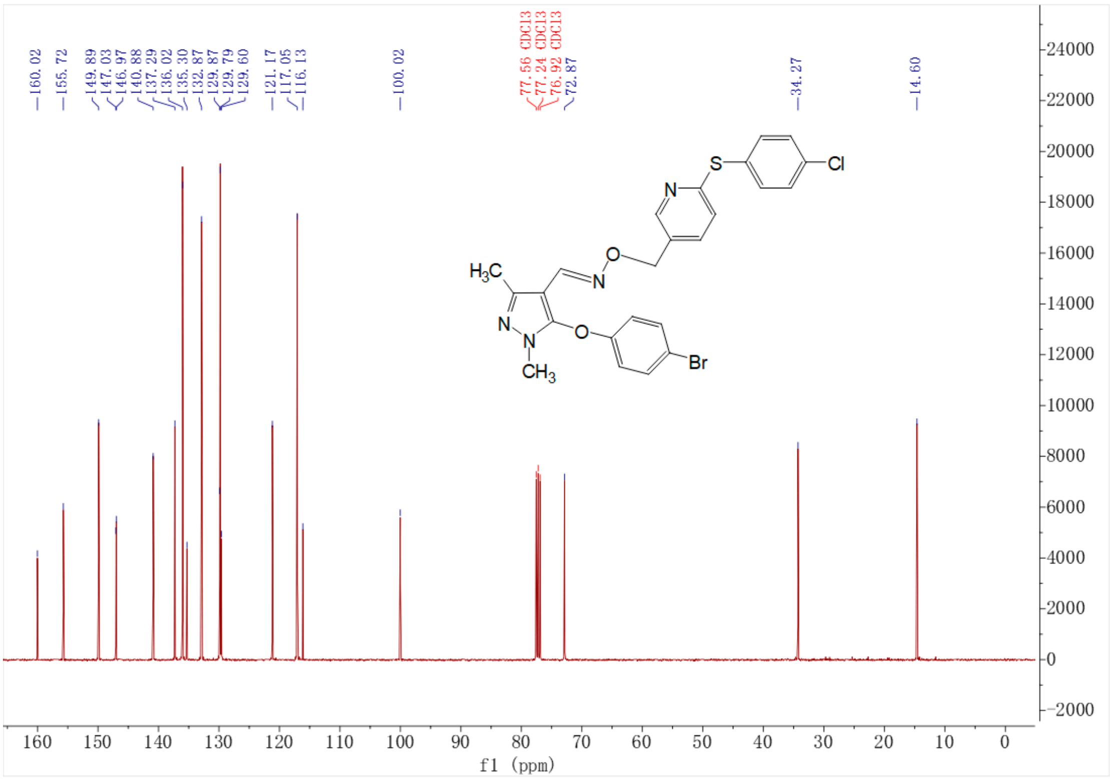

Figure S41. ^{13}C-NMR of compound **8n** (101 MHz, $CDCl_3$)

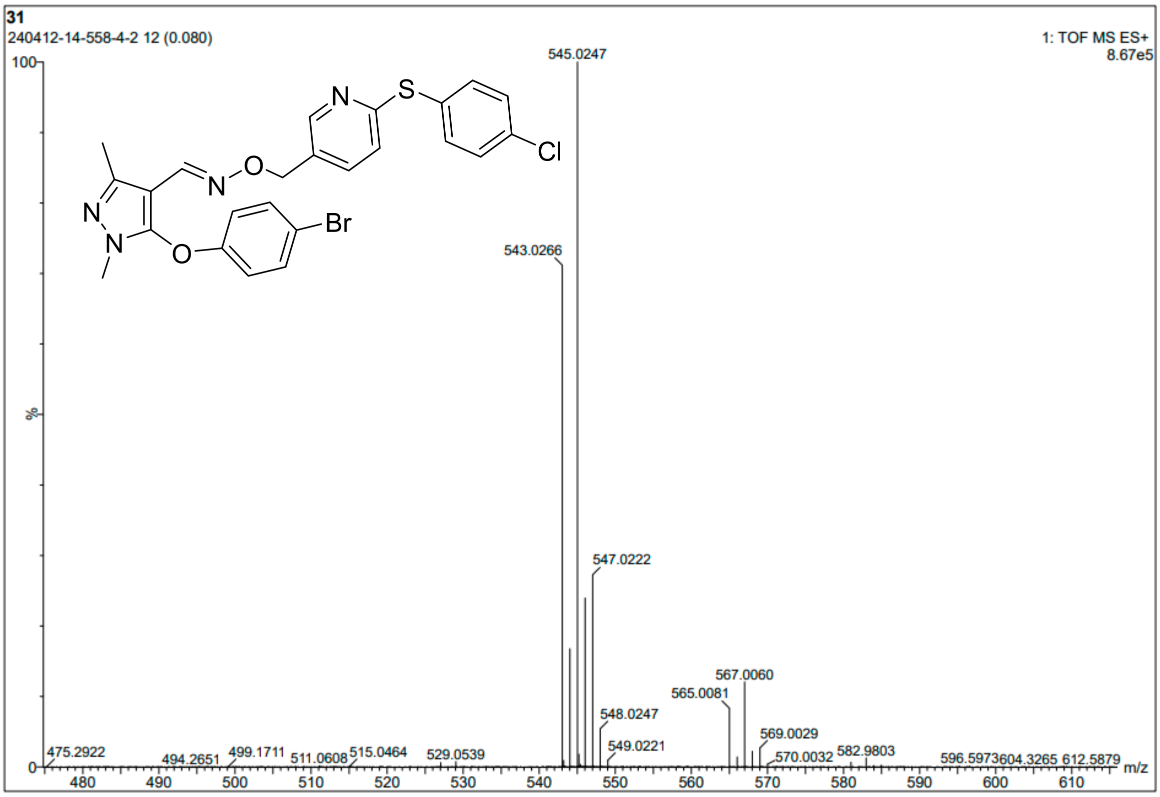

Figure S42. HRMS of compound **8n**

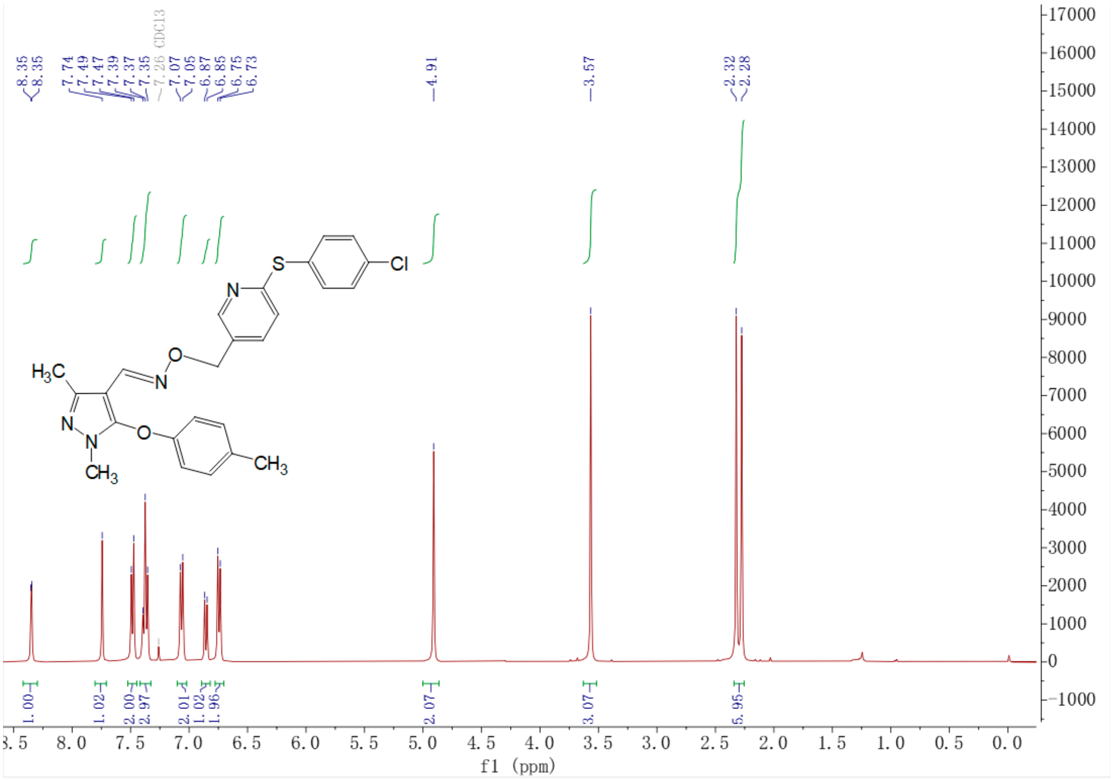

Figure S43. ^{1}H-NMR of compound **8o** (400 MHz, $CDCl_3$)

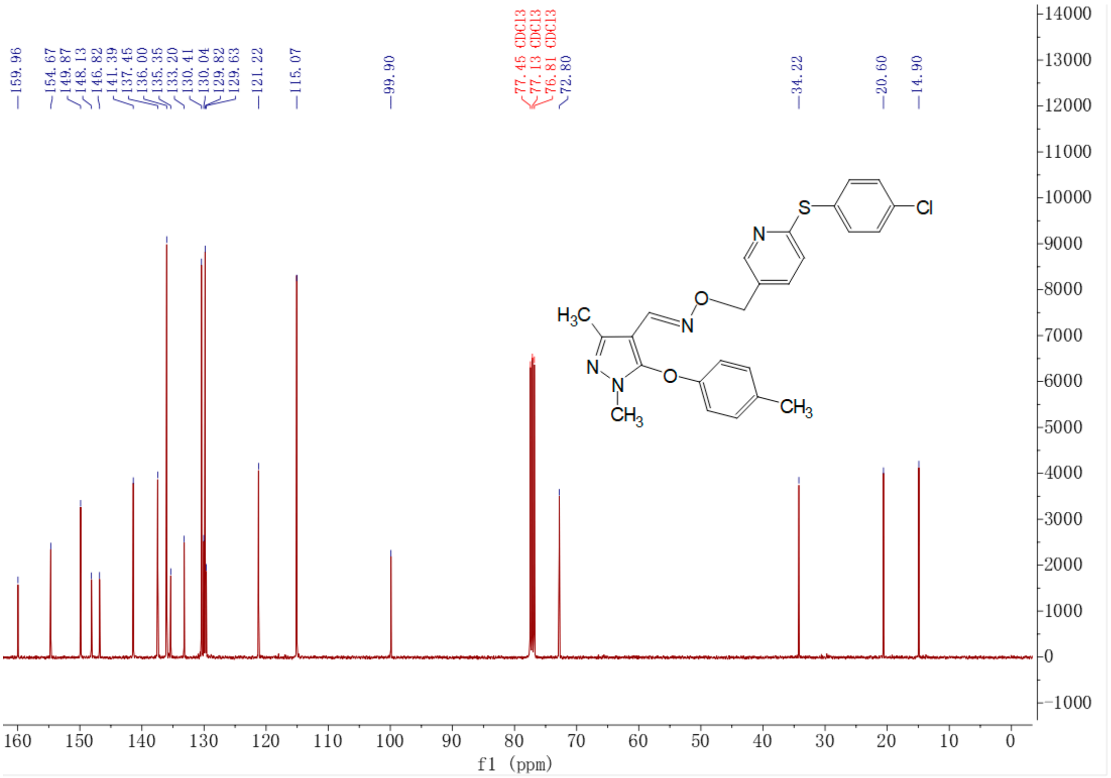

Figure S44. ^{13}C-NMR of compound **8o** (101 MHz, $CDCl_3$)

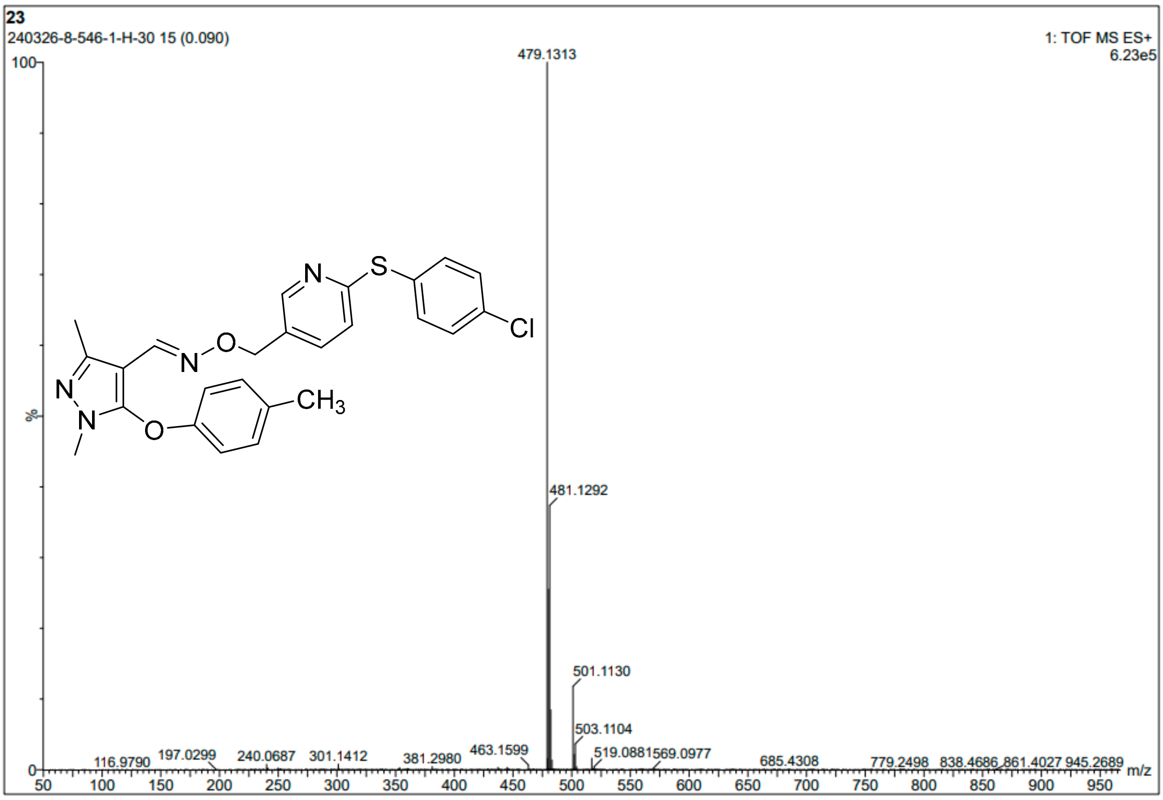

Figure S45. HRMS of compound **8p**

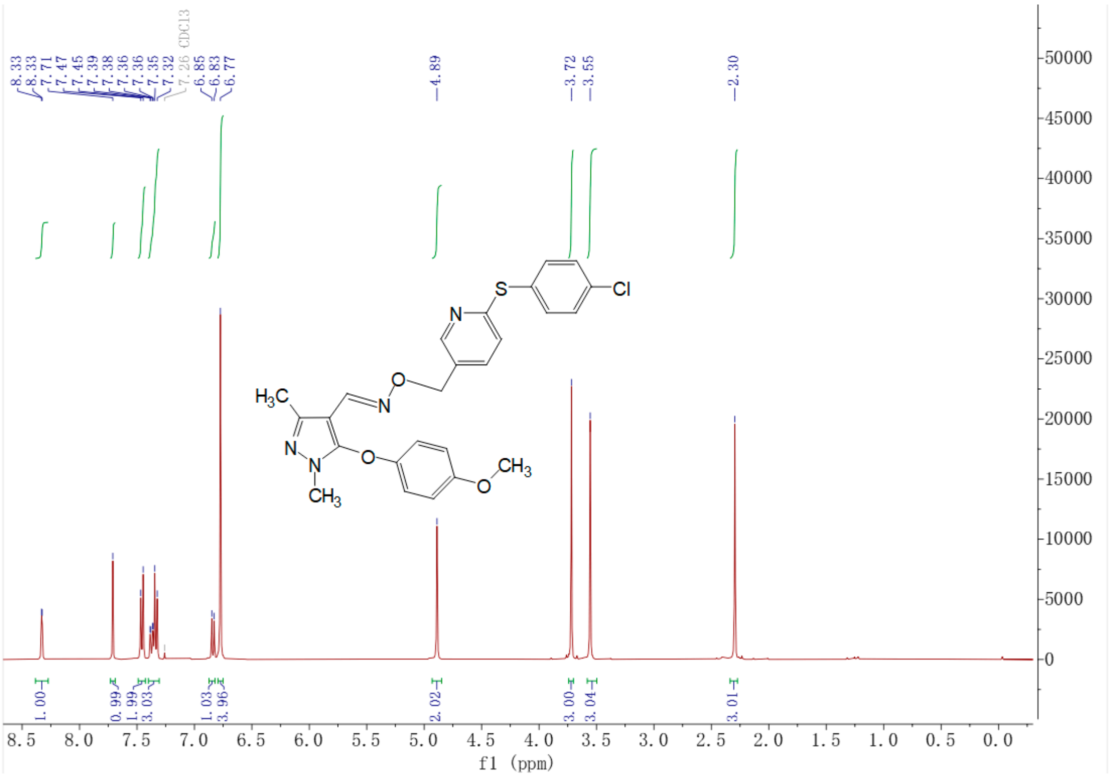

Figure S46. ^{1}H-NMR of compound **8p** (400 MHz, $CDCl_3$)

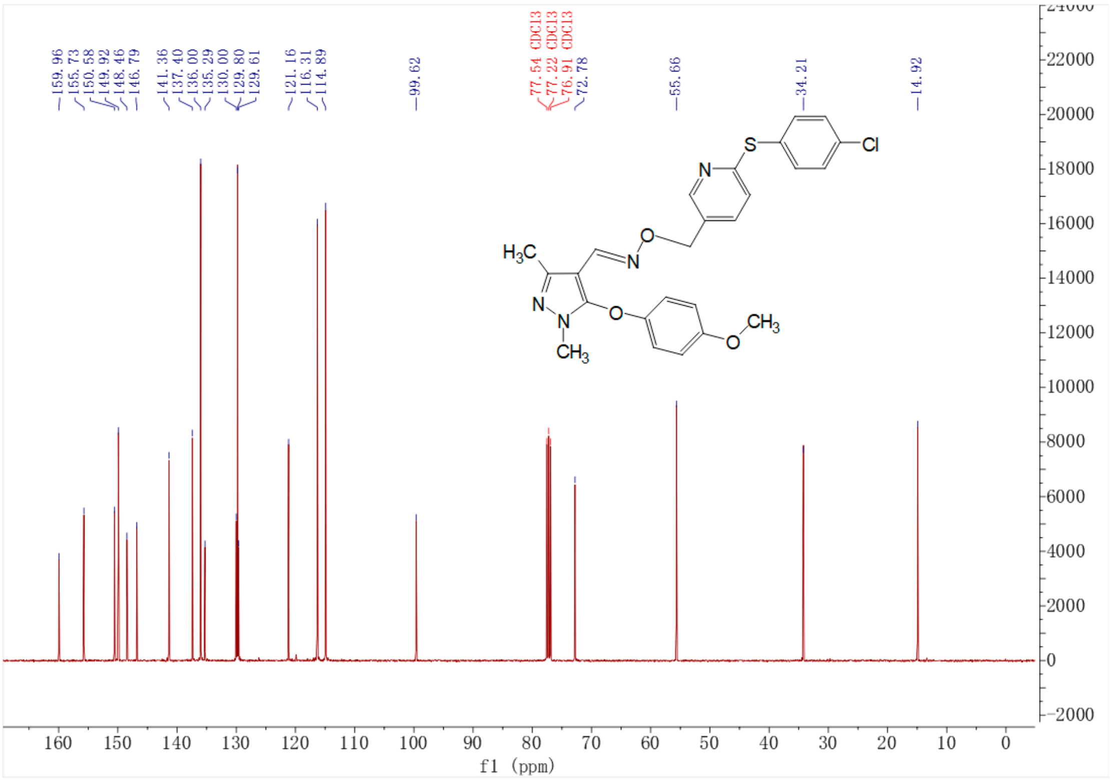

Figure S47. ^{13}C-NMR of compound **8p** (101 MHz, $CDCl_3$)

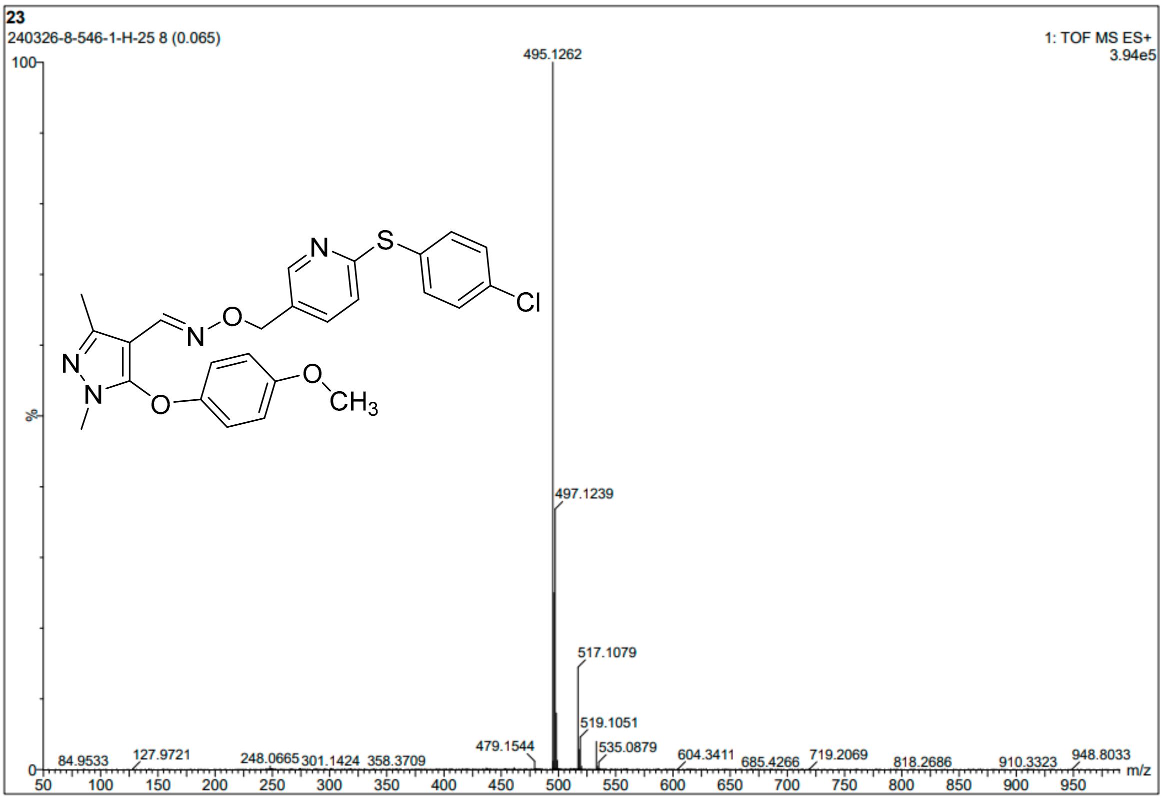

Figure S48. HRMS of compound **8p**

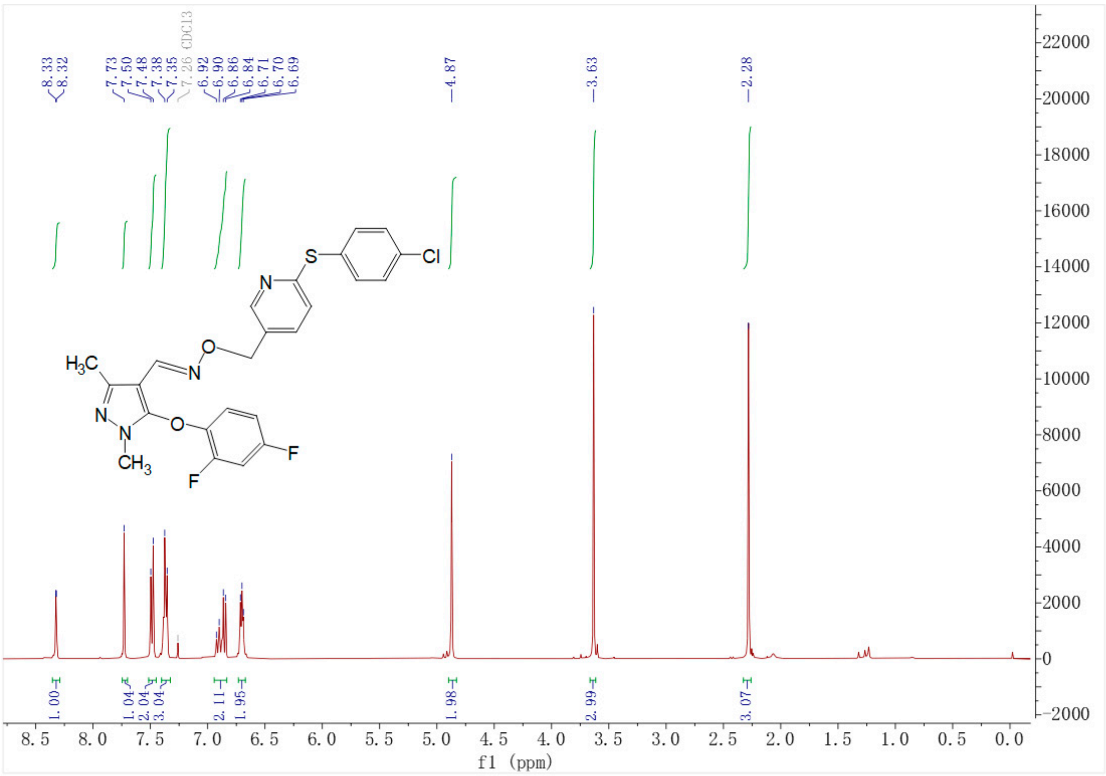

Figure S49. ¹H-NMR of compound **8q** (400 MHz, $CDCl_3$)

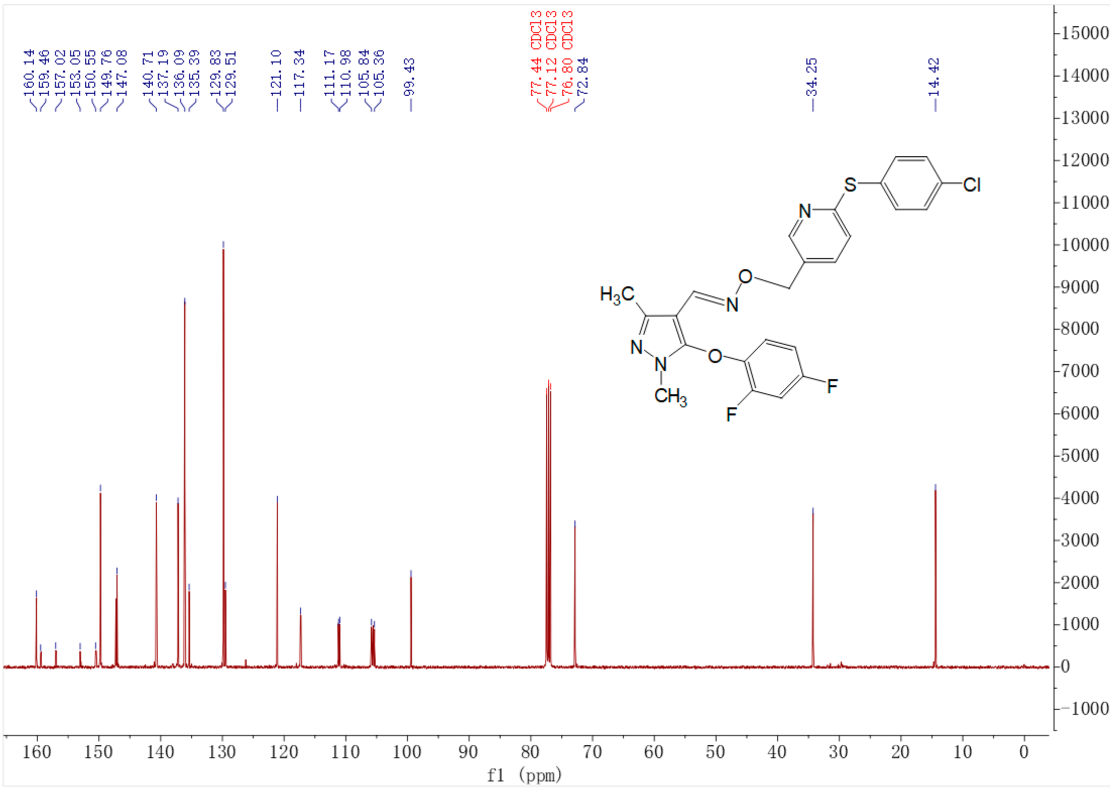

Figure S50. ¹³C-NMR of compound **8q** (101 MHz, $CDCl_3$)

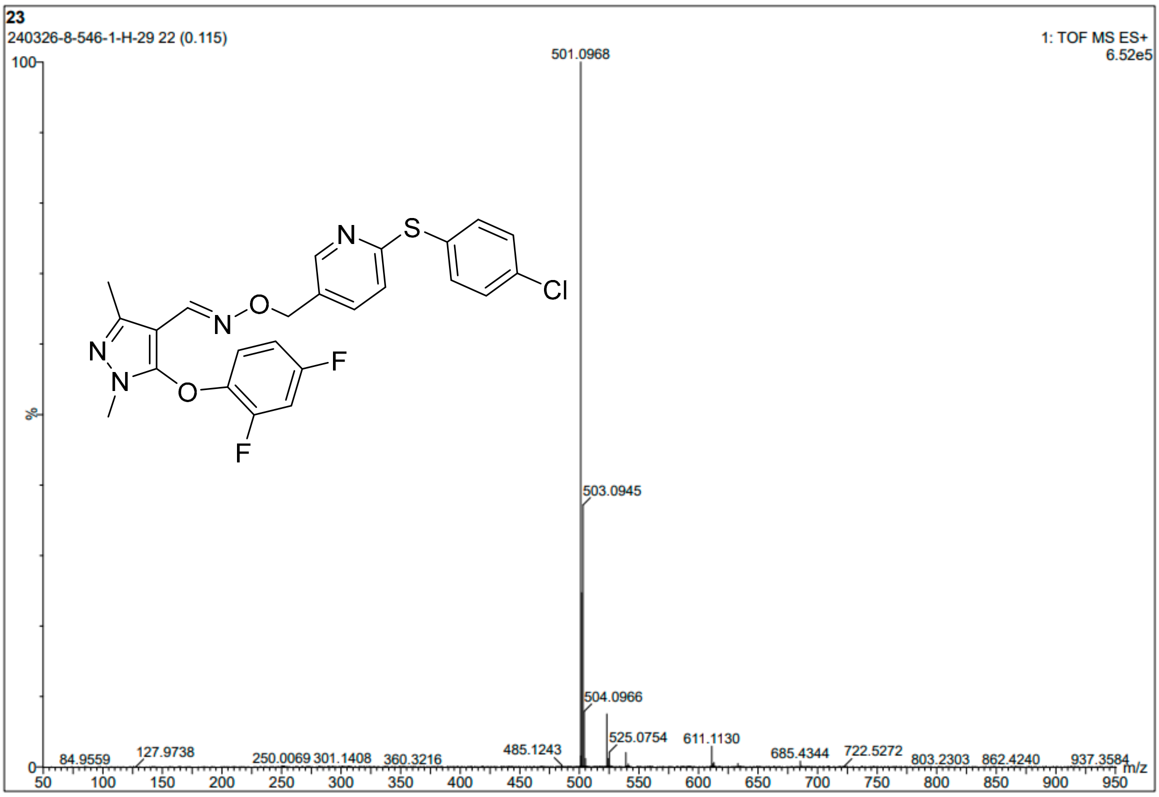

Figure S51. HRMS of compound **8q**

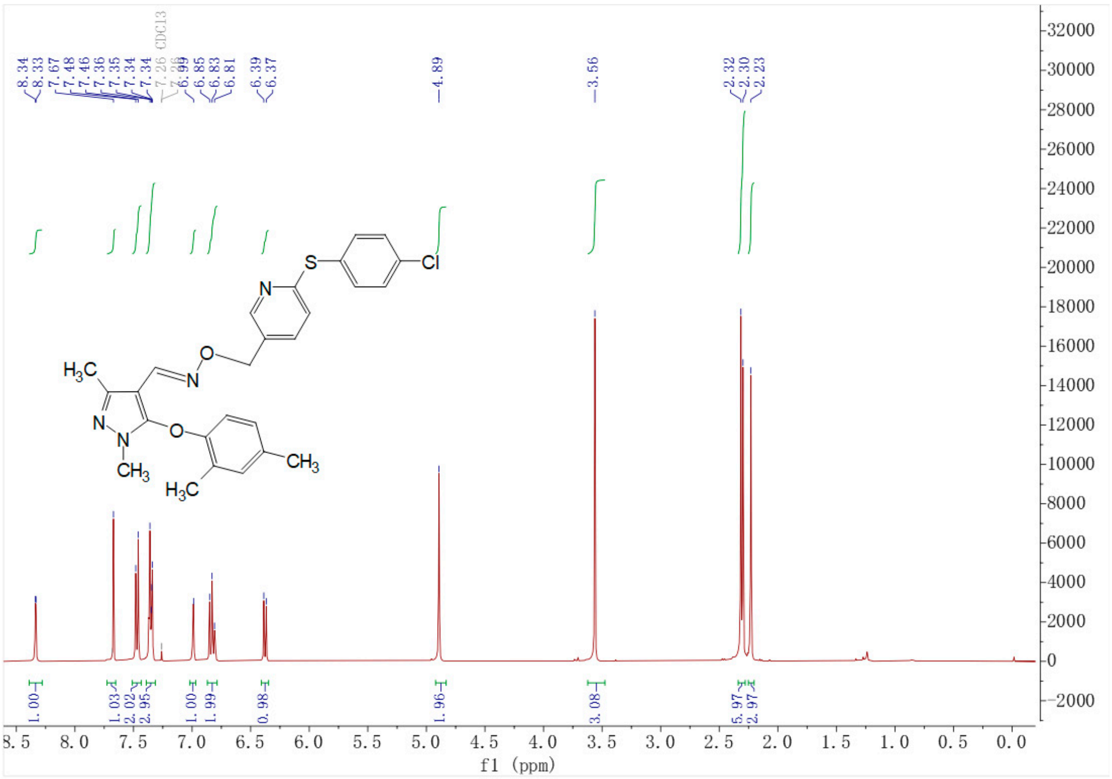

Figure S52. ^{1}H-NMR of compound **8r** (400 MHz, $CDCl_3$)

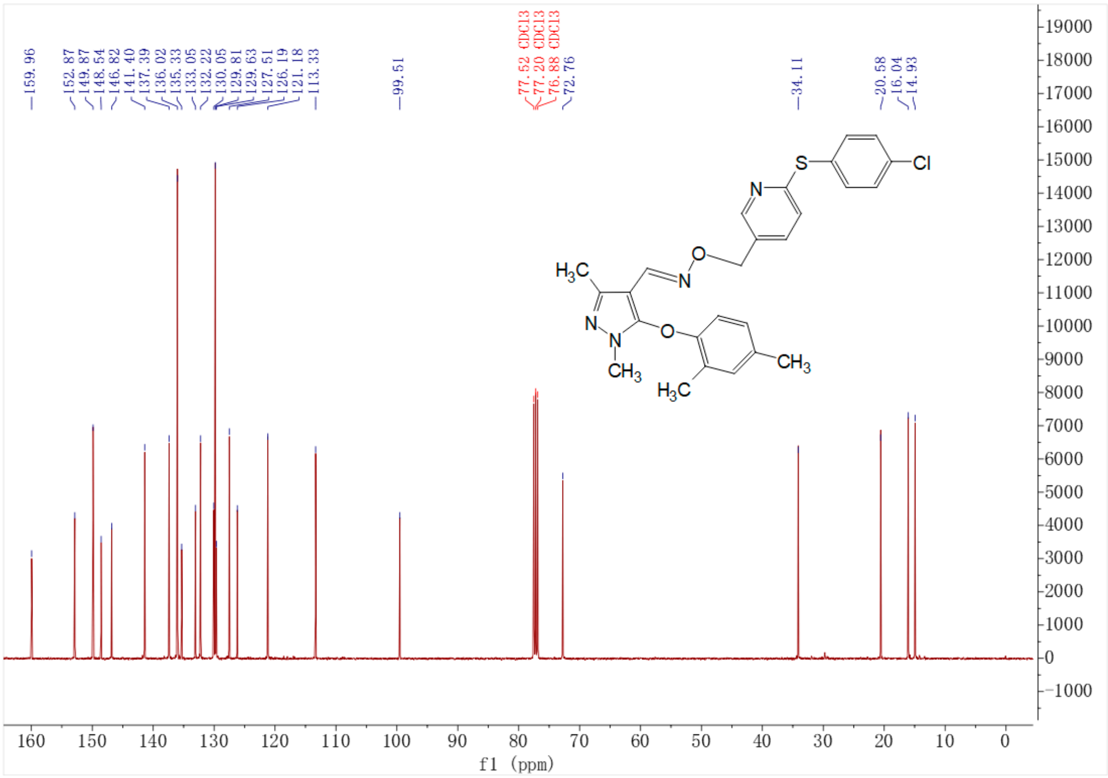

Figure S53. ^{13}C-NMR of compound **8r** (101 MHz, $CDCl_3$)

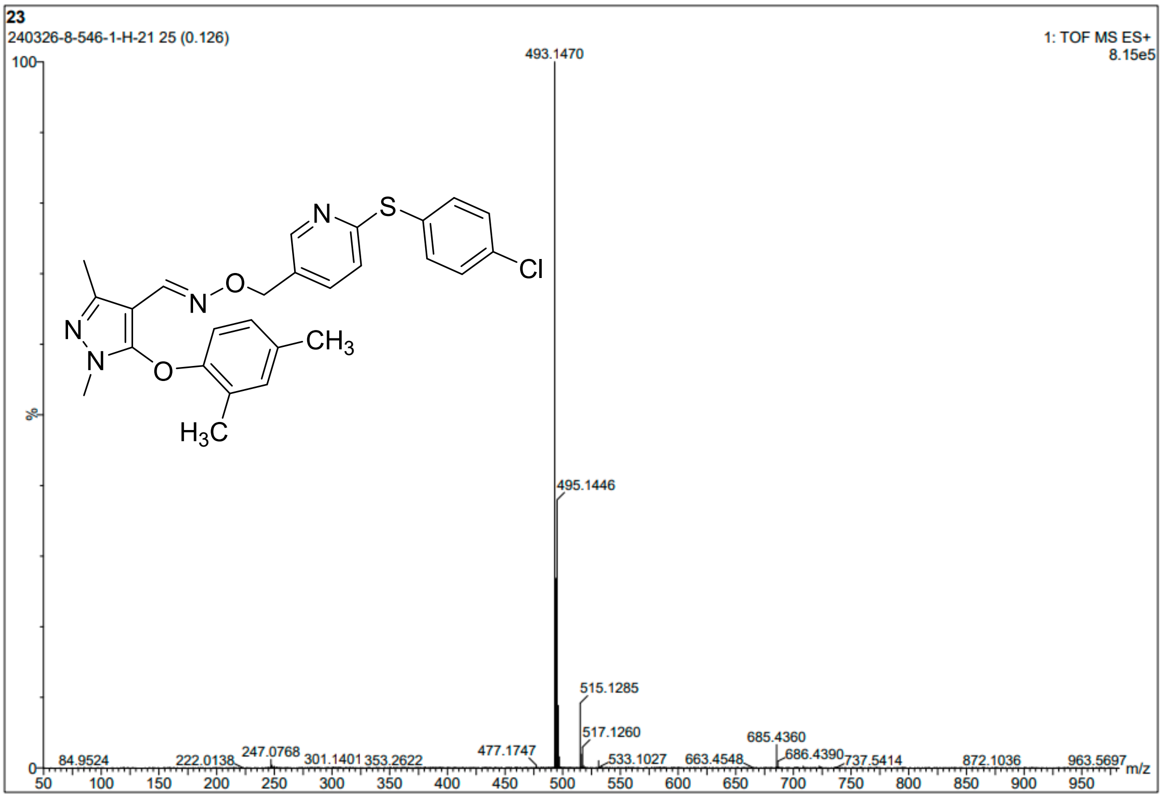

Figure S54. HRMS of compound **8r**

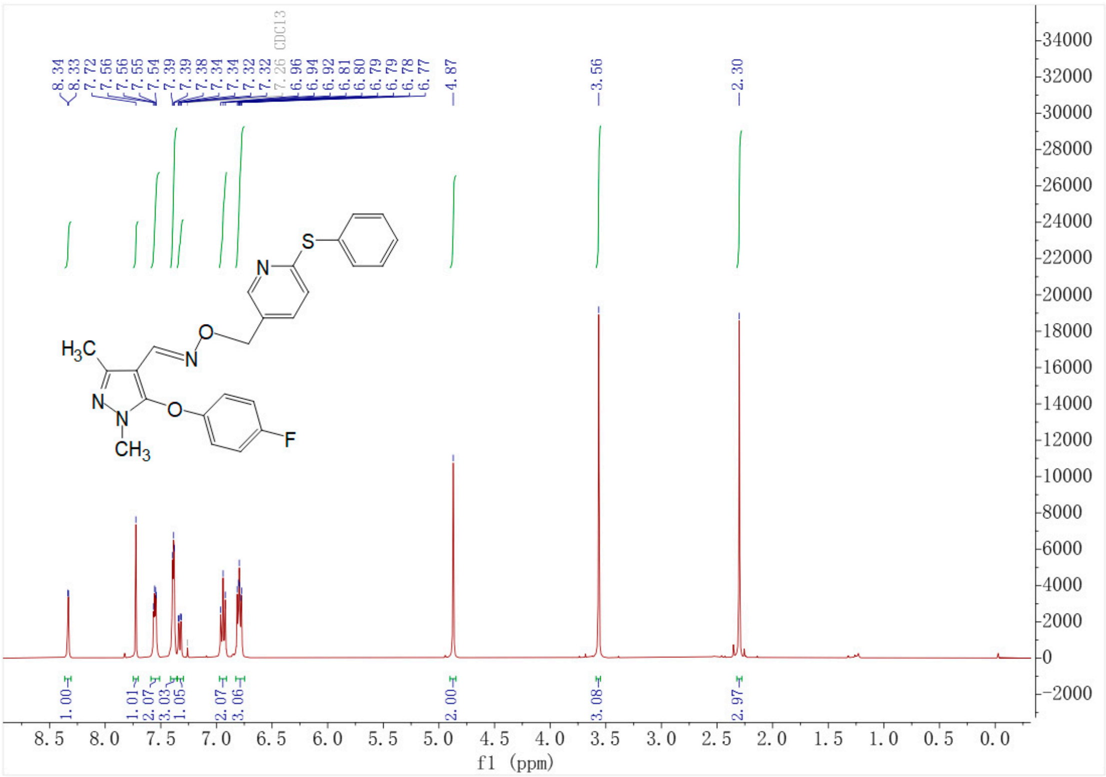

Figure S55. ^{1}H-NMR of compound **8s** (400 MHz, $CDCl_3$)

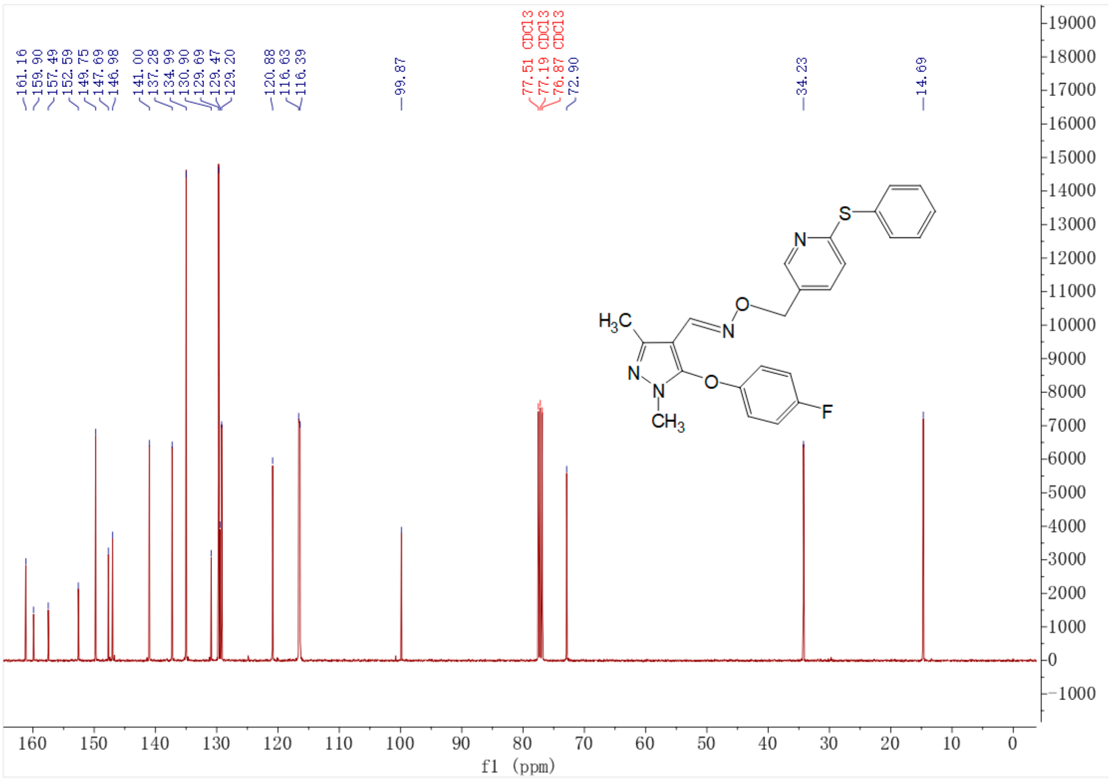

Figure S56. ^{13}C-NMR of compound **8s** (101 MHz, $CDCl_3$)

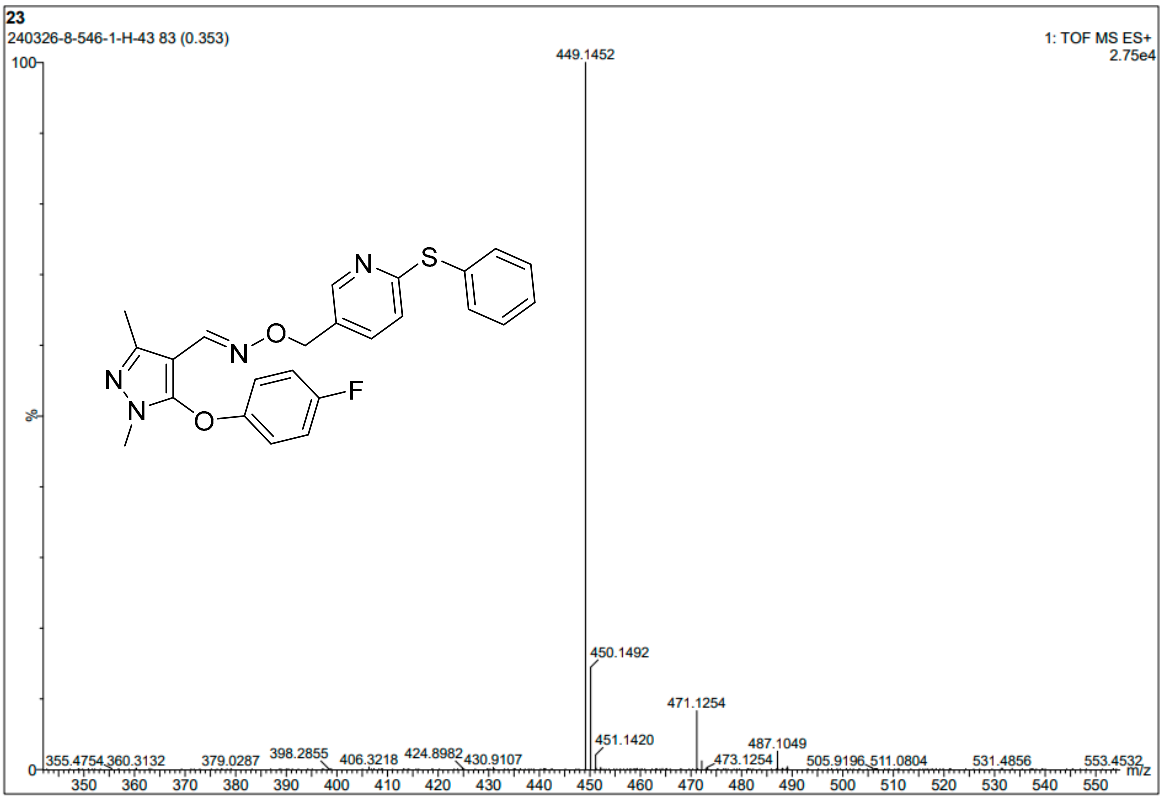

Figure S57. HRMS of compound **8s**

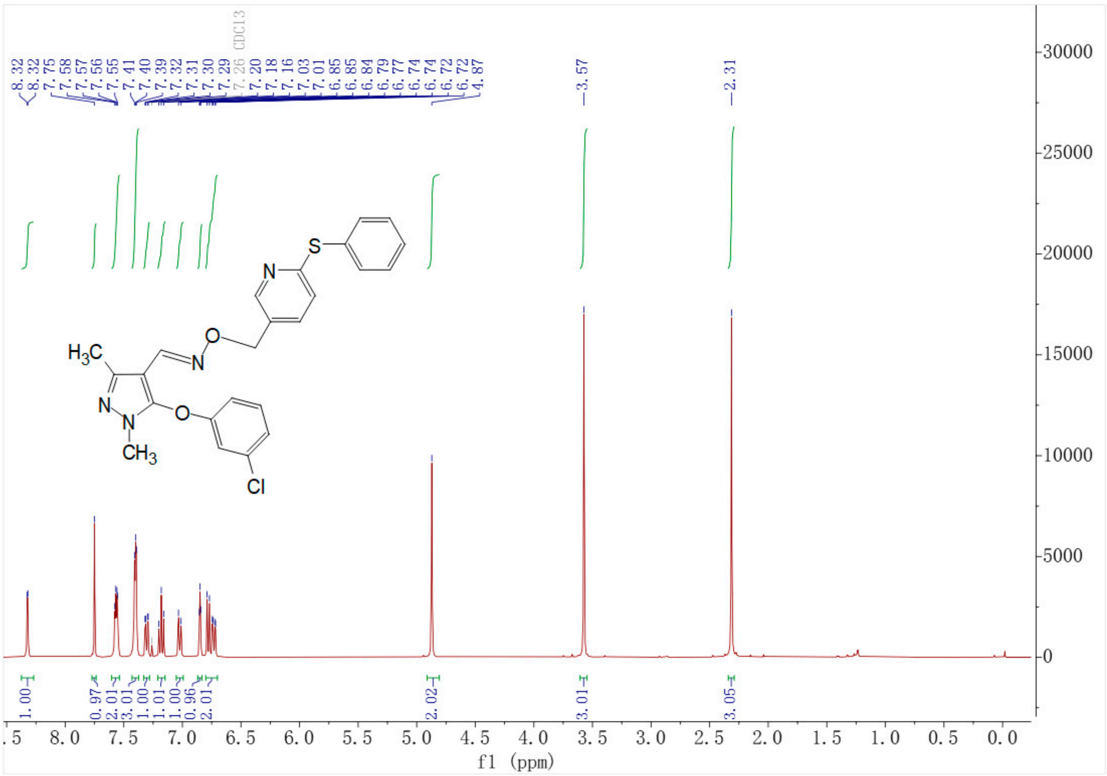

Figure S58. ^{1}H-NMR of compound **8t** (400 MHz, $CDCl_3$)

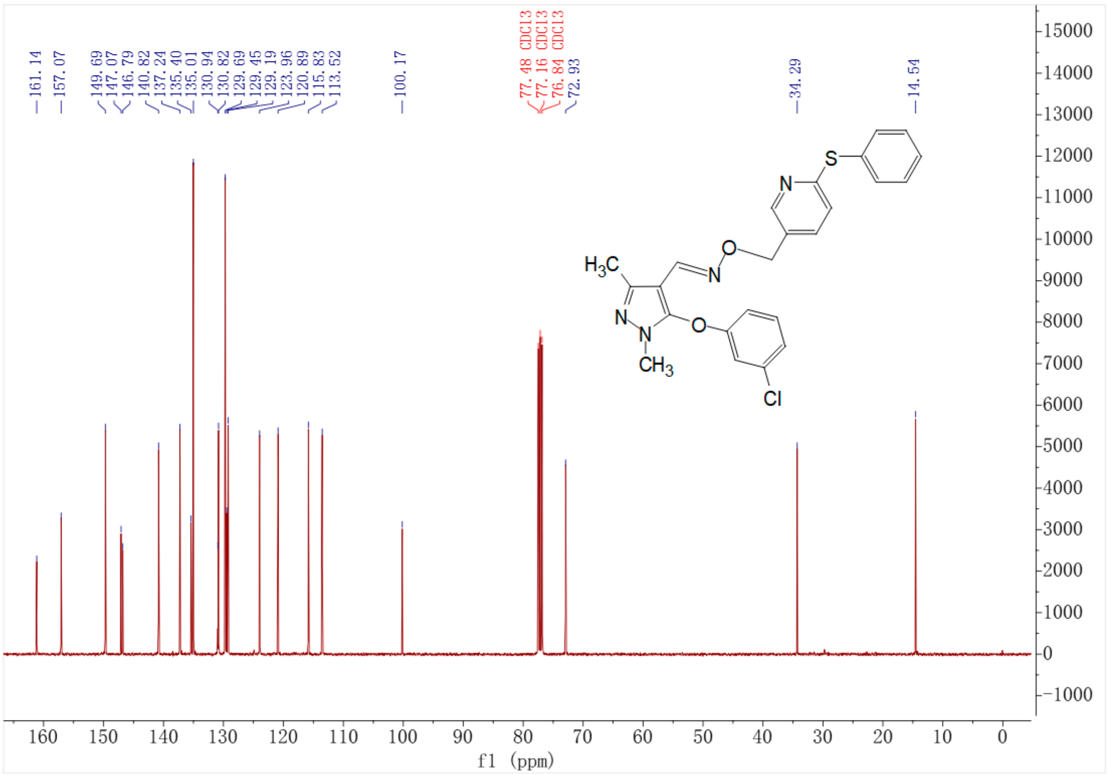

Figure S59. ^{13}C-NMR of compound **8t** (101 MHz, $CDCl_3$)

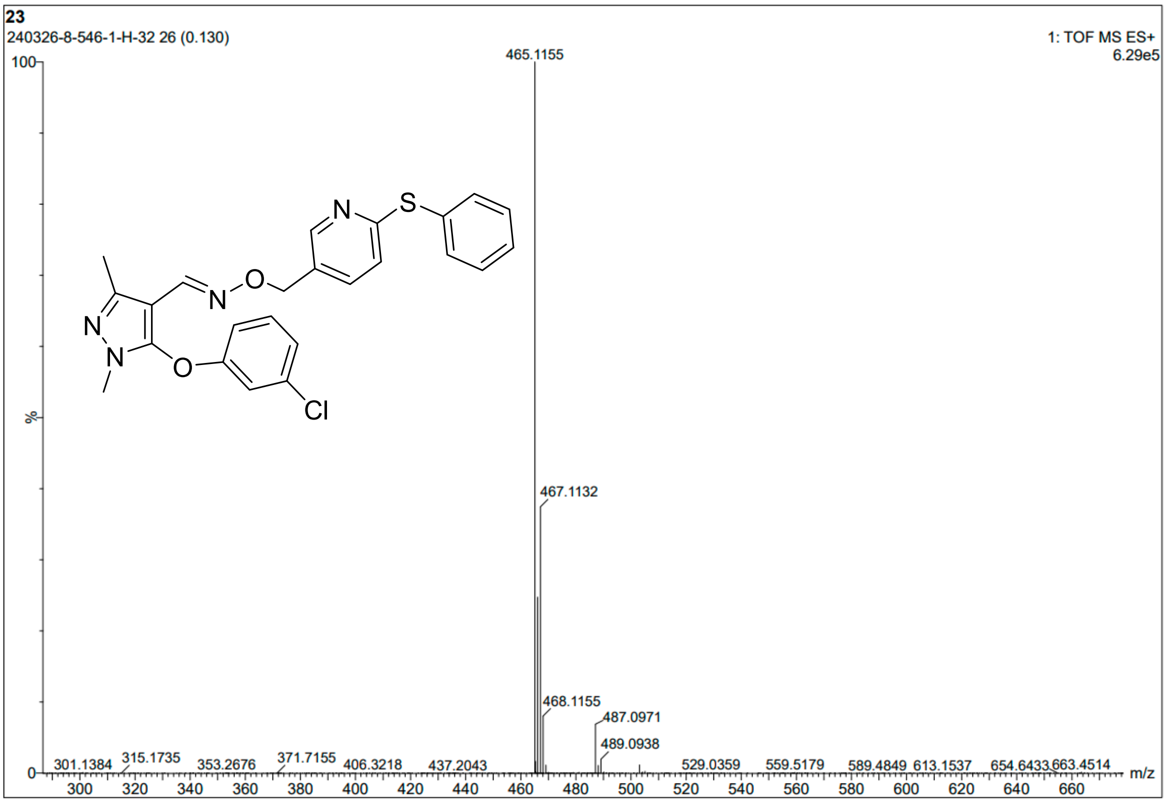

Figure S60. HRMS of compound **8t**

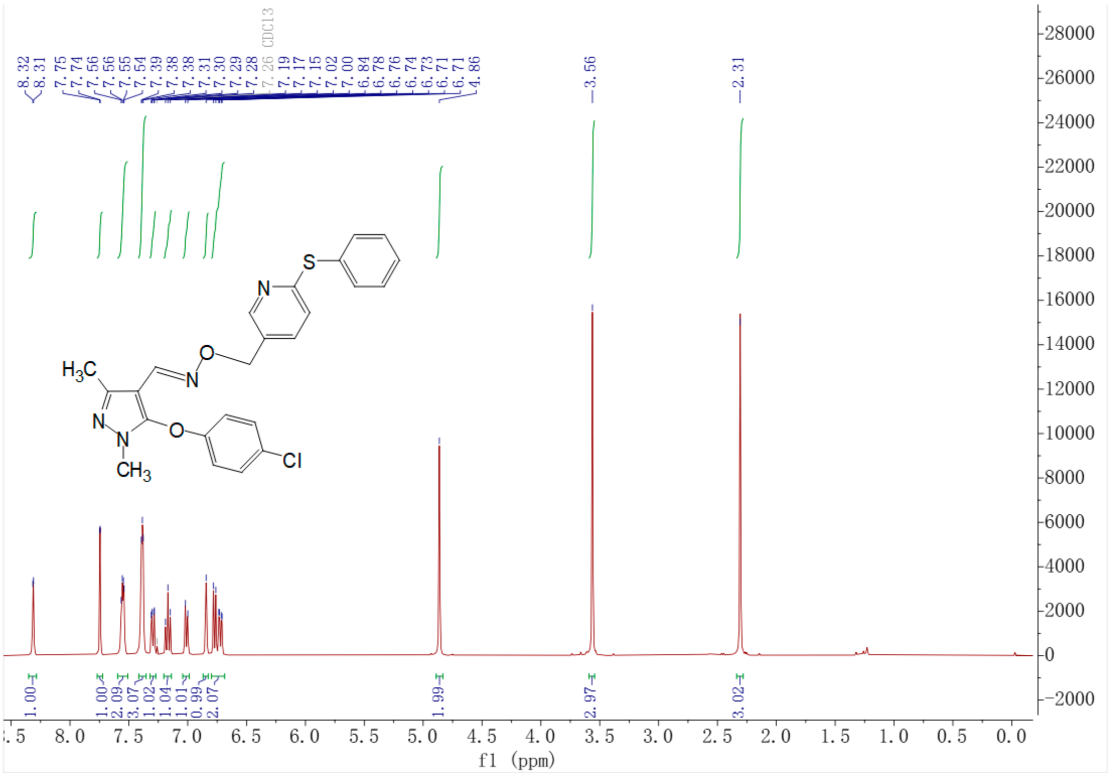

Figure S61. ^{1}H-NMR of compound **8u** (400 MHz, $CDCl_3$)

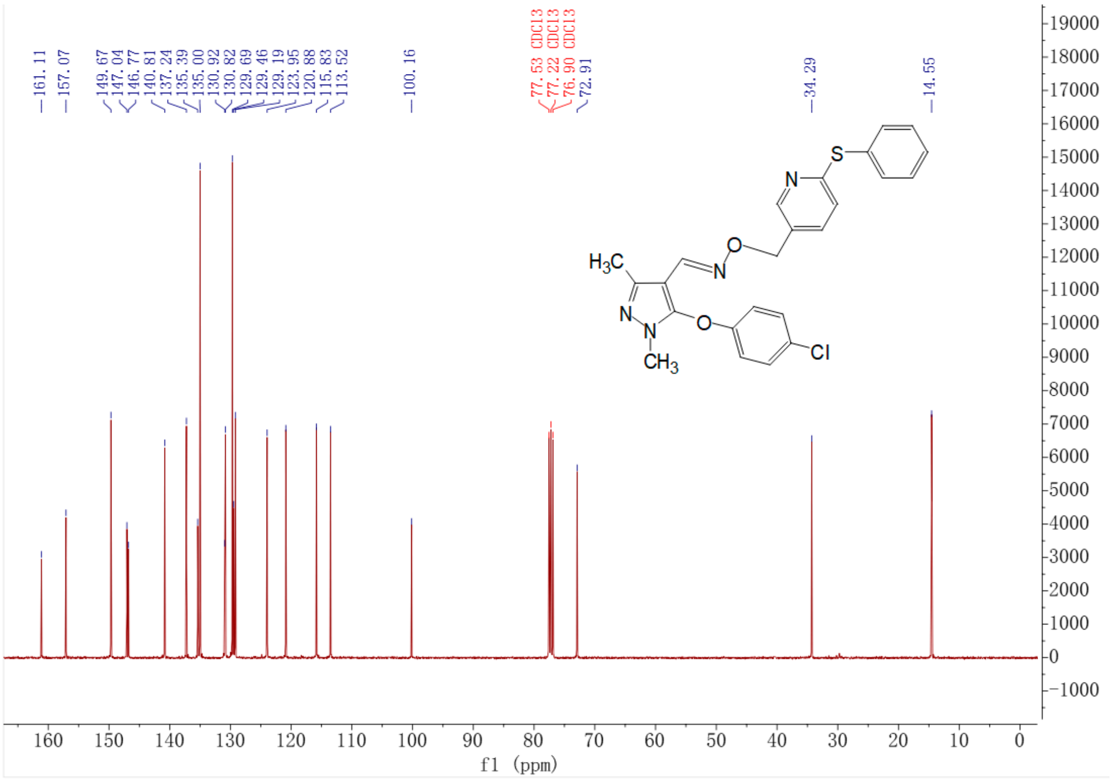

Figure S62. ^{13}C-NMR of compound **8u** (101 MHz, $CDCl_3$)

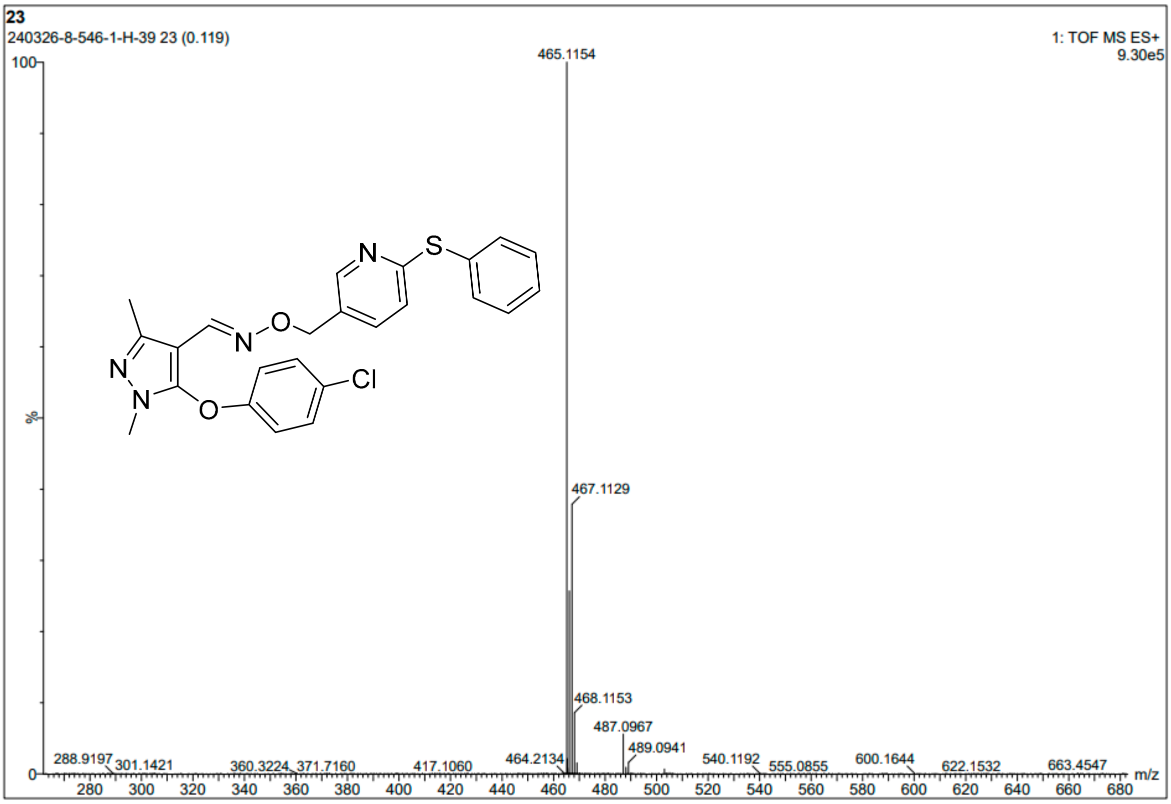

Figure S63. HRMS of compound **8u**

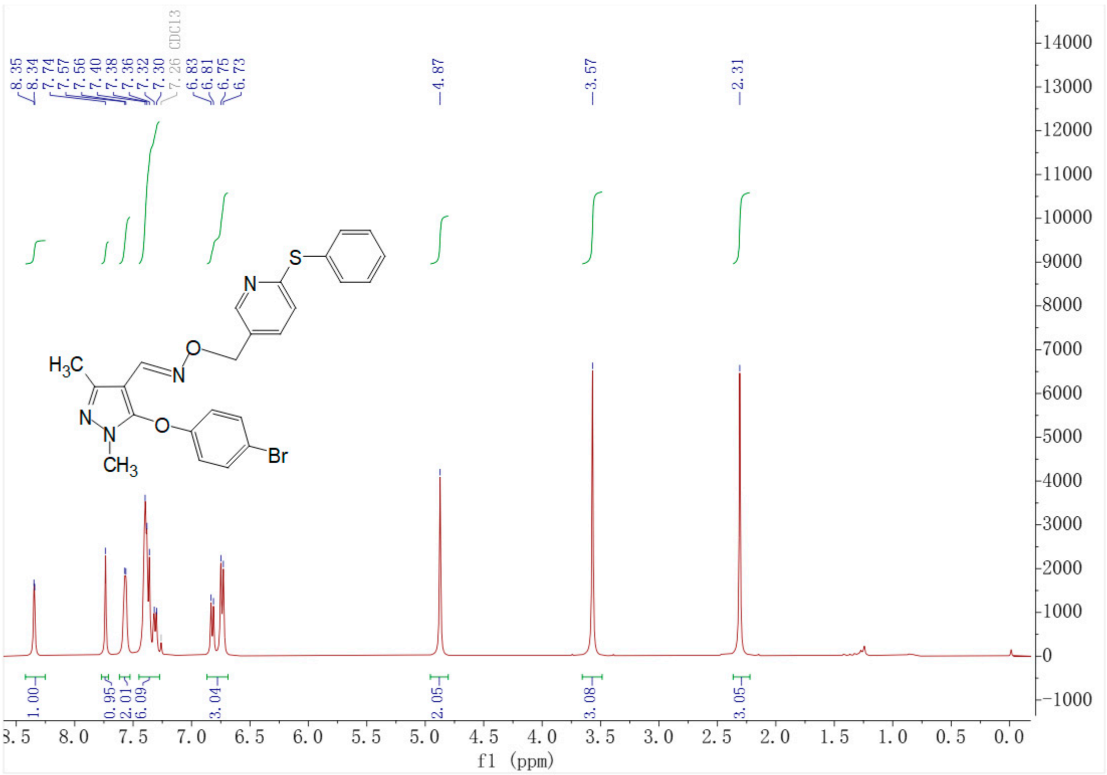

Figure S64. ^{1}H-NMR of compound **8v** (400 MHz, $CDCl_3$)

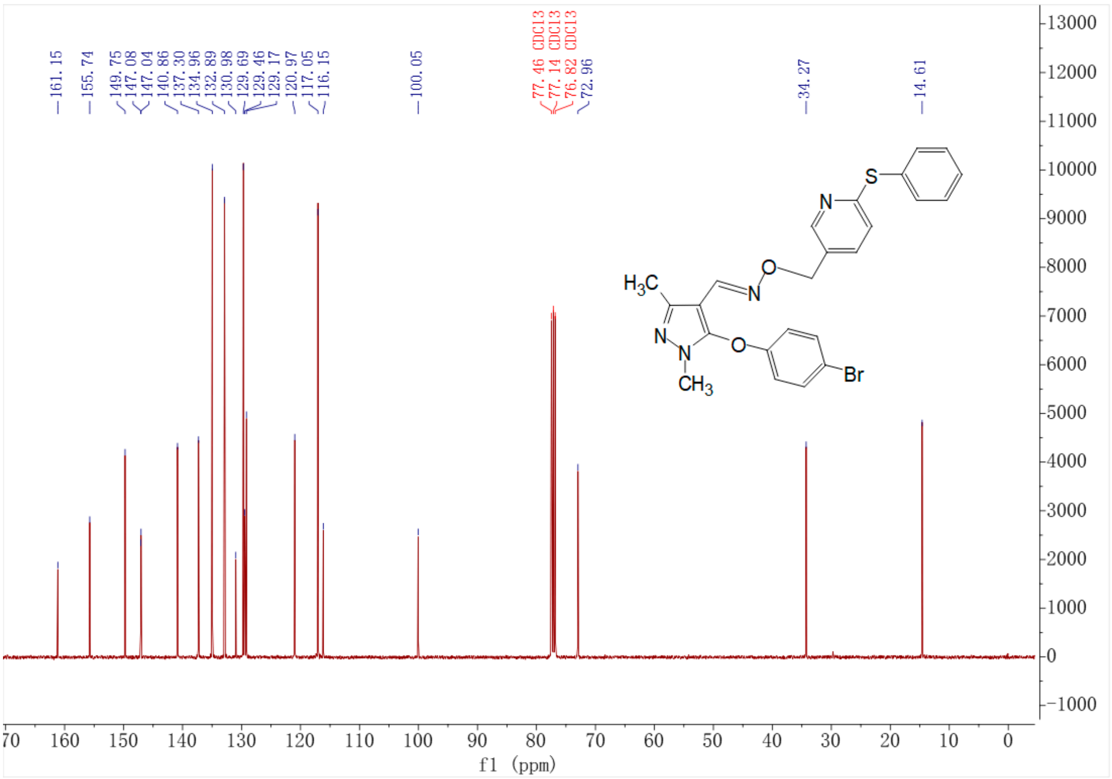

Figure S65. ^{13}C-NMR of compound **8v** (101 MHz, $CDCl_3$)

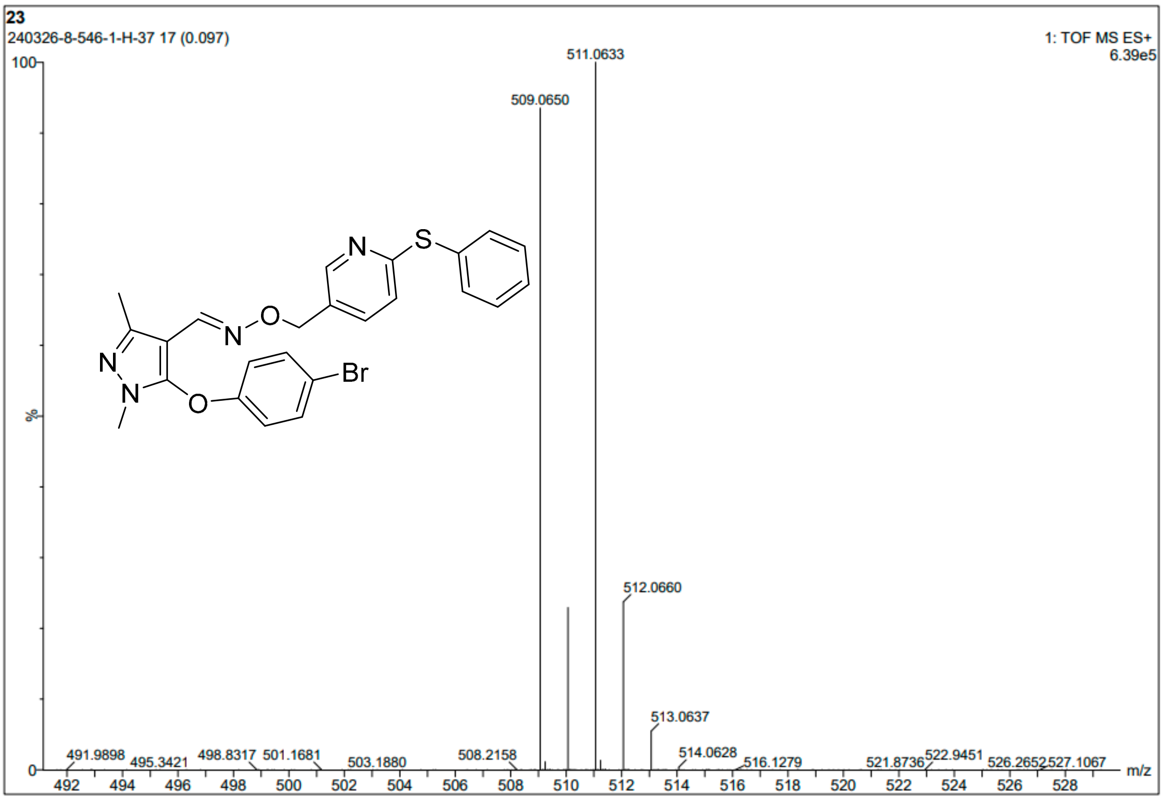

Figure S66. HRMS of compound **8v**

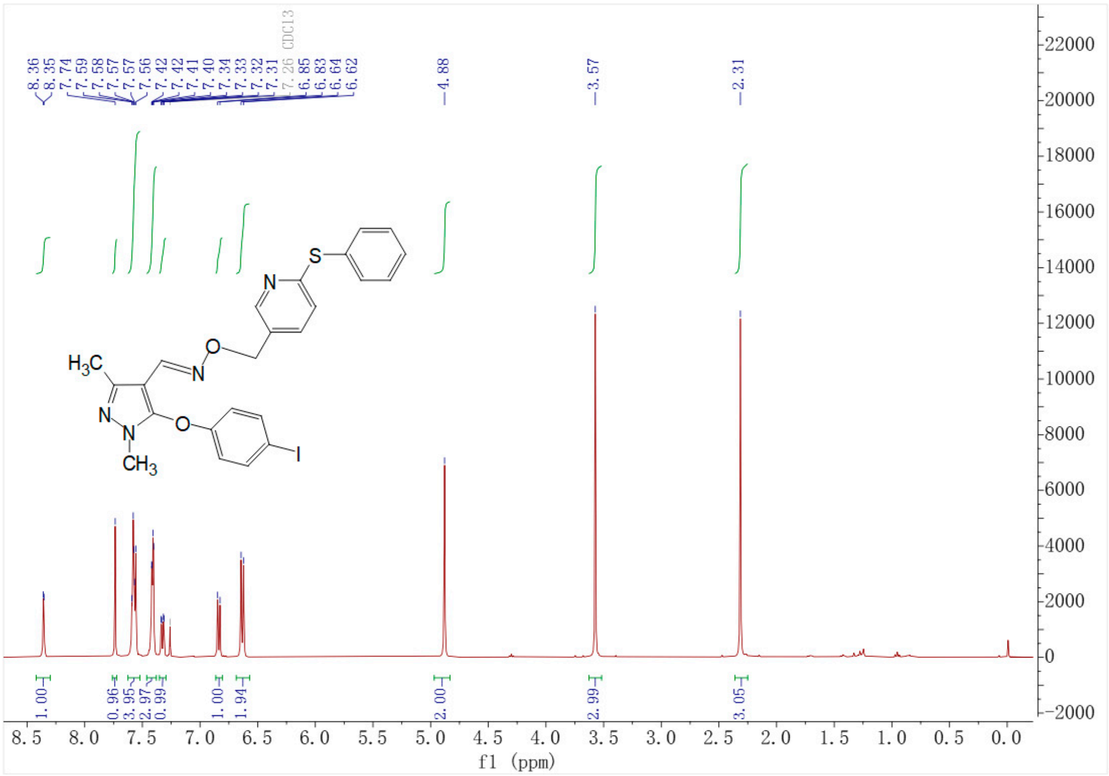

Figure S67. ^{1}H-NMR of compound **8w** (400 MHz, $CDCl_3$)

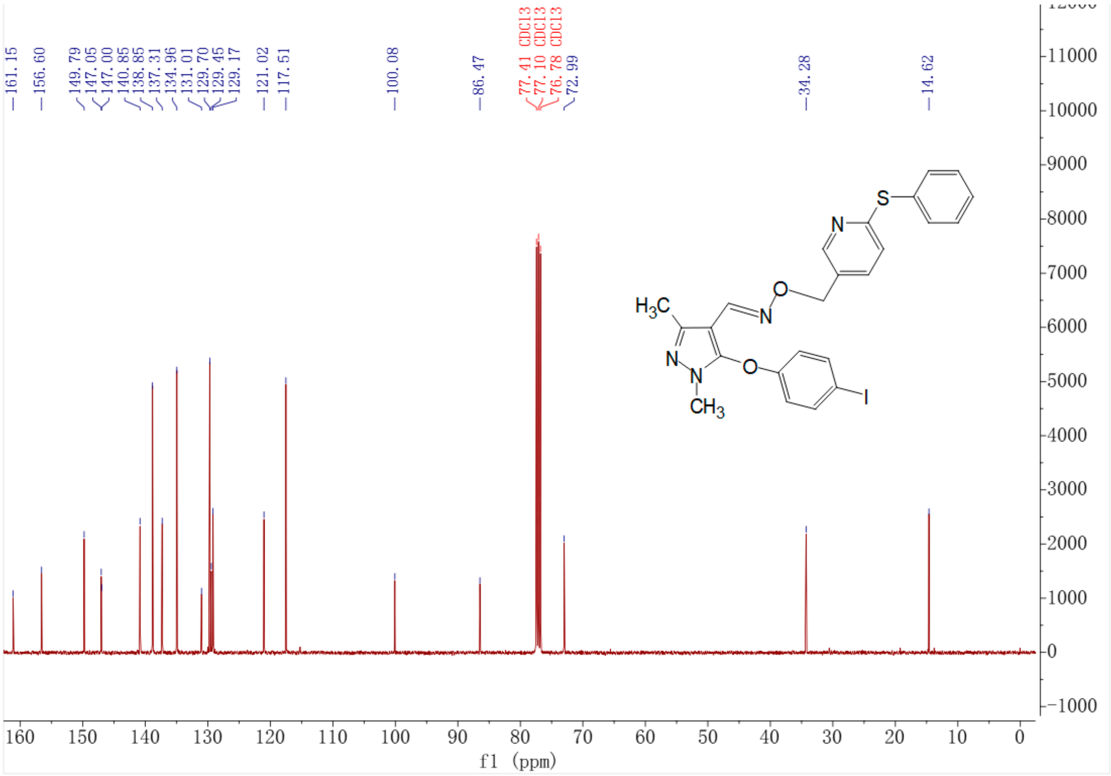

Figure S68. ^{13}C-NMR of compound **8w** (101 MHz, $CDCl_3$)

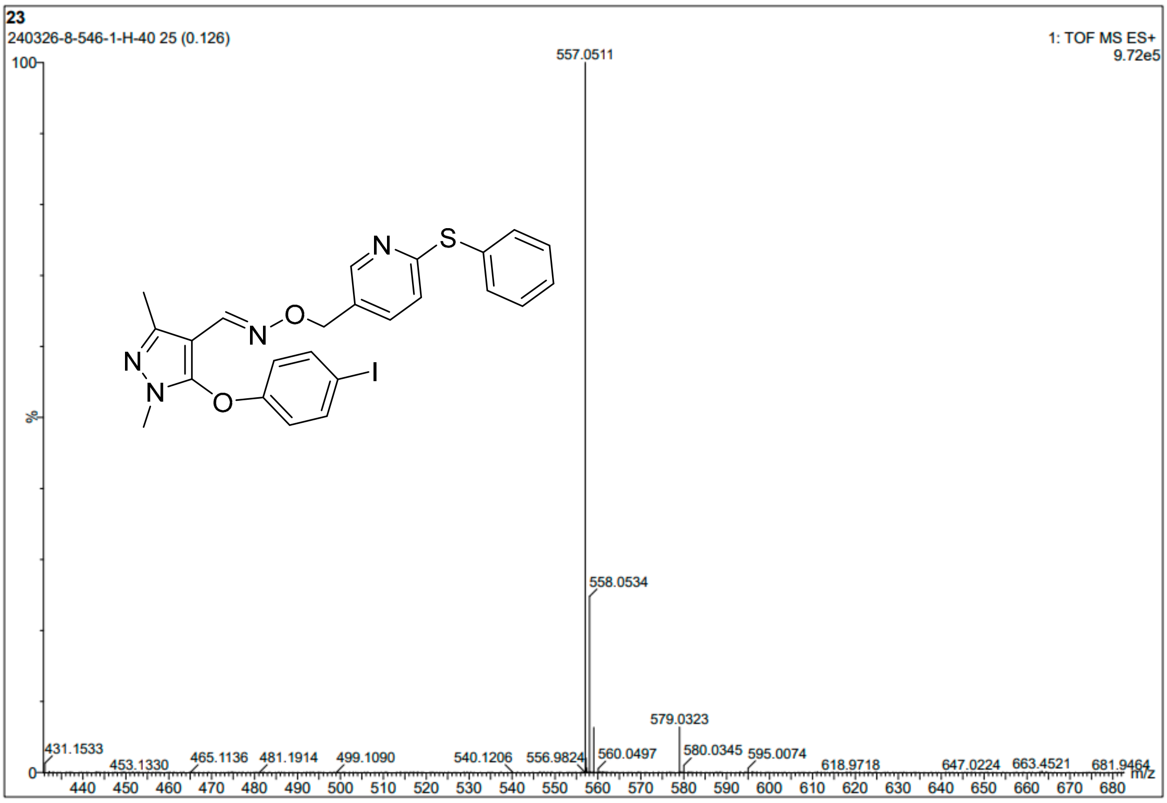

Figure S69. HRMS of compound **8w**

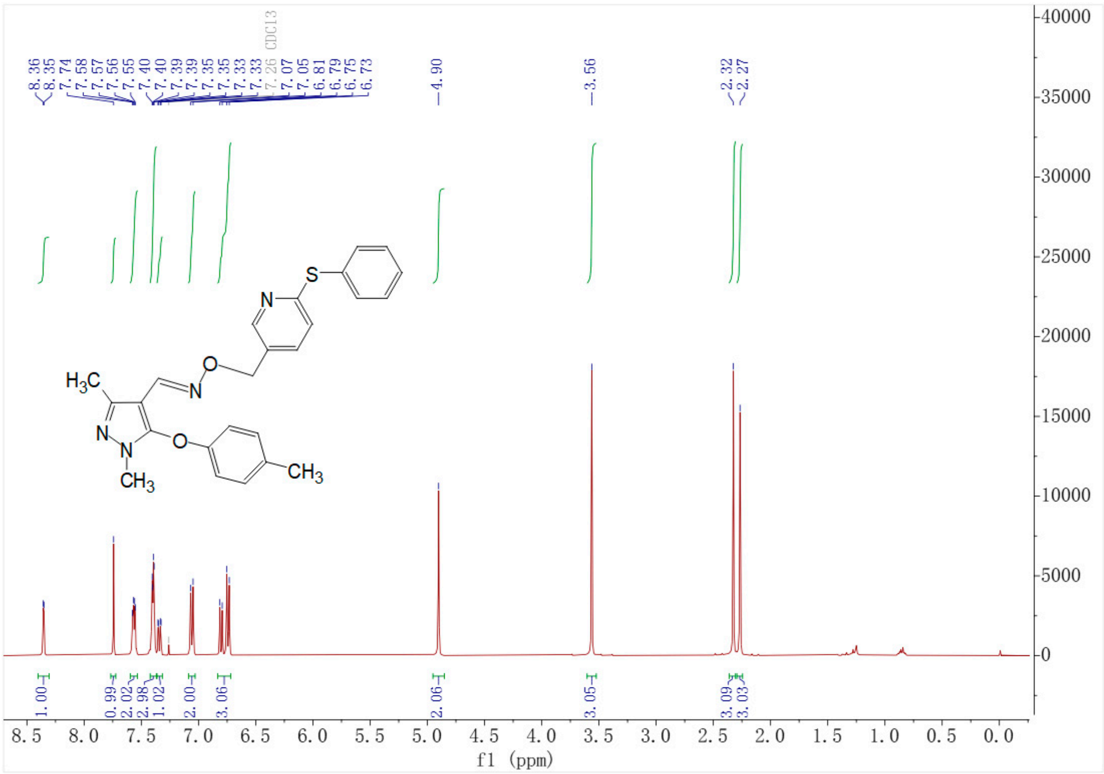

Figure S70. ^{1}H-NMR of compound **8x** (400 MHz, $CDCl_3$)

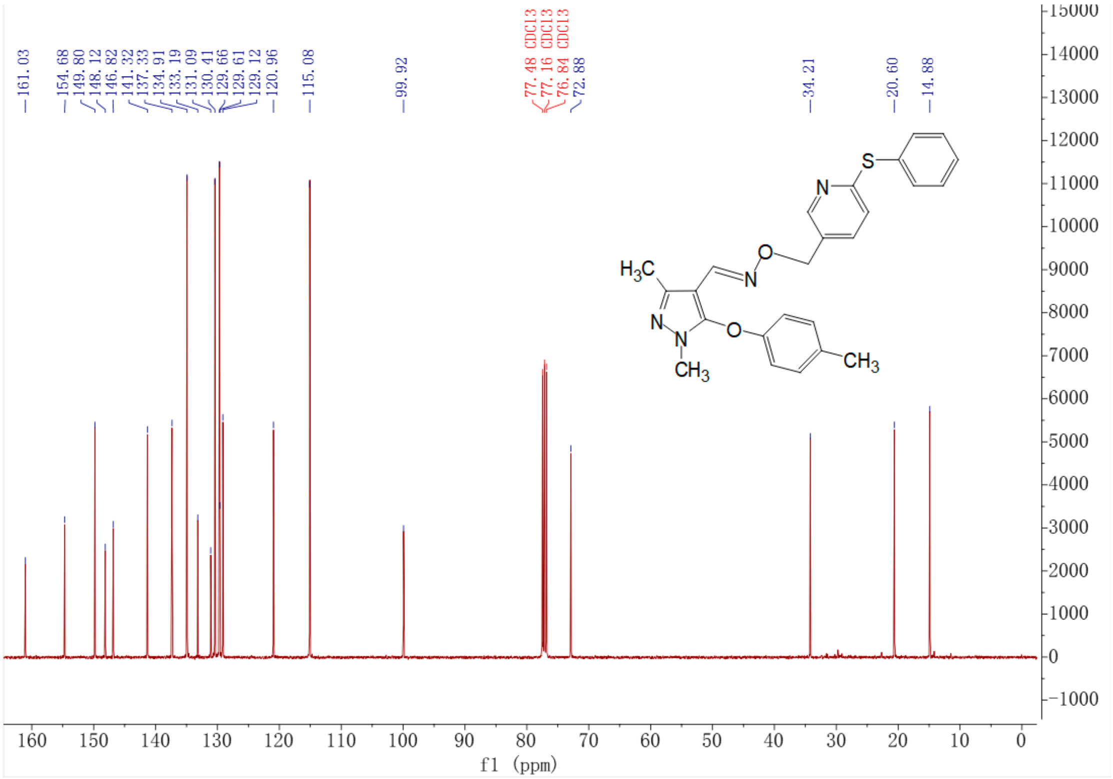

Figure S71. ^{13}C-NMR of compound **8x** (101 MHz, $CDCl_3$)

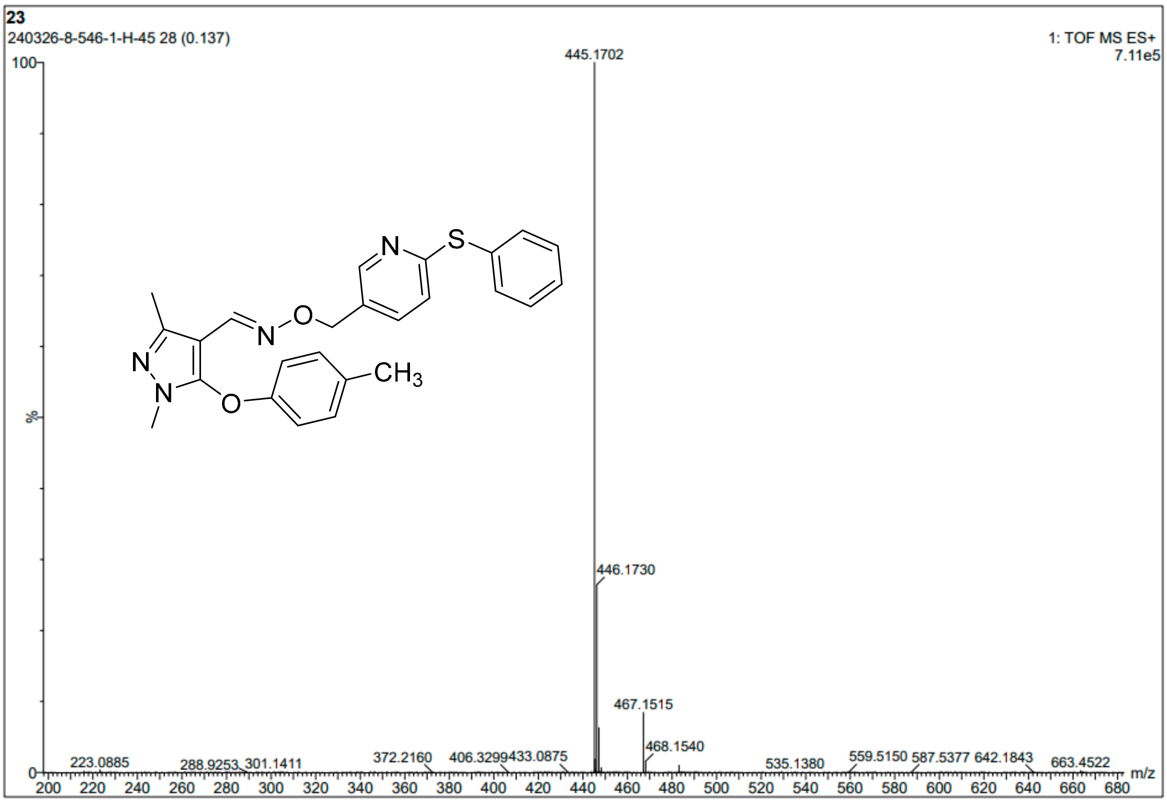

Figure S72. HRMS of compound **8x**

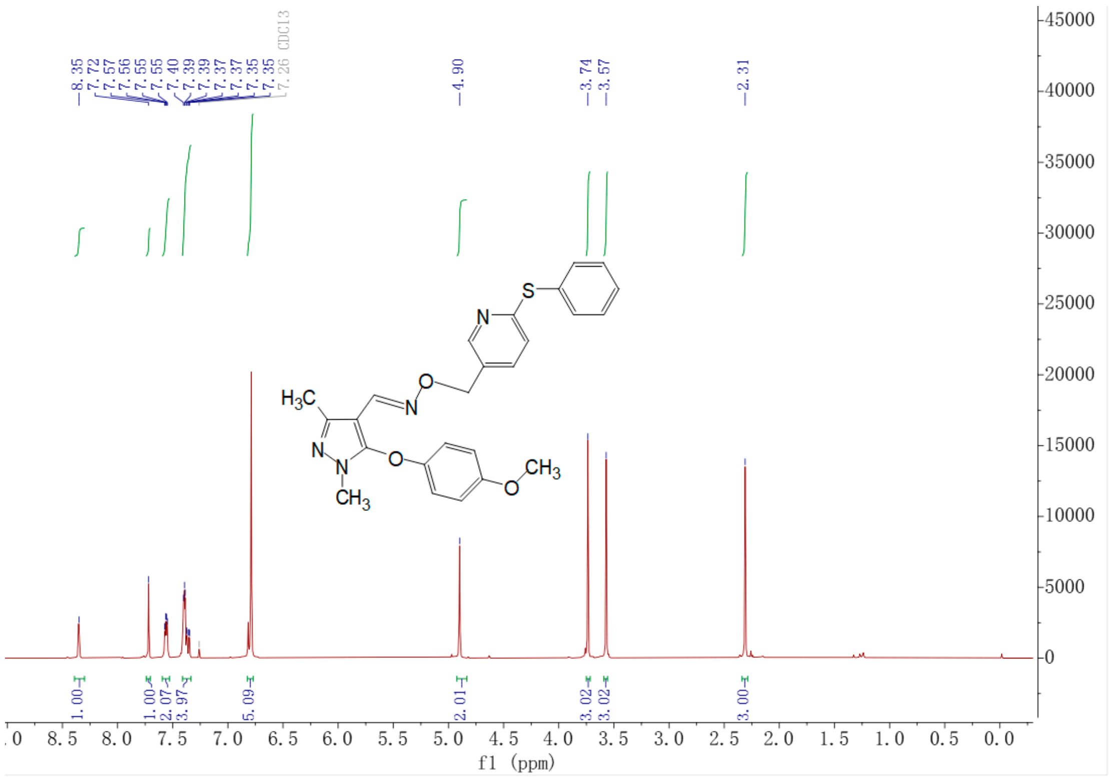

Figure S73. ^{1}H-NMR of compound **8y** (400 MHz, $CDCl_3$)

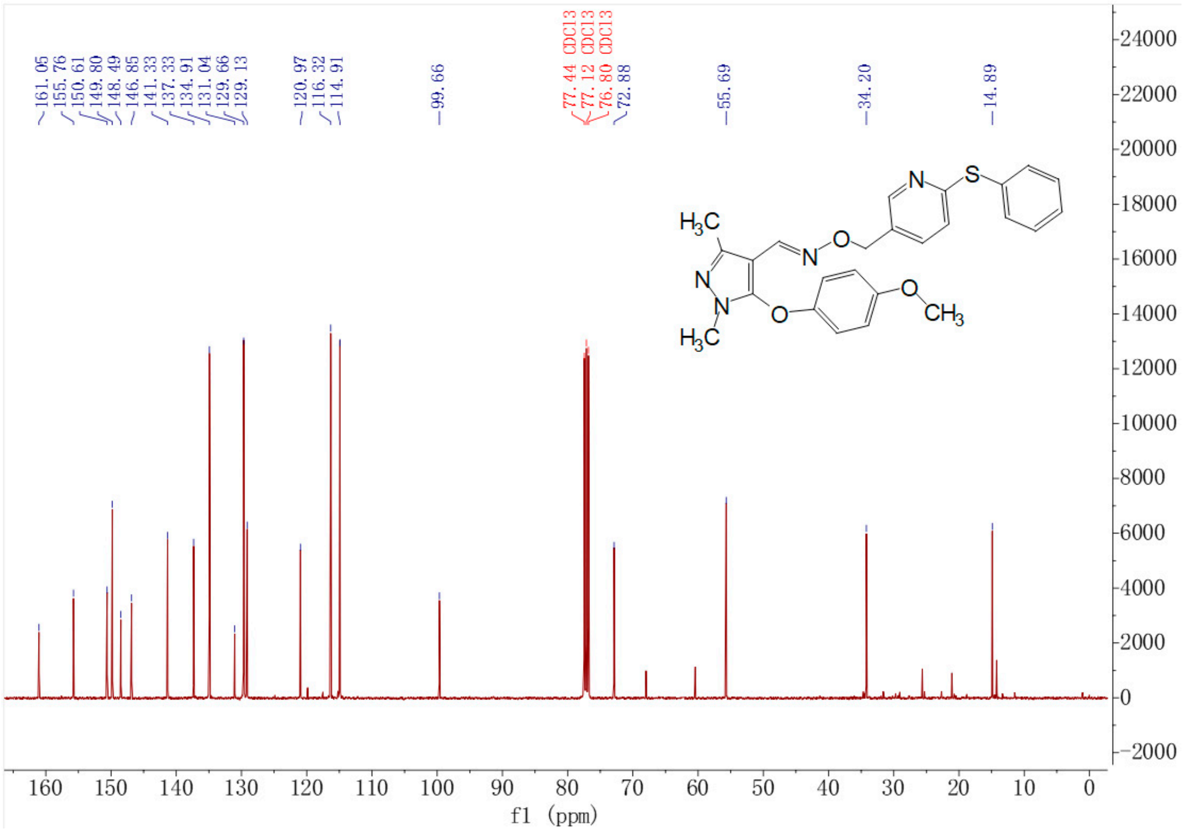

Figure S74. ^{13}C-NMR of compound **8y** (101 MHz, $CDCl_3$)

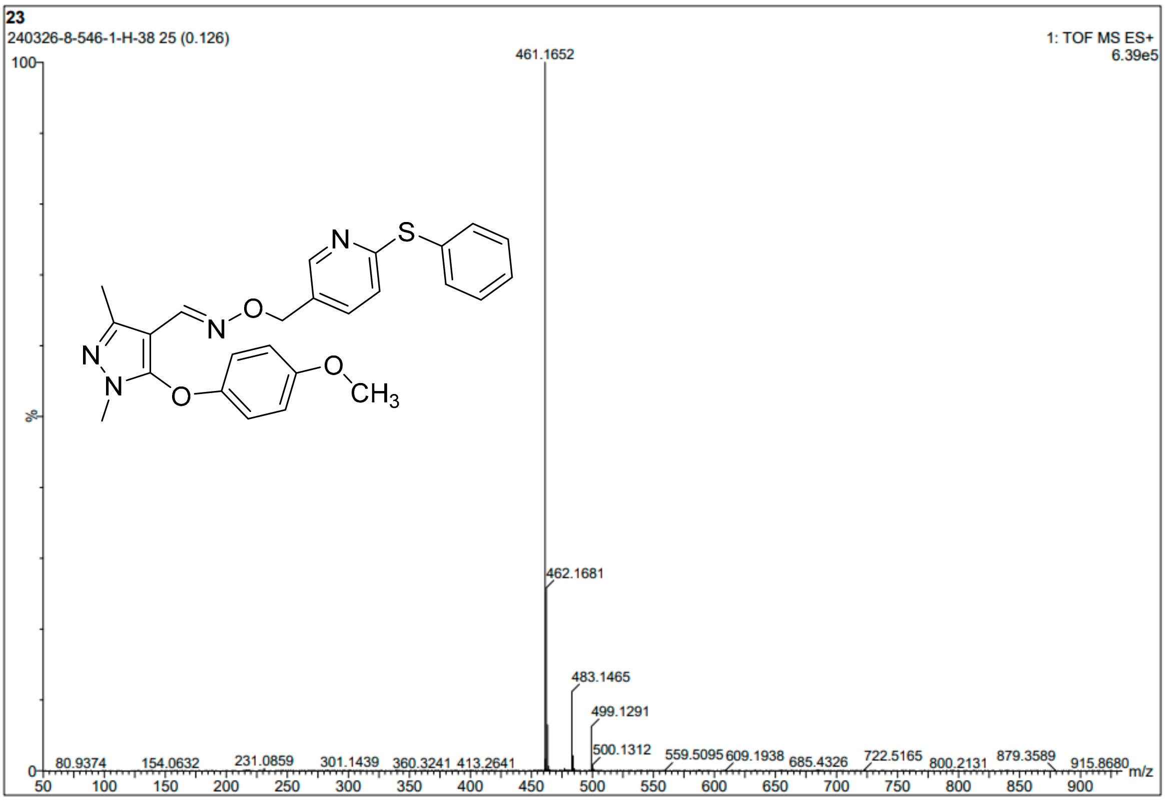

Figure S75. HRMS of compound **8y**

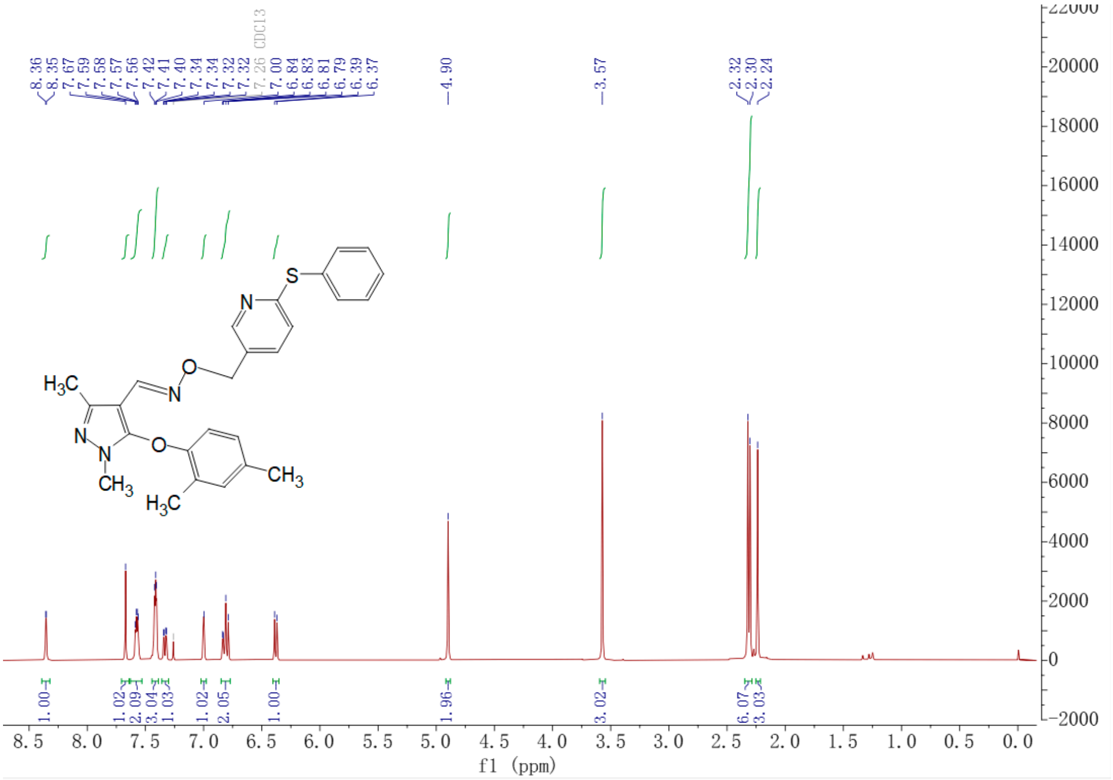

Figure S76. ^{1}H-NMR of compound **8z** (400 MHz, $CDCl_3$)

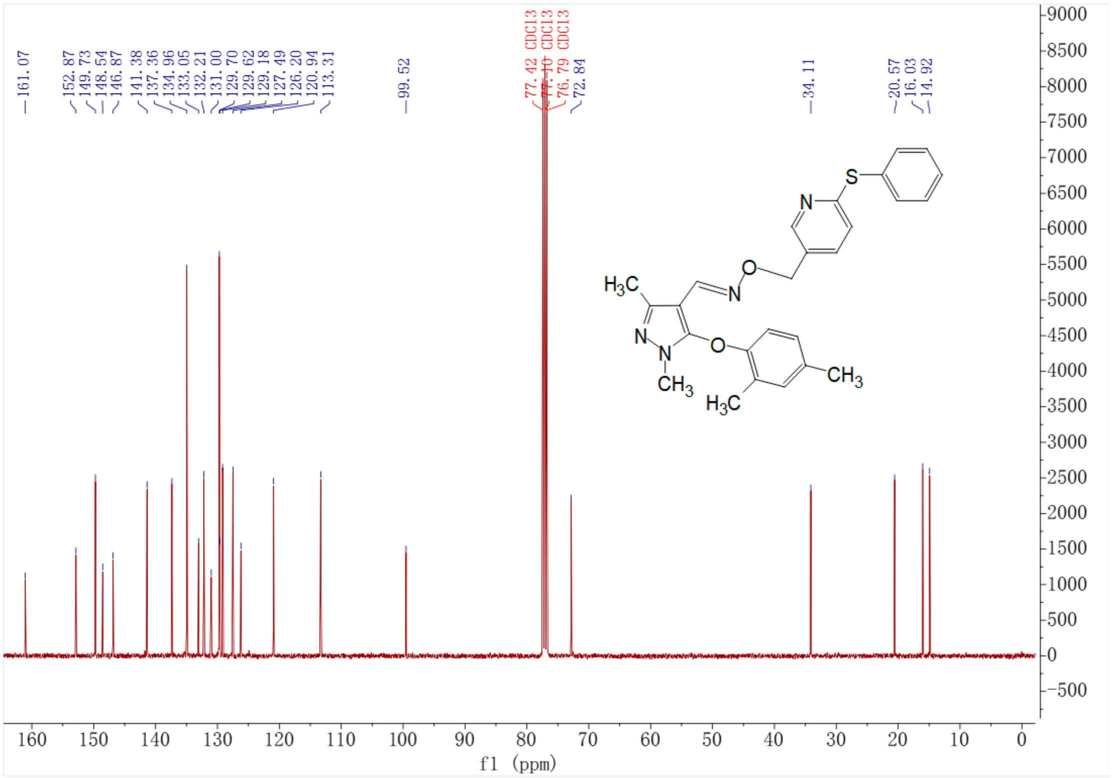

Figure S77. ^{13}C-NMR of compound **8z** (101 MHz, $CDCl_3$)

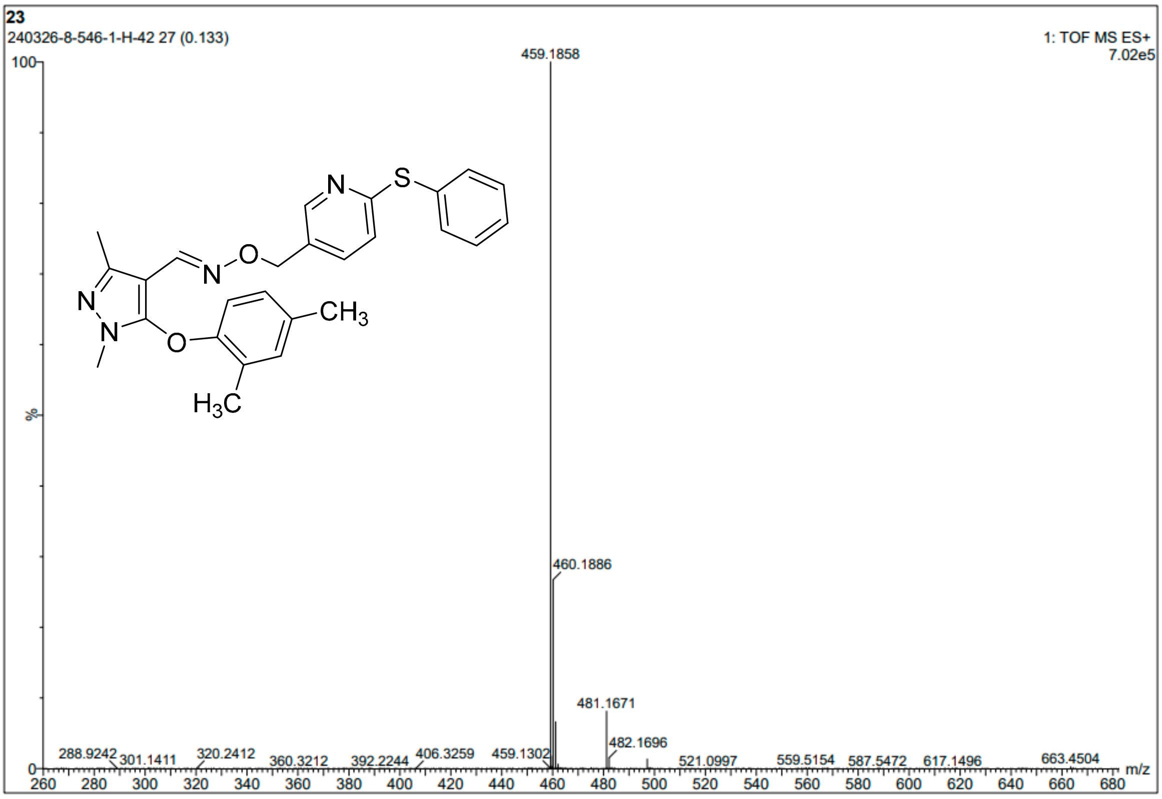

Figure S78. HRMS of compound **8z**

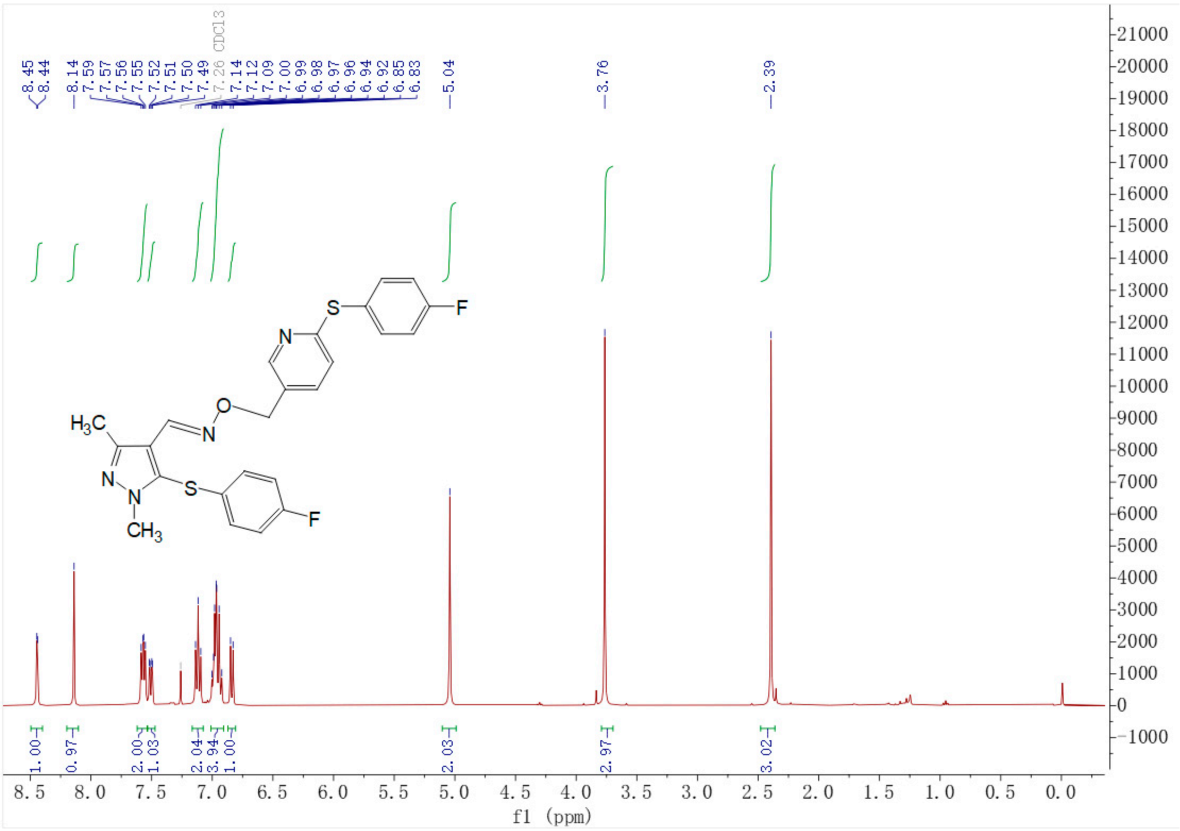

Figure S79. ^{1}H-NMR of compound **11a** (400 MHz, $CDCl_3$)

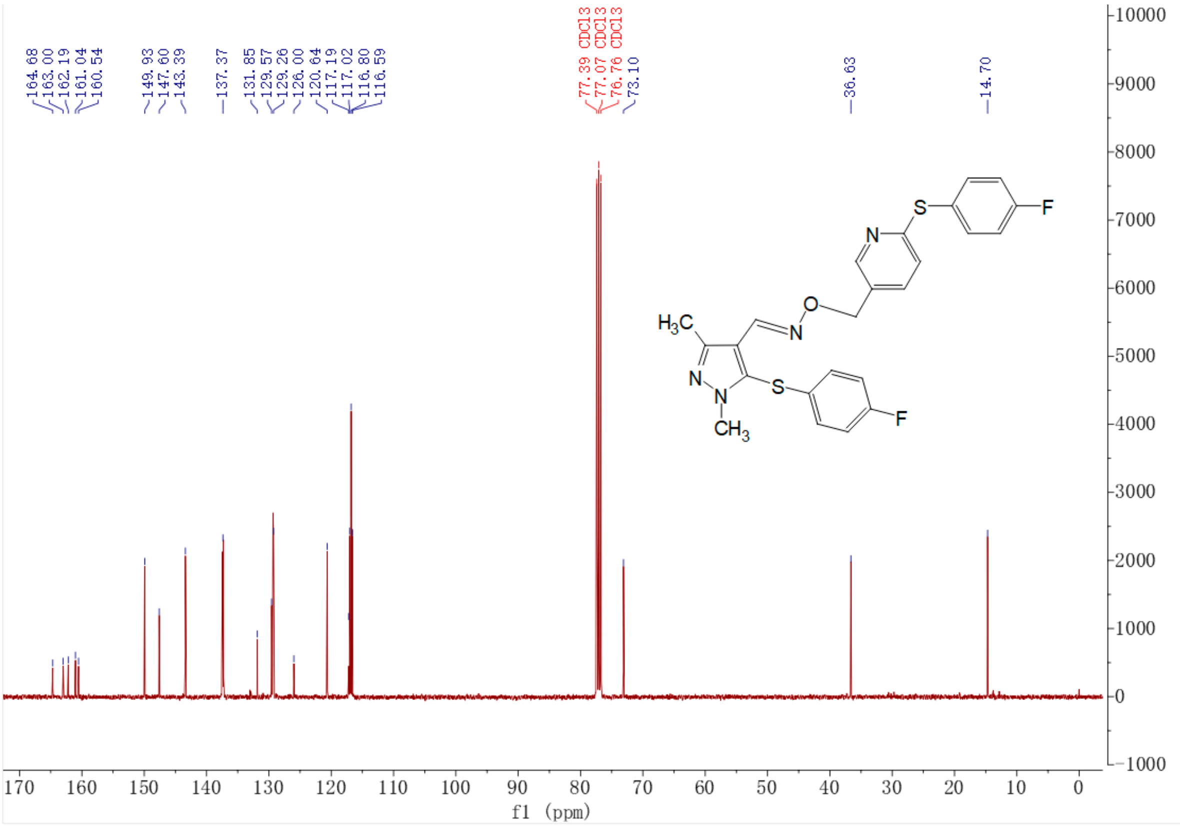

Figure S80. ^{13}C-NMR of compound **11a** (101 MHz, $CDCl_3$)

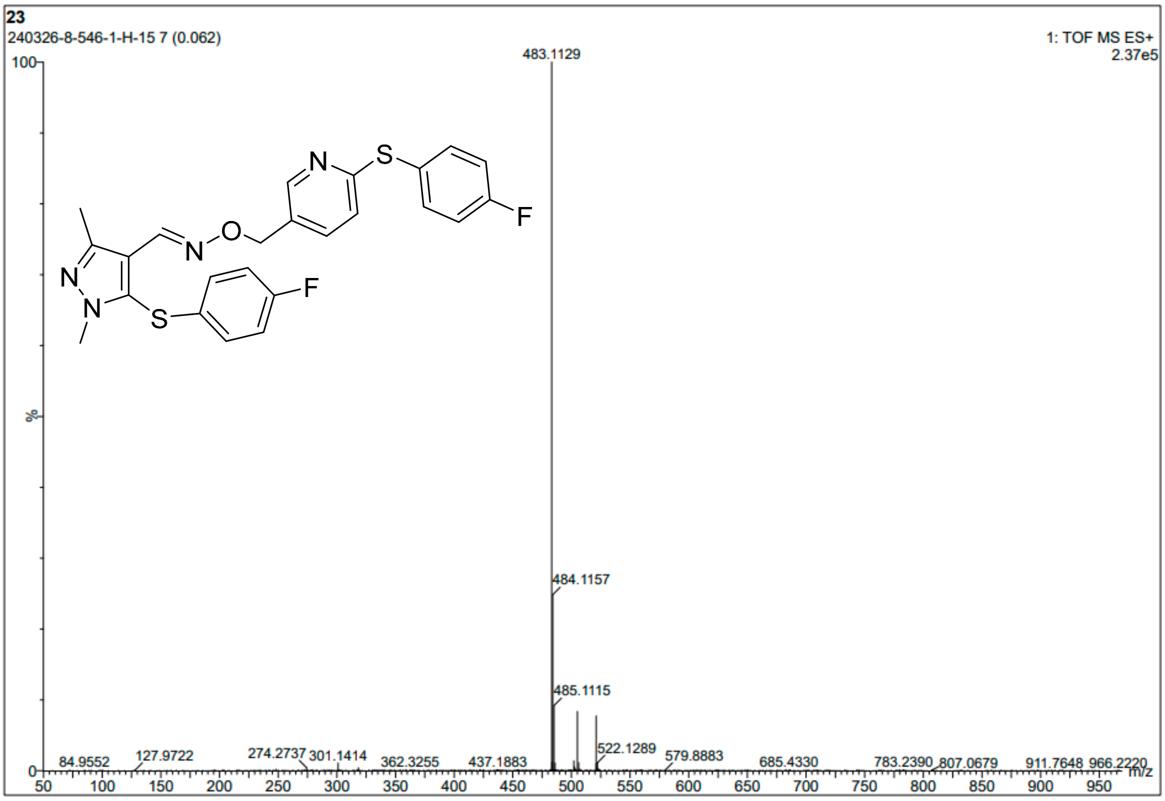

Figure S81. HRMS of compound **11a**

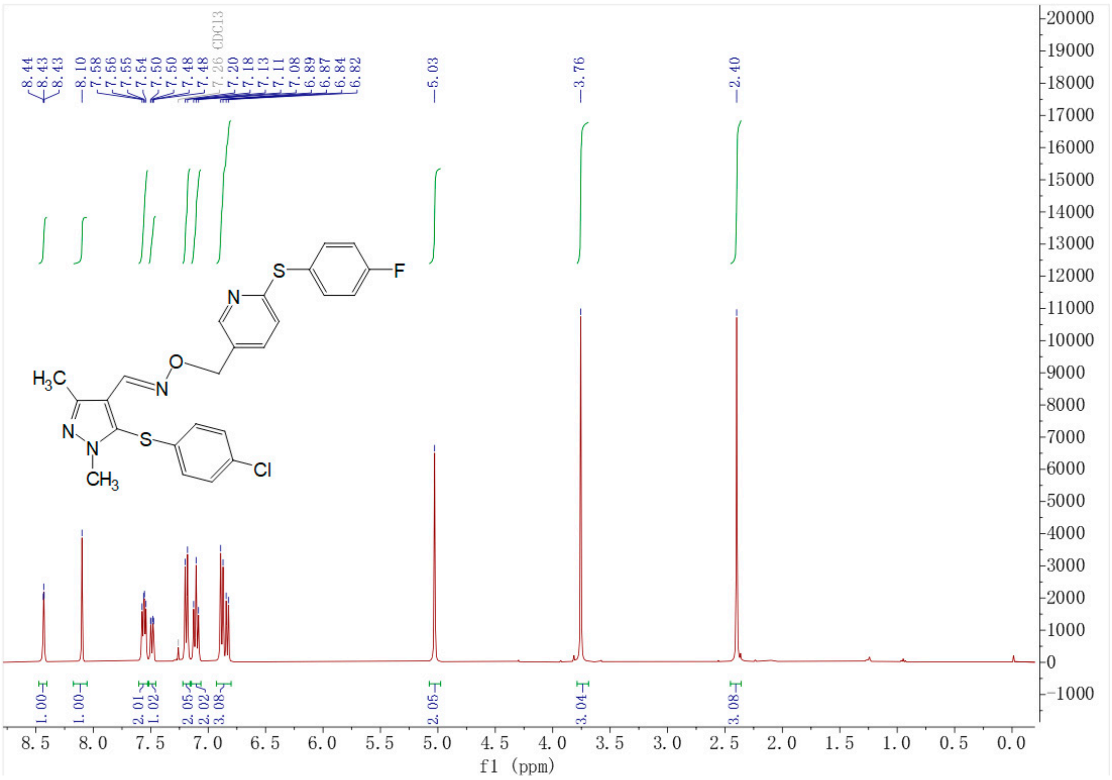

Figure S82. ^{1}H-NMR of compound **11b** (400 MHz, $CDCl_3$)

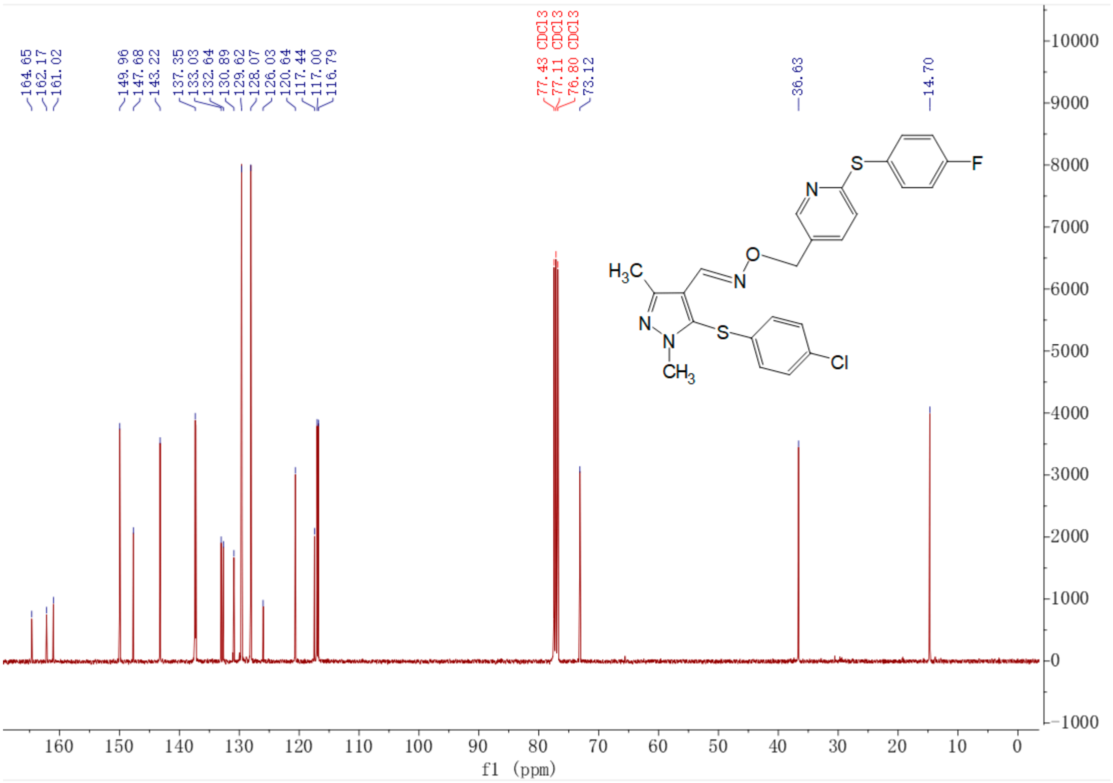

Figure S83. ^{13}C-NMR of compound **11b** (101 MHz, $CDCl_3$)

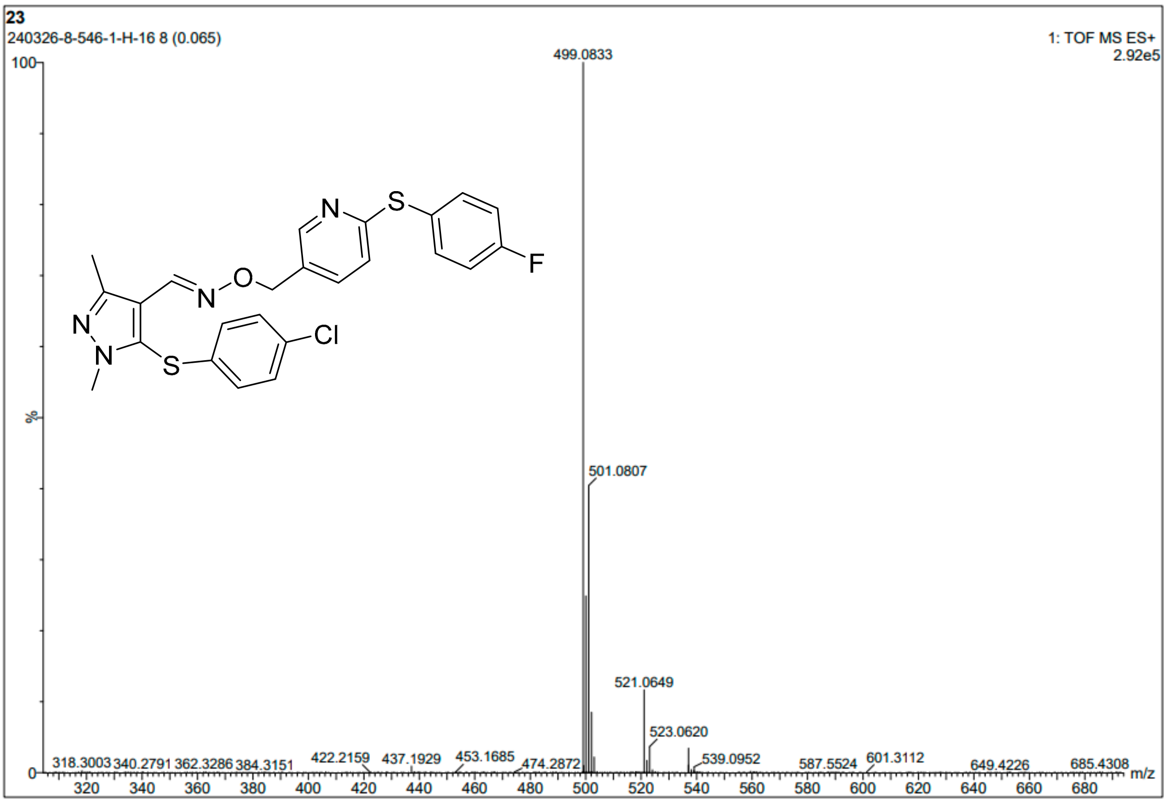

Figure S84. HRMS of compound **11b**

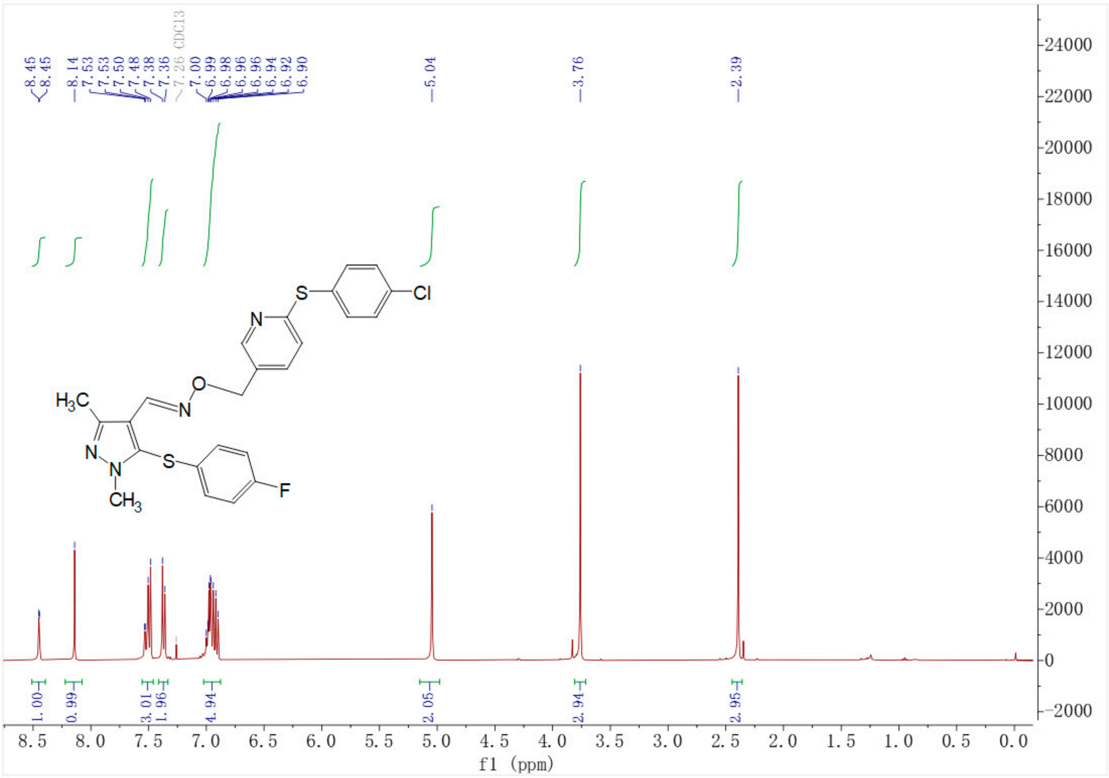

Figure S85. ^{1}H-NMR of compound **11c** (400 MHz, $CDCl_3$)

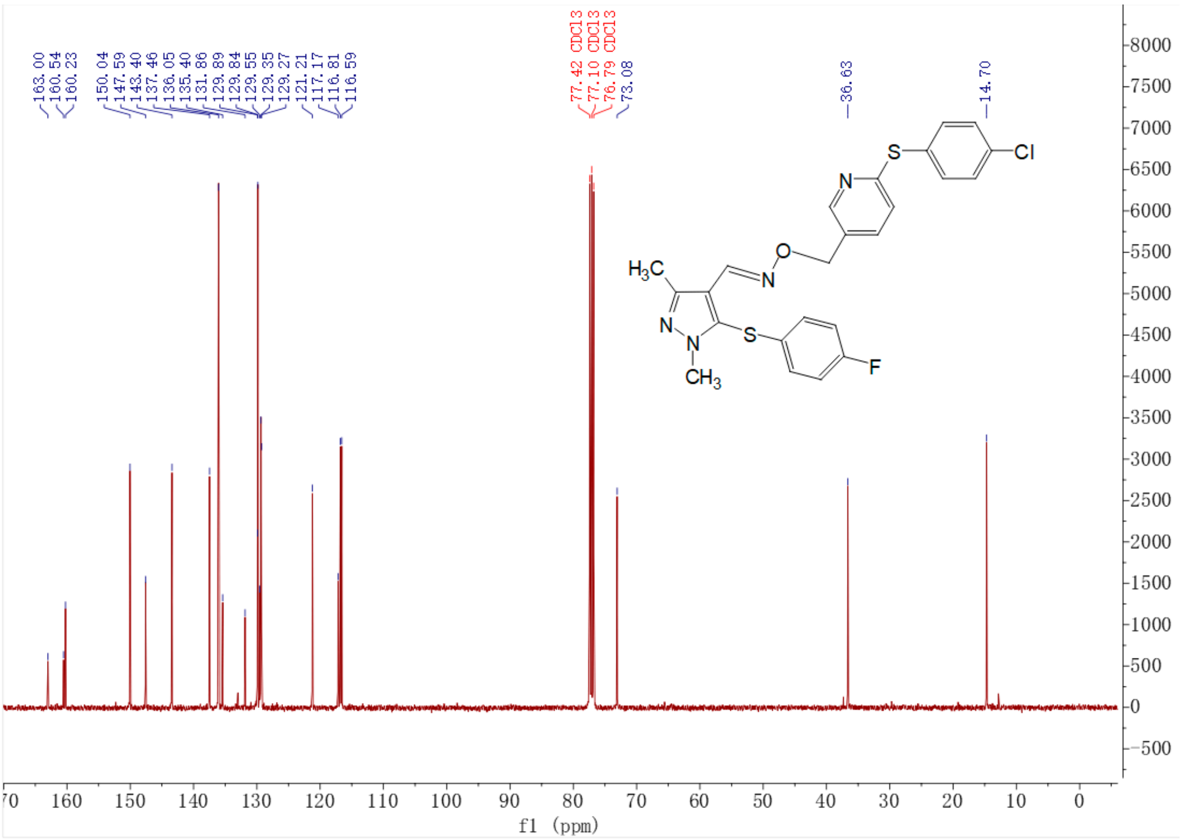

Figure S86. ^{13}C-NMR of compound **11c** (101 MHz, $CDCl_3$)

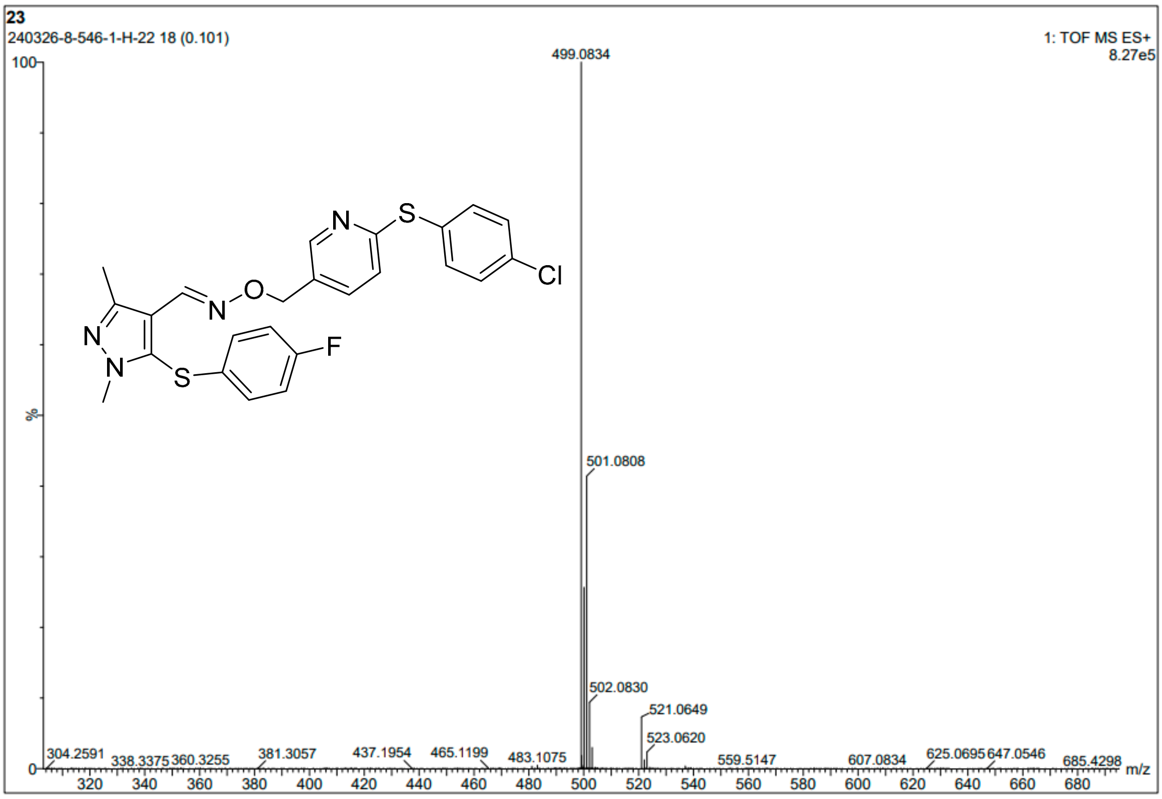

Figure S87. HRMS of compound **11c**

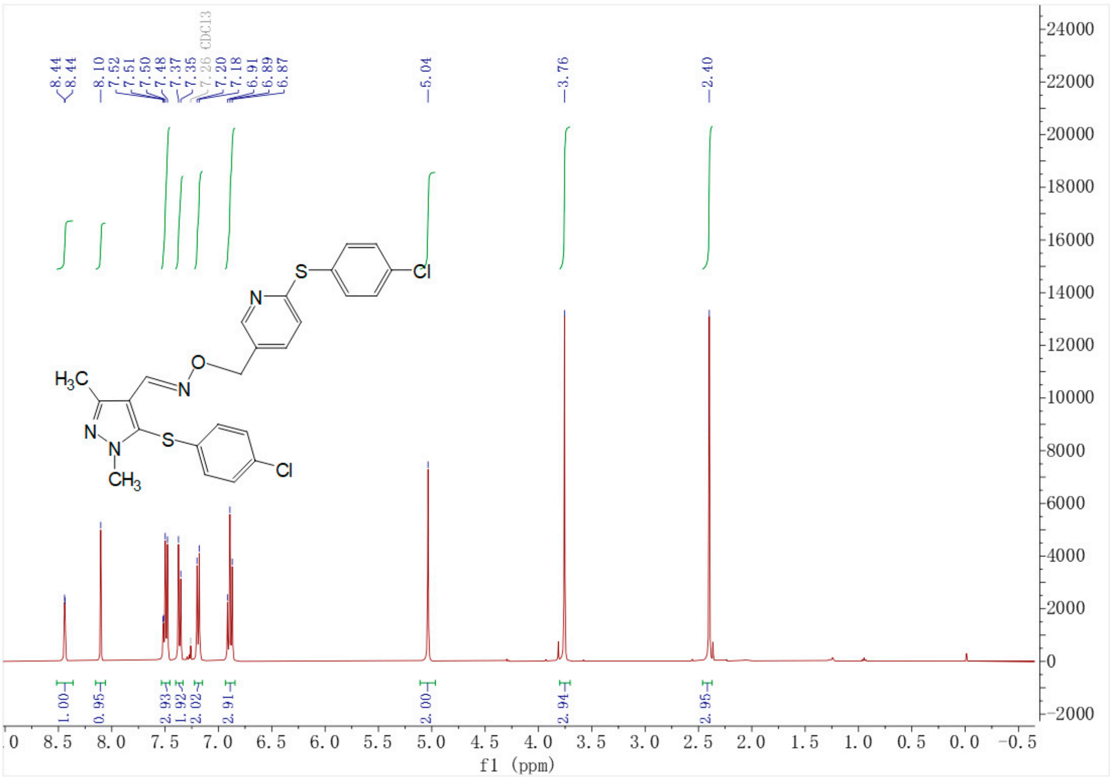

Figure S88. ^{1}H-NMR of compound **11d** (400 MHz, $CDCl_3$)

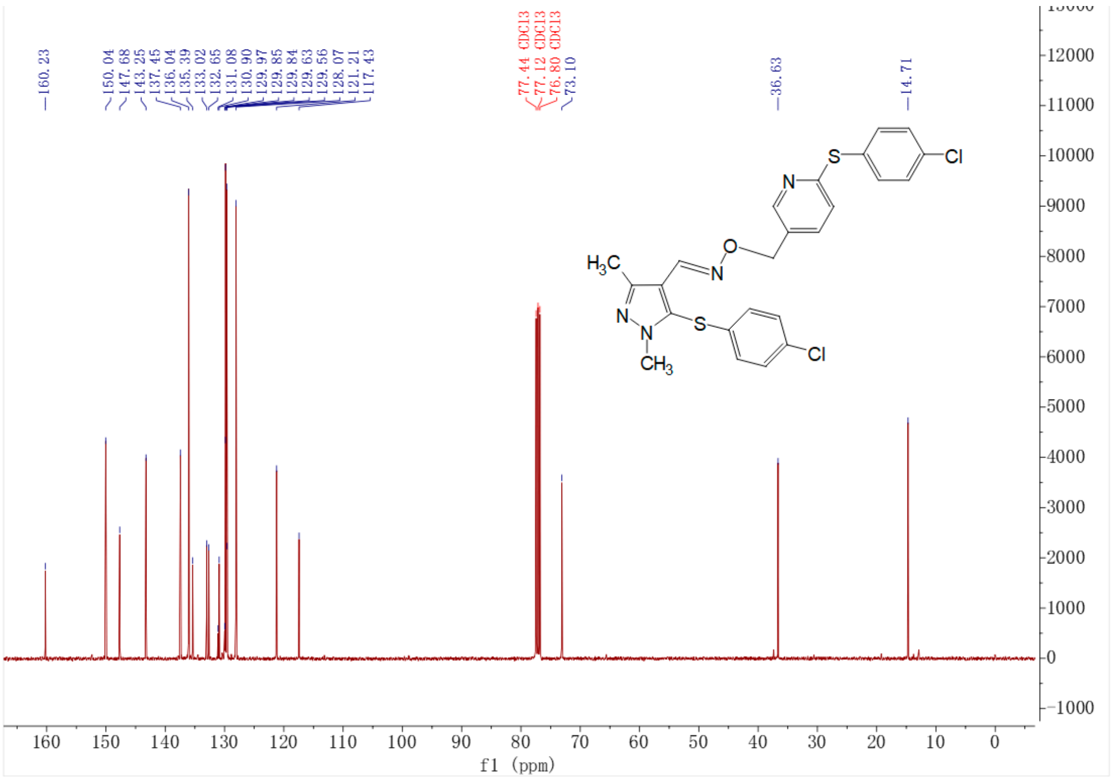

Figure S89. ^{13}C-NMR of compound **11d** (101 MHz, $CDCl_3$)

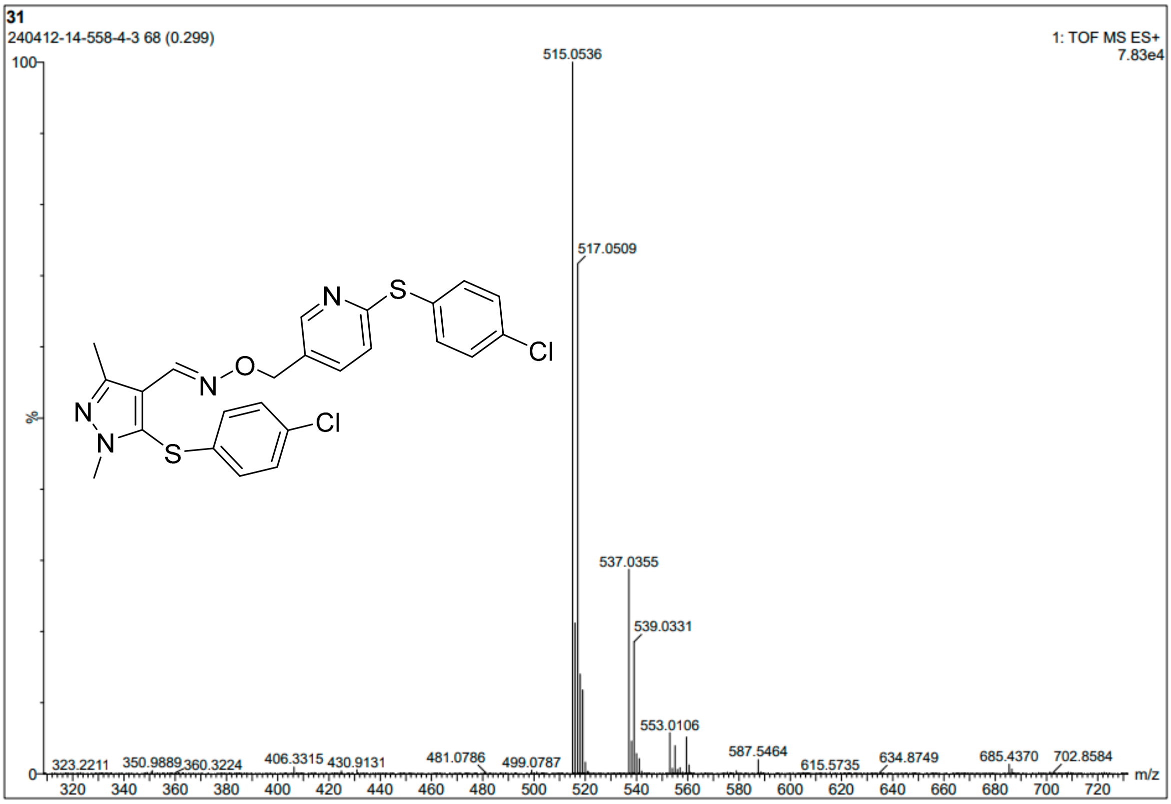

Figure S90. HRMS of compound **11d**

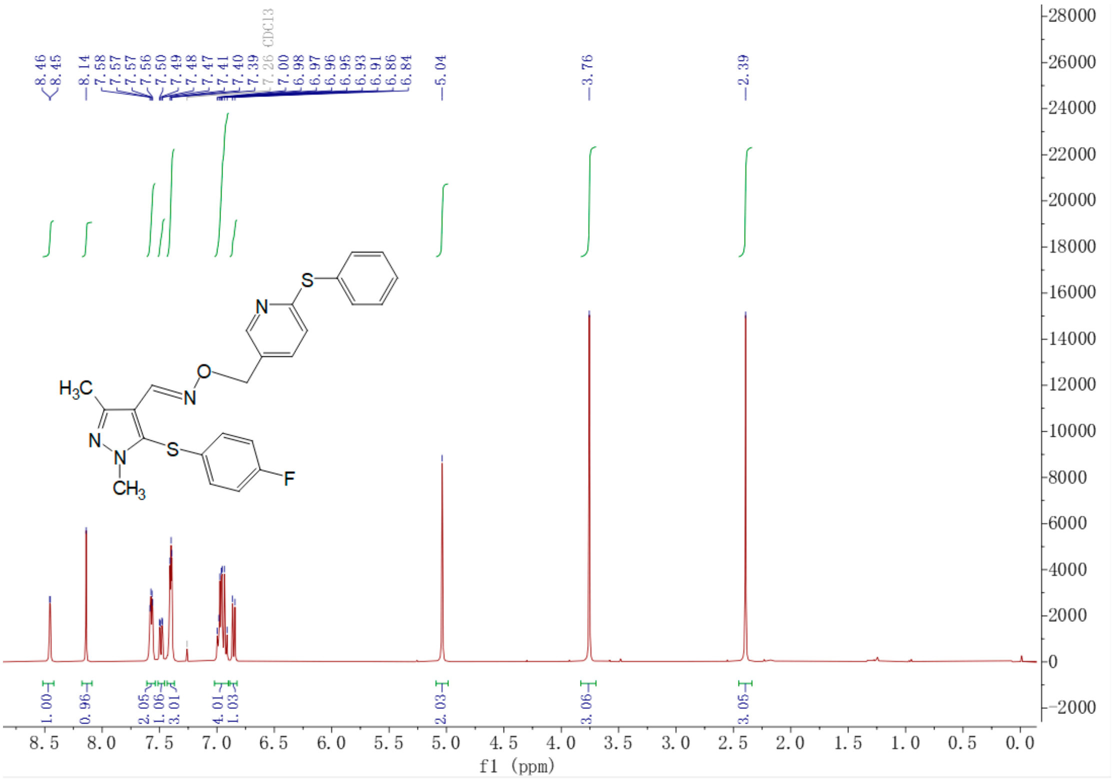

Figure S91. ^{1}H-NMR of compound **11e** (400 MHz, $CDCl_3$)

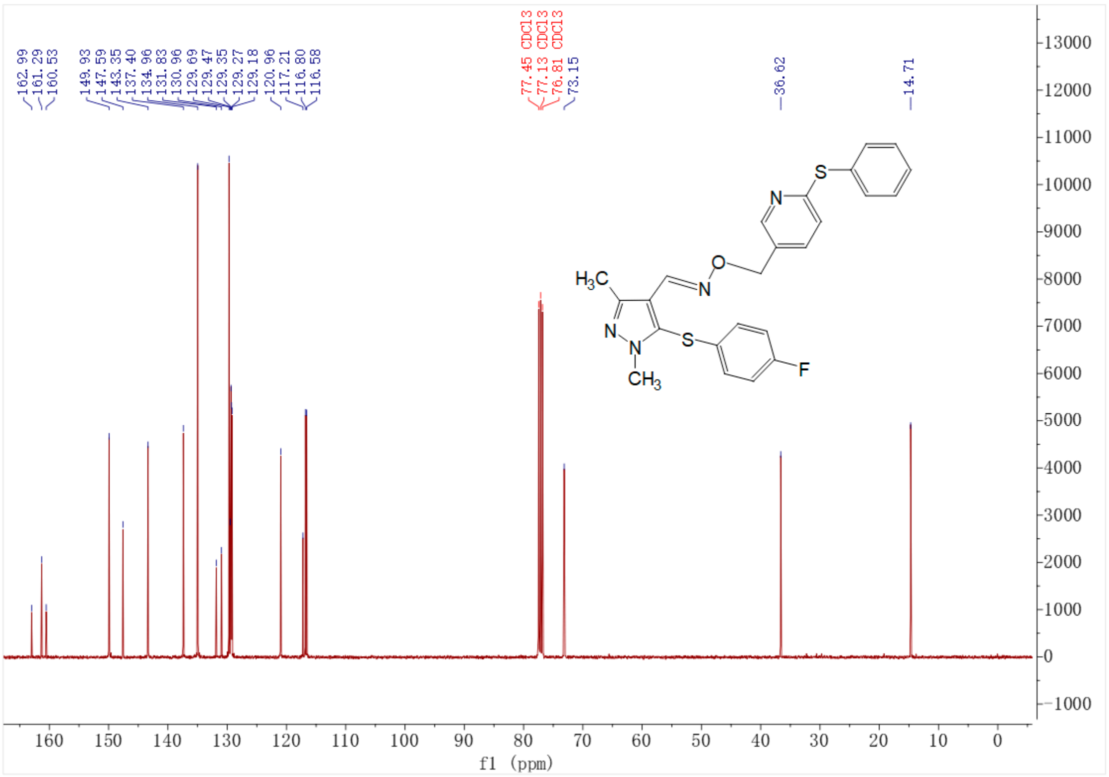

Figure S92. ^{13}C-NMR of compound **11e** (101 MHz, $CDCl_3$)

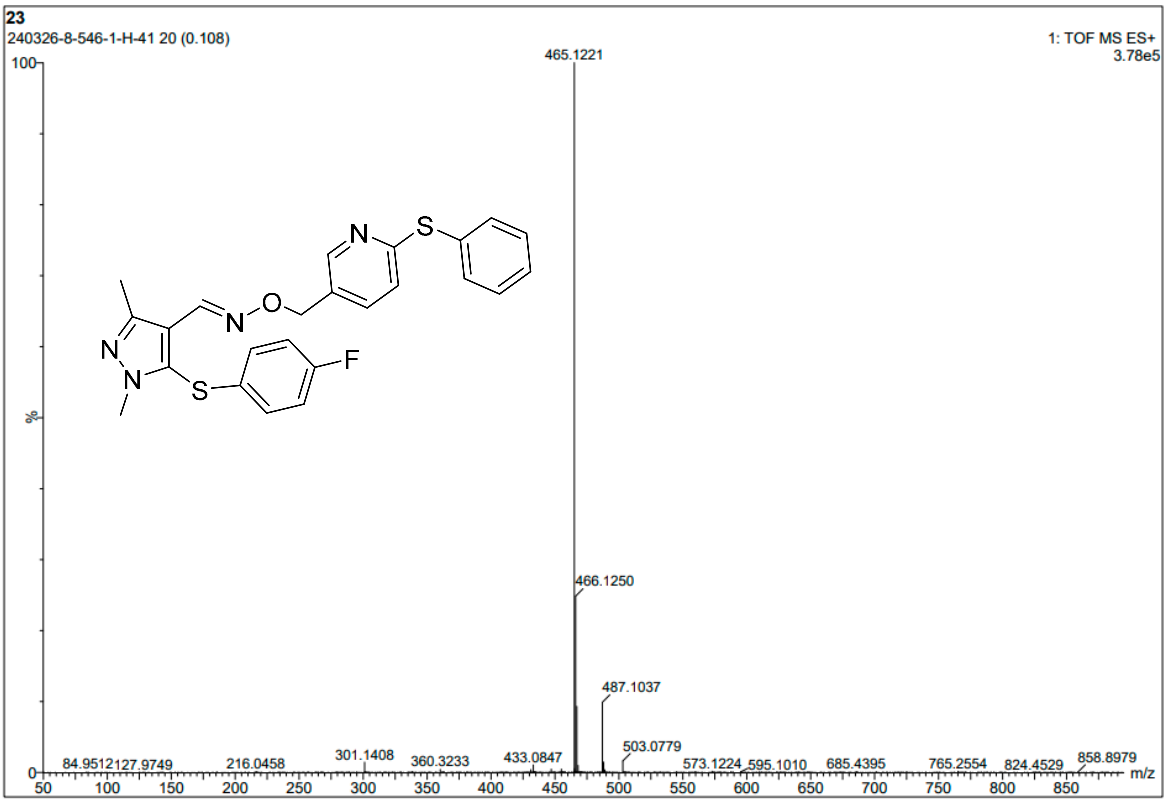

Figure S93. HRMS of compound **11e**

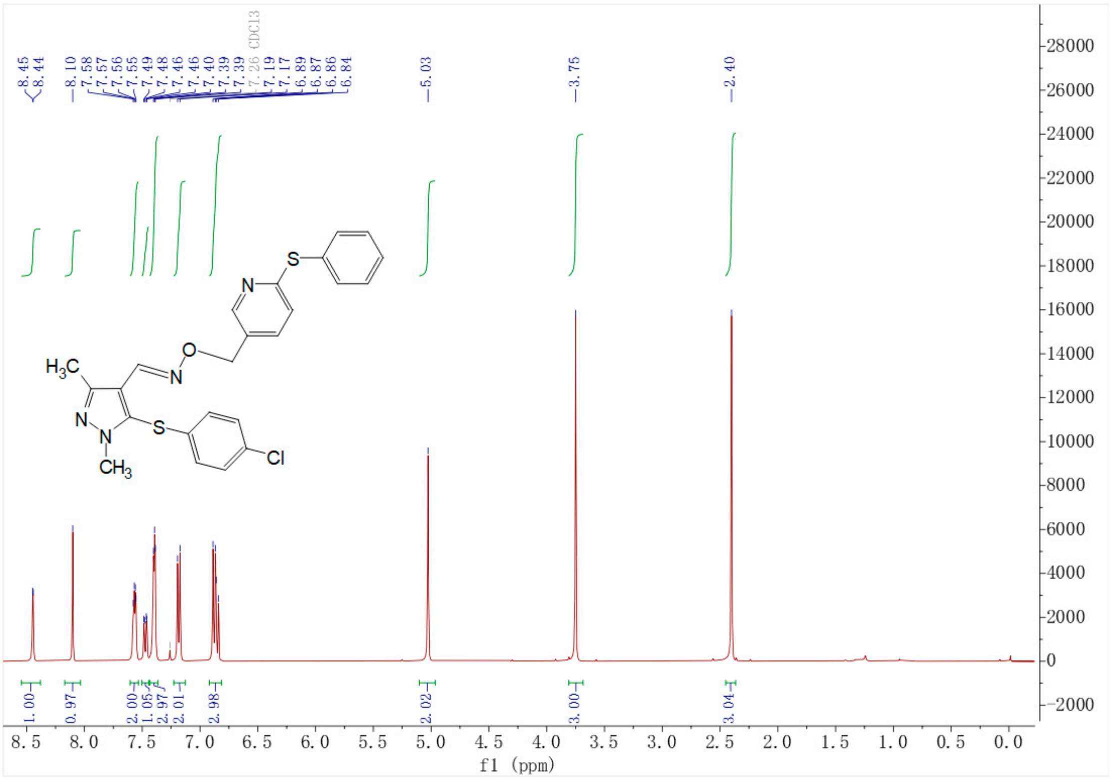

Figure S94. ^{1}H-NMR of compound **11f** (400 MHz, $CDCl_3$)

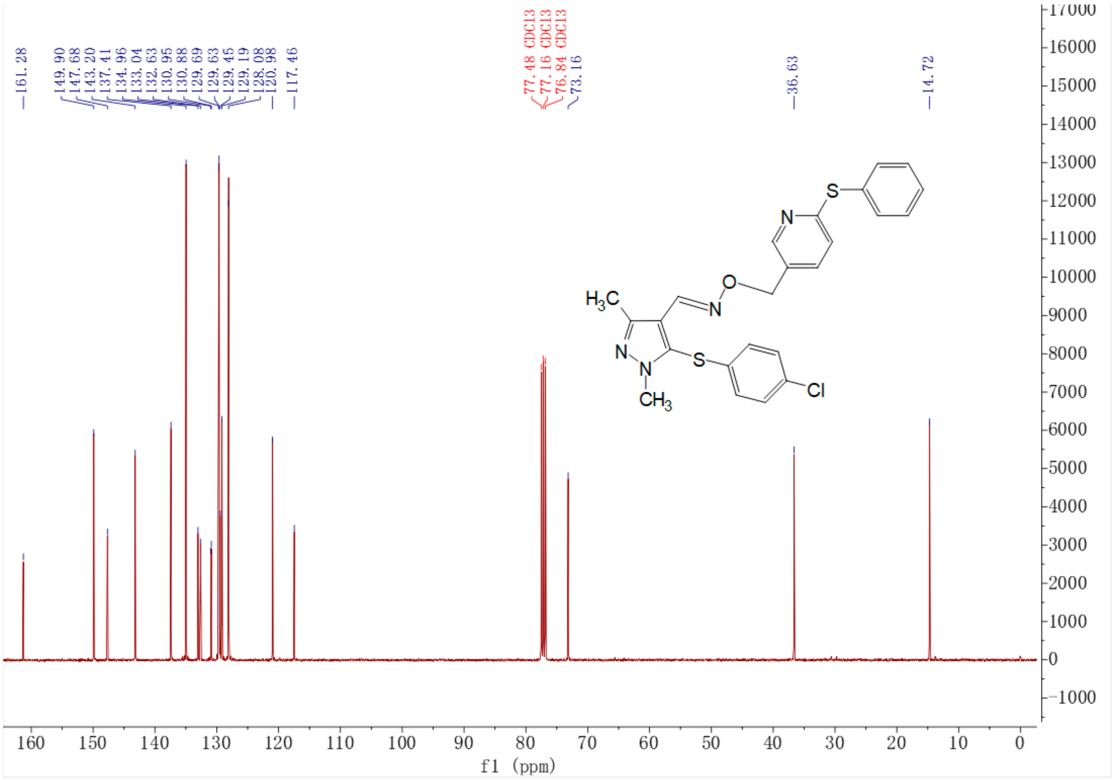

Figure S95. ^{13}C-NMR of compound **11f** (101 MHz, $CDCl_3$)

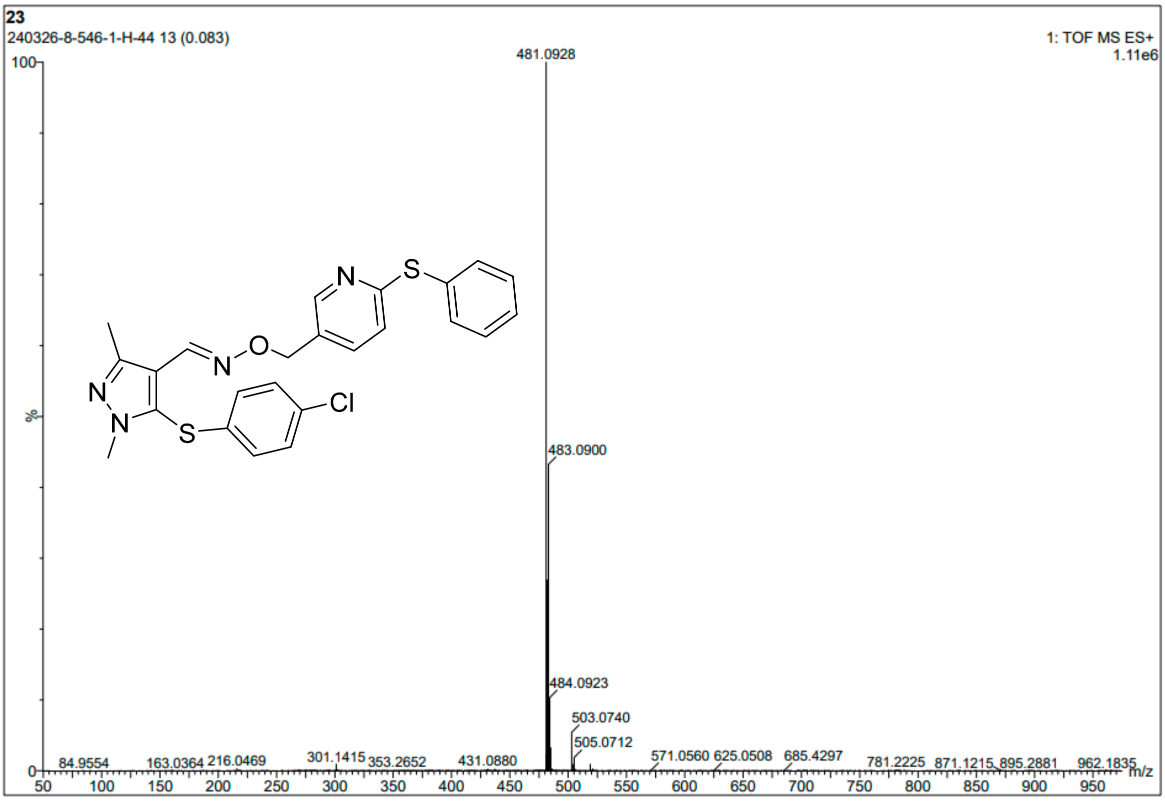

Figure S96. HRMS of compound **11f**
